# Rewriting and suppressing UMLS terms for improved biomedical term identification

**DOI:** 10.1186/2041-1480-1-5

**Published:** 2010-03-31

**Authors:** Kristina M Hettne, Erik M van Mulligen, Martijn J Schuemie, Bob JA Schijvenaars, Jan A Kors

**Affiliations:** 1 Department of Medical Informatics, Erasmus University Medical Center, Rotterdam, the Netherlands; 2 Department of Health Risk Analysis and Toxicology, Maastricht University, Maastricht, The Netherlands; 3 Collexis Holdings Inc, Columbia SC, USA

## Abstract

**Background:**

Identification of terms is essential for biomedical text mining.. We concentrate here on the use of vocabularies for term identification, specifically the Unified Medical Language System (UMLS). To make the UMLS more suitable for biomedical text mining we implemented and evaluated nine term rewrite and eight term suppression rules. The rules rely on UMLS properties that have been identified in previous work by others, together with an additional set of new properties discovered by our group during our work with the UMLS. Our work complements the earlier work in that we measure the impact on the number of terms identified by the different rules on a MEDLINE corpus. The number of uniquely identified terms and their frequency in MEDLINE were computed before and after applying the rules. The 50 most frequently found terms together with a sample of 100 randomly selected terms were evaluated for every rule.

**Results:**

Five of the nine rewrite rules were found to generate additional synonyms and spelling variants that correctly corresponded to the meaning of the original terms and seven out of the eight suppression rules were found to suppress only undesired terms. Using the five rewrite rules that passed our evaluation, we were able to identify 1,117,772 new occurrences of 14,784 rewritten terms in MEDLINE. Without the rewriting, we recognized 651,268 terms belonging to 397,414 concepts; with rewriting, we recognized 666,053 terms belonging to 410,823 concepts, which is an increase of 2.8% in the number of terms and an increase of 3.4% in the number of concepts recognized. Using the seven suppression rules, a total of 257,118 undesired terms were suppressed in the UMLS, notably decreasing its size. 7,397 terms were suppressed in the corpus.

**Conclusions:**

We recommend applying the five rewrite rules and seven suppression rules that passed our evaluation when the UMLS is to be used for biomedical term identification in MEDLINE. A software tool to apply these rules to the UMLS is freely available at http://biosemantics.org/casper.

## Background

Biomedical text mining has been shown to be valuable for diverse applications in the domains of molecular biology, toxicogenomics, and medicine. For example, it has been used to functionally annotate gene lists from microarray experiments [1-4], create literature-based compound profiles [5], generate medical hypotheses [6,7], find new uses for old drugs [8-10], and measure protein similarity [11,12]. The identification of biomedical terms in natural language is essential for biomedical text mining. The process of term identification consists of three tasks: term recognition, term classification and term mapping [13,14]. Approaches to term identification generally fall into three categories: lexicon-based systems, rule-based systems, and statistics-based systems making use of different machine learning techniques [15]. All approaches have their disadvantages: lexicon-based systems are dependent on fast updates and large coverage of the underlying lexicons; to craft the rules for a rule-based system is time consuming and requires a high level of domain knowledge, and statistics-based systems need annotated corpora to train the classifiers. Term mapping, in which terms are linked to reference data sources, is the last step in the term identification process. Term mapping is only possible using lexicon-based term identification and is the focus of this paper (for comprehensive reviews on term identification see for example [13-17]). In addition, the lexicon-based approach deals with general medical terms for which it is difficult to design general matching patterns that are used by rule-based systems. It provides information concerning the semantic relations between terms and supports synonym and referent data source mapping, which is not possible using rule-based or statistically-based term identification. Specifically, we use the Unified Medical Language System (UMLS) meta-thesaurus provided by the U.S National Library of Medicine (NLM) [18]. The 2007AA edition of the UMLS contains more than 1.3 million concepts and 6.4 million terms referring to these concepts from more than 100 different vocabularies. These vocabularies cover different aspects of the biomedical field and have been developed for such different purposes as disease and procedure coding, adverse event reporting, literature indexing, billing, and gene function identification. NLM checks terms from different vocabularies for synonymy, assigns a unique concept identifier (CUI) and assigns concepts to one or more semantic types from the UMLS Semantic Network.

Naturally, the usefulness of the lexicon-based approach depends on the coverage of terms in the vocabulary for the particular domain and how well the terms are suited for natural language processing. The UMLS is not primarily intended as a resource for text mining, so not all of its terms are suitable for this purpose. For example, terms for coding of concepts can include specialized syntax (e.g., brackets) that is not suitable for text mining solutions ("undesired terms"). The following definition of a term in the UMLS can be found in the UMLS Glossary [19]: "A word or collection of words comprising an expression. In the Metathesaurus, a term is the class of all strings that are lexical variants (made singular and normalized to case) of each other". his definition allows for expressions that are not terms according to certain theories of terminology, in which terms are expressions that are actually used in domain-specific communication [20-22]. In fact, the UMLS abounds of expressions that are not expected to occur in any written or oral communication but are intended to precisely paraphrase the exact meaning of a concept. This has been illustrated by, for example, Srinivasan et al. [23], who found that by using normalized matching (i.e. ignoring case variation, punctuation, possessive markers, inflectional variation and word order) only a total of 34.3% of the 1,451,824 terms in the January 2002 version of the UMLS (non-English terms and terms with a suppressible term type excluded) could be found in a 11.5 million MEDLINE abstract corpus. McCray et al [24] could only find 10% of the UMLS terms when using a smaller corpus of 439,741 MEDLINE abstracts (UMLS 2001 version, 1,397,429 English terms, string features retained except for case variation). The lower match result in comparison with Srinivasan et al. might be explained by the difference in the matching methods, the UMLS version, and by the smaller corpus used. McCray et al. [24,25] also investigated the nature of the strings in the UMLS and evaluated them for their use in natural language processing. The investigation resulted in a number of properties that could be used to filter unwanted strings from the UMLS. Rogers and Aronson [26] identified a number of filtering rules and term types which help in filtering the UMLS for the update of the MetaMap program [27].

This paper is inspired by McCray et al. [24,25] and Rogers and Aronson [26] in that we aim to make the UMLS more useable for text-mining purposes. We do this by removing and adding synonyms to the UMLS, which are supposed to increase the accuracy and efficiency of biomedical term identification using the UMLS. We manually evaluate the impact of the rules on a MEDLINE corpus.

## Methods

The rewrite rules were implemented to increase the recall of UMLS concepts in text. The suppression rules on the other hand were implemented to rid the UMLS of terms that are undesired when it comes to term identification either because they affect the precision of the term identification, e.g. the synonym "2" for the term "clinical class", the synonym "EC 2.7.1.-" for the concept "human CDC7 protein", or because they affect the efficiency of the term identification, i.e. long and vague terms that are unlikely to be found in text such as the term "poisoning by other and unspecified drugs and medicinal substances" or terms that are useless for concept identification such as the concept with the single term "WHILE". We applied the rules to the 2007AA version of the UMLS in UTF8 coding and then indexed citations from the MEDLINE database (1965-2007) (we refer to this as "the corpus" in the rest of the paper). Finally, the identified rewritten terms were manually assessed for their correspondence to the original UMLS terms and the identified suppressed terms were manually assessed for their usefulness for automatic text mining purposes. A detailed description of the procedure follows.

### UMLS extraction

The UMLS 2007AA version was downloaded from the UMLS knowledge source server [28] and installed locally using the MetamorphoSys tool provided by NLM for customizing the UMLS. The default settings in MetamorphoSys were used to create the UMLS subset, using the option to include all vocabularies in the English language. Strings marked as suppressible by the NLM as well as strings longer than 255 characters were not included in the analysis. This approach resulted in 2,844,004 strings, based on the String Unique Identifier (SUI) field in the UMLS. These strings belonged to 1,294,936 concepts, based on the CUI field in the UMLS. Duplicate strings within a concept were removed by comparing strings after conversion to lower case and removal of punctuation; 2,696,820 strings remained and these are henceforth referred to as "terms".

### Corpus creation

All MEDLINE citations (title and abstract) available at the time of this study, with publication dates ranging from January 1965 to December 2007 (17,674,805 citations, of which 9,446,335 have an abstract) were used as a test corpus.

### Creation of rules

A set of nine rewrite rules and eight suppression rules were given. A description of the rules together with motivation and differences in comparison to original source (when applicable) is provided below. In order to avoid introducing duplicates and homonyms when applying the rewrite rules, a new term was not added to the concept if it could already be found among the synonyms for that concept or any other concept (case insensitive matching after removal of punctuation).

#### 1) Rewrite rules

***Syntactic inversion ***[24,26]: add syntactic inversion of term if a term contains a comma followed by a space and does not contain a preposition or conjunction (e.g. "Failure, Renal"). We added the condition that only one such pattern of a comma followed by a space is to be found in a term for the rule to be executed.

***Possessives ***[26]: remove the possessive "'s" at the end of a word (e.g. "Alzheimer's disease") and add the rewritten term.

***Short form/long form ***[29]: add short form and long form of term (e.g. "Selective Serotonin Reuptake Inhibitors (SSRIs)"). Schwartz and Hearst's algorithm [29] achieved 96% precision and 82% recall on a standard test collection, which was as good as existing approaches at the time [29] and still competitive according to recent comparison studies [30,31]. An advantage of the algorithm is that, unlike other approaches, it does not require any training data. Two extra conditions were added to the original rule by Schwartz and Hearst: 1) the short form must be found at the end of the term, and 2) the first letter of the short form should be the same as the first letter of the long form. These conditions were added in order to adjust the rule to extract abbreviations from a dictionary instead of from biomedical text.

**A*ngular brackets ***[26]: remove expressions within angular brackets anywhere in a term. This pattern was previously used in the UMLS to denote polysemy or homonymy of a term, i.e. a term having different meanings. Terms having this property still exist in the UMLS, even though the property is not assigned to new terms. We have adjusted the rule to remove expressions within angular brackets anywhere in a term since these expressions usually contain meta-information about a term, which is unlikely to be found in text (e.g. "Chondria <beetle>").

***Semantic type***: remove expressions within parentheses that match the list of semantic types in the UMLS (e.g. "Surgical intervention (finding)"). This rule was developed by our group based on the observation that the semantic type to which the term belongs to is often added as meta-information about the term.

***Non-essential parentheticals ***[24,26] has been split into four rules in order to make the error analysis more transparent:

1. ***Begin parentheses***: remove expressions within parenthesis at the beginning of a term (e.g. (protein) methionine-R-sulfoxide reductase)

2. ***Begin brackets***: remove expressions within brackets at the beginning of a term (e.g. [V] Alcohol use)

3. ***End parentheses ***removes expressions within parenthesis at the end of a term (e.g. flagellar filament (sensu Bacteria))

4. ***End brackets ***removes expressions within brackets at the end of a term (e.g. Gluten-free foods [generic 1])

In addition, we have added the condition that the rule does not apply to terms belonging to the semantic group Chemicals & Drugs. The reason for this condition is that chemical expressions by nature often contain both brackets and parentheses at the beginning or end of a term.

#### 2) Suppression rules

***Short token ***[24,26]: remove term if the whole term after tokenization and removal of stop words is a single character, or is an arabic or roman number. For this rule, the stop word list from PubMed [32] was used. This rule differs from the one in [24,26] in that it takes each token into account separately (e.g. the term "10*9/L" would be tokenised to "10 9 L" and removed by this rule since every token either is a number or a single character).

***Dosages ***[24]: the original rule addressed terms belonging to certain term types defined by the NLM in the UMLS, namely BD (Fully-specified drug brand name that can be prescribed), CD (Clinical Drug) or MS (Multiple names of branded and generic supplies or supplements). This rule was further refined by us to remove all terms that contain a dosage in percent, gram, microgram or milliliter (e.g. Oxygen 2%).

***At-sign***: this rule was implemented by us to remove terms that contain the @-character (e.g. ADHESIVE @@ BANDAGE).

***EC numbers ***[26]: Remove terms that contain enzyme classification numbers as defined by IUPAC (e.g. EC 2.7.1.112). The justification for this rule is that an EC number in the UMLS usually is mapped to a specific enzyme while it actually refers to a class of enzymes.

***Any classification ***[24]: remove terms containing the following properties: "NEC" at the end of a term and preceded by a comma, "NEC" within parentheses or brackets at the end of a term and preceded by a space, "not elsewhere classified", "unclassified", "without mention" (e.g. "Unclassified sequences").

***Any underspecification ***[24,26]: remove terms containing the following properties: "not otherwise specified", "not specified", or "unspecified"; "NOS" at the end of a term and preceded by a comma, or "NOS" within parentheses or brackets at the end of a term and preceded by a space (e.g. "Other and unspecified leukaemia").

***Miscellaneous ***[24,26]: remove terms containing the following properties: "other" at the beginning of a term and followed by a space character or at the end of a term and preceded by a space character; "deprecated", "unknown", "obsolete", "miscellaneous", or "no" at the beginning of a term and followed by a space character (e.g."Other").

***Words > 5 ***[25]: remove terms that contain more that five words (e.g. "Head and Neck Squamous Cell Carcinoma"). This rule is not applied to terms belonging to the semantic group Chemicals & Drugs.

### Term and concept recognition

For the term and concept recognition we used our concept recognition software Peregrine [33]. For this study, Peregrine was set up to mimic a minimal, general-purpose concept recognizer performing case-insensitive string lookup (ignoring punctuation), similar to, for instance, TextPresso [34]. Largest match was turned off, meaning that nested terms were counted both as a match for a longer and for a short term. Our choice of set-up was based on the fact that we clearly wanted to see the effect of the rewrite and suppression rules.

### Evaluation

Each rule was evaluated separately. To assess the effect of a rule, the difference in the set of terms identified in the corpus before and after applying the rule was determined. For rewrite rules, the number of different additional terms found was determined. In addition, for each term its frequency of occurrence in the corpus was computed. For the suppression rules, the number of different suppressed terms was determined and for each term the number of times it was suppressed in the corpus. A manual analysis of the top 50 most frequent terms and 100 randomly selected terms was performed for each rule. This analysis was used to determine the size of the effect and to judge its quality.

## Results

### Generation of new synonyms and suppression of undesired ones

The number of new terms generated by the rewrite rules and number of terms suppressed by the suppression rules are shown in Table 1. The *syntactic inversion *rule generated the highest number of new terms (231,976 terms). The number of homonyms generated for every rule is shown in Table 2. The homonyms were not used in the MEDLINE indexation. The *words > 5 *rule suppressed the highest number of terms in the thesaurus (653,128 terms). When excluding the *words > 5 *rule, a total of 257,118 undesired terms was suppressed in the UMLS, thereby decreasing its size in megabyte by 25%.

**Table 1:** New terms generated by the rewrite rules and terms suppressed by the suppression rules.

Rule	Terms in thesaurus
Original	2,696,820
***Rewrite rules***	
Syntactic inversion	231,976
Possessives	10,388
Short/long form	288
Angular brackets	2,824
Semantic type	7,231
Begin parentheses	376
End parentheses	45,265
Begin brackets	11,402
End brackets	17,620

***Suppression rules***	

Dosages	171,369
Short token	2,044
At-sign	123
EC numbers	161
Any classification	5,299
Any underspecification	40,237
Miscellaneous	37,885
Words > 5	653,128

"Terms in thesaurus" indicates the number of new terms generated by the rewrite rules and the number of terms suppressed by the suppression rules, for every rule. The row "Original" indicates the total number of terms in the thesaurus when no rewrite or suppression rule was applied.

**Table 2:** Number of homonyms (%) generated for every rewrite rule.

Rewrite rule	No of homonyms (%)
Syntactic inversion	303 (0.1)
Possessives	40 (0.4)
Short/long form	321 (52.7)
Angular brackets	218 (7.2)
Semantic type	130 (1.8)
Begin parentheses	28 (6.9)
End parentheses	5,505 (10.8)
Begin brackets	249 (2.1)
End brackets	37,083 (67.8)

The percentage is relative to the total number of rewritten terms for every rule.

### Impact on number of identified terms in the MEDLINE corpus

Of the 2,696,820 original UMLS terms, 651,268 (24.2%) were uniquely identified in the corpus, with an occurrence count of roughly 4 billion; 397,414 of the 1,294,936 distinct concepts (30.6%) were identified. The different rewrite and suppression rules had a different impact on the number of identified terms (Table 3). *Syntactic inversion *(12,433 distinct terms) had the highest impact on number of distinct terms found in the MEDLINE corpus. *Words > 5 *(5,734 distinct terms) had the highest impact on the number of distinct terms suppressed in the MEDLINE corpus. In addition, terms suppressed by the *short token *rule and the *miscellaneous *rule are found with an extremely high frequency (*short token*: roughly 2 billion times, *miscellaneous*: 91,576,083 times).

**Table 3:** Rewritten or suppressed terms and concepts found in the corpus.

Rule	Terms in corpus (all)	Terms in corpus (distinct)	Concepts in corpus (distinct)
Original	3,992,662,340	651,268	397,414

***Rewrite rules***			

Syntactic inversion	529,058	12,433	11,291
Possessives	34,211	1,134	946
Short/long form	305,541	216	182
Angular brackets	30,124	743	731
Semantic type	218,838	259	259
Begin parentheses	523	26	25
End parentheses	8,916,764	4,776	4,494
Begin brackets	176,791	274	251
End brackets	65,873	241	236

***Suppression rules***			

Dosages	109,246	5,014	4,885
Short token	1,906,901,846	1009	945
At-sign	0	0	0
EC numbers	45,138	149	146
Any classification	6,972	42	36
Any underspecification	9,470	322	290
Miscellaneous	91,576,083	1,257	1,095
Words > 5	179,051	5,734	4,665

"Terms in corpus (all)" indicates the number of occurrences of the new terms generated by the rewrite rules and the terms suppressed by the suppression rules in the corpus. "Terms in corpus (distinct)" and "Concepts in corpus (distinct)" indicate the number of unique terms and concepts produced or suppressed by the rules that were found in the corpus. The row "Original" indicates the total number of terms found in corpus when no rewrite or suppression rule was applied.

The rewrite rules also had different impact on the coverage regarding unique concepts. By rule *syntactic inversion *we have improved coverage by 2.8%, by rule *possessives *the improvement was 0.2%, by rule *short/long form *the improvement was 0.05%, by rule *angular brackets *the improvement was 0.2%, by rule *semantic type *the improvement was 0.07%, by rule *begin parentheses *the improvement was 0.006%, by rule *end parentheses *the improvement was 1.1%, by rule *begin brackets *the improvement was 0.06%, by rule *end brackets *the improvement was 0.06%, overall 5.0%

### Manual error analysis of identified terms

A sample of the 50 most frequent terms in the corpus and 100 random terms were analyzed for every rule (see additional file 1: The 50 most frequent and 100 random terms).

Based on a manual analysis of the sample terms, we found that six of the nine rewrite rules resulted in incorrectly rewritten terms: *angular brackets*, *short/long form*, *begin parentheses*, *end parentheses*, *begin brackets*, and *end brackets *(Table 4).

**Table 4:** Number of correct and incorrect terms for each of the rewrite and suppression rules.

Rule	Most frequent		Random	
***Rewrite rules***	***Correct***	***Incorrect***	***Correct***	***Incorrect***

Syntactic inversion	50	0	100	0
Possessives	50	0	100	0
Short/long form	49	1	98	2
Angular brackets	50	0	97	3
Semantic type	50	0	100	0
Begin parentheses	1	25	-	-
End parentheses	49	1	96	4
Begin brackets	38	12	91	9
End brackets	46	4	95	5

***Suppression rules***				

Dosages	50	0	100	0
Short token	50	0	100	0
At-sign	-	-	-	-
EC numbers	50	0	99	0
Any classification	50	0	100	0
Any underspecification	50	0	100	0
Miscellaneous	50	0	100	0
Words > 5	0	50	5	95

The calculations are based on the, for every rule, 50 most frequently found terms in the corpus and 100 randomly selected terms in the corpus (if available). The At-sign rule has no values because terms suppressed by this rule were not found in the corpus.

The three incorrect terms generated by the *angular brackets *rule were the terms: "<timing>C (_cum_)<meal>" rewritten as "C (_cum_)", "every <integer> weeks" rewritten as "every weeks", "every <integer> minutes" rewritten as "every minutes". Projecting the results from the random sample, the three incorrect terms would correspond to 22 terms (3% of 743 terms) found in the corpus by this rule.

The two incorrect terms generated by the *short/long form *rule in the sample were the terms "Control of skeletal myogenesis by HDAC & calcium/calmodulin-dependent kinase (CaMK)" which gave the long form "calmodulin-dependent kinase" and "Polibar Rapid (P/P)" which gave the short form "P/P". These terms do not correspond to their original UMLS terms, since the first UMLS term describes a process which is incorrectly rewritten as an enzyme, and "P/P" is not a short form of Polibar Rapid. Projecting the results from the random sample, the two incorrect terms would correspond to four terms (2% of 216 terms) found in the corpus by this rule.

Only 26 terms generated by the *begin parentheses *rule were found in the MEDLINE corpus (Table 3) and only one was correct: "(protein) methionine-R-sulfoxide reductase" rewritten as "methionine-R-sulfoxide reductase". Almost all other terms corresponded to the activity of enzymes where the application of the rule resulted in a less specific term, e.g. "(2-5')oligo(A) synthetase activity" rewritten as "oligo(A) synthetase activity". The number of terms in the random sample was equal to the total number of terms found in the corpus by this rule. There is therefore no need to project the results from the random sample.

The *end parentheses *rule had four incorrect terms in the random sample. The incorrect terms in the random sample all corresponded to loci on a chromosome, e.g. "t(3;6)(p13;q25) " rewritten as "t(3;6)". Terms generated by this rewrite rule are found with a high frequency in the corpus (Table 3), which can be explained by the fact that the removal of end parentheses can result in very general terms. For example, rewriting the term "Controls (Instrument)" results in the general term "Controls" that is found 609,492 times in the MEDLINE corpus. Projecting the results from the random sample, the four incorrect terms would correspond to 191 terms (4% of 4,776 terms) found in the corpus by this rule.

The high error rate for the rule *begin brackets *is due to the fact that many of the incorrectly rewritten terms correspond to biological activities of proteins such as the term " [pyruvate dehydrogenase (lipoamide)] phosphatase activity", which is incorrectly rewritten as "phosphatase activity". On the other hand, many of the correctly rewritten terms corresponded to terms that start with a code, e.g. " [D]Respiratory abnormalities", which is correctly rewritten as "Respiratory abnormalities". Projecting the results from the random sample, the nine incorrect terms would correspond to 25 terms (9% of 274 terms) found in the corpus by this rule.

Almost all incorrect terms produced by the *end brackets *rule corresponded to antigens of a specific bacterial strain, e.g. "Shigella flexneri 2a [II:3,4]" incorrectly rewritten as "Shigella flexneri 2a". Terms generated by this rewrite rule are found with a high frequency in the corpus (Table 3), which can be explained by the fact that the removal of end brackets from terms such as "Abstracts [Publication Type]" results in the very general term "Abstracts", which is found 25,082 times in the MEDLINE corpus. Projecting the results from the random sample, the five incorrect terms would correspond to 12 terms (5% of 241 terms) found in the corpus by this rule.

Most terms suppressed by the *words > 5 *rule were found to be valuable terms that did not need to be suppressed, e.g. "Carcinoma of the Head and Neck", "insulin-like growth factor binding protein 1". Projecting the results from the random sample, the 95 incorrect terms would correspond to 4,432 terms (95% of 4,665 terms) found in the corpus by this rule.

None of the suppression rules except the *words > 5 *rule caused any correct term to be suppressed in the sample (Table 4). All suppressed terms were either too generic (e.g. "Unspecified conditions", "Of"), highly unlikely to be found in the literature (e.g. "Symptoms, signs and abnormal clinical and laboratory findings, not elsewhere classified"), or suppressed for specific needs (terms from the *dosages *and *EC numbers *rules).

## Discussion

To make the UMLS more suitable for biomedical text mining we implemented and evaluated nine term rewrite and eight term suppression rules. In the creation of these rules, we used and refined many of the UMLS string properties identified by McCray et al. [24,25] and Rogers and Aronson [26], together with an additional set of new properties discovered by our group during our work with the UMLS. Our work complements the work by McCray et al. and Rogers and Aronson in that we measured the impact on the number of terms identified by the different rules on all of MEDLINE (1965-2007), whereas the others only reported the number of strings in the UMLS that were affected by the specific string properties, and in that we also performed a manual analysis of the rewritten terms retrieved from the corpus and of the terms that were suppressed in the corpus. This was done in order to establish that the rewritten terms indeed correspond to the original terms and that only undesired terms were suppressed by the suppression rules.

The goal set for the rewrite rules was to increase the number of synonyms for a UMLS concept and thereby also increase the number of times a concept will be correctly identified in text, thus increasing the recall of biomedical term identification. Good rewrite rules should not generate terms that do not correspond to the original term. This holds true for the rules *syntactic inversion*, *possessives *and *semantic type*. The *angular brackets *rule and the *short/long form *rule generated a few incorrect cases. The incorrect cases from the *angular brackets *rule represent repeat patterns used in coding meal-related timings in patient records. These terms are in fact compositional grammar representing a class of "terms" in which various parts of a complex term are separated to their primitive codes and then put together through, for example, qualifiers. Hypothetically, such template terms could yield instance terms that have matches in the text. Rewriting these terms alters the template pattern and therefore the meaning of the term. Despite the incorrect cases generated by the *angular brackets *rule we recommend it to be used, but with a manual check of the results. We argue that this is feasible considering the small number of incorrect cases. We also recommend the use of the *short/long form *rule together with a manual check of the results. We find this advisable since the number of terms generated by the rule is relatively small (288 terms) but significant: it for example adds the commonly used abbreviation "SSRIs" to the term "Selective Serotonin Reuptake Inhibitors". It can also be noted that about half of the terms generated by this rule were homonyms. This indicates that the rule gives rise to quite ambiguous terms, which is another reason why we recommend a manual check of the results of this rule. Our analysis revealed that even though the different rules for rewriting terms with parentheses or brackets had an impact on the number of rewritten terms found, the quality of the rewritten terms was not perfect. The terms giving most problems were the names of biological entities, such as a genetic locus or the activity of an enzyme. These problems might be solved by introducing the criteria that parentheticals at the beginning (end) of a term should only be removed if they are followed (preceded) by a white space. This however would cause the rules to miss obvious cases without a white space where rewriting is necessary, such as " [M]Lymphoid leukaemias" (where the [M] is specific for the Read Codes vocabulary). A more promising way to tackle this problem is to analyze what kind of strings are found between parentheticals in the UMLS and based on these findings try to rewrite the terms. Our group has in this manner found that in 7,231 cases in the UMLS, the string between parentheses at the end of a term actually corresponds to the semantic type to which the term belongs. The implementation of this *semantic type *rule caused 259 new distinct terms to be found 218,838 times in our corpus and thus improves the recall of the UMLS-based term matching. Using the rewrite rules that passed our evaluation (rules 1-5) we were able to identify 1,117,772 new occurrences of 14,784 rewritten terms in the corpus. Projecting the results from the manual evaluation of 100 random rewritten terms per rule, 26 of these 14,784 terms would be incorrect.

Removal of erroneous synonyms improves the precision of the UMLS terms and removal of unnecessary terms and synonyms reduces the size of the UMLS, thus improving its efficiency. The evaluation of the suppression rules showed that all except one, namely the *words > 5 *rule, are safe to apply when the UMLS is to be used for concept identification in text.

### Use scenarios

The following use scenarios illustrate the usefulness of the rules:

#### 1) Concept-based biomedical information extraction

The rules that passed the evaluation in this work can be used to prepare the UMLS for use in a lexicon-based information extraction pipeline with the goal of identifying biomedical concepts in text. For illustration, we used the rules to prepare the UMLS for the indexing of MEDLINE abstracts with MetaMap. It is worth mentioning that most of the rules are already partly or fully implemented in MetaMap and that it is not possible to measure the exact effect of the different rules on MetaMap since MetaMap also performs a number of steps (part-of-speech tagging, shallow parsing and normalization) that all affect its performance. MetaMap also has an internal rule engine that cannot be switched on or off for specific rules. Indeed, by applying the rules to the UMLS and subsequently indexing a random set of 10,000 MEDLINE abstracts using MetaMap, only a minor increase in recall and precision was gained (31 additional concepts were recognized and 95 concepts were suppressed, all manually checked and found to be correctly recognized or suppressed). It is worth noting that MetaMap is not designed to work on large corpora: indexing the 10,000 abstracts took 33 hours on a medium performance computer. Using Peregrine with the settings described in this paper, 17,674,805 citations (9,446,335 of these have an abstract) were indexed within the same amount of time.

#### 2) Chemical name identification

In a separate study, we used the rules suitable for chemical terms as a pre-processing step in the creation of a multi-source chemical dictionary [35]. We used the suppression rules *short token*, *dosages*, *at-sign*, *any underspecification*, and *miscellaneous*, and the rewrite rules *syntactic inversion*, *possessives*, and *short form/long form *for this purpose. The dictionary was tested on a corpus annotated with chemical entities [36] and recall and precision was calculated. The rules doubled the precision, leaving the recall practically unchanged. From this use case it is obvious that the suppression rules played a large role in increasing the precision of a chemical dictionary by removing highly ambiguous terms that are rarely used as synonyms for chemicals in text. Examples of such synonyms are single letter acronyms and general English words. The rewrite rules played a less important role, only generating a few extra hits that did not influence the recall much.

### Limitations

A limitation for the generalizability of our study is that we restricted ourselves to MEDLINE and did not include other types of text such as electronic patient records, which have a different structure that might influence the performance of the rules.

### Future work

A restriction on size or on the type of content within parentheticals might lead to additional useful rewrite or suppression rules. Furthermore, a vocabulary-based suppression of terms in the UMLS might also be applicable since each vocabulary has been independently developed and adheres to its own rules. One could for example question the use of the vocabulary NCI modified Common Terminology Criteria for Adverse Events v3.0, 2003 (NCI-CTCAE), for which only two out of the 4504 terms in the vocabulary were found in the corpus. A quick analysis of the terms in NCI-CTCAE showed that many of them may be useful for clinical applications but not for knowledge discovery aiming at for example finding links between chemicals and adverse events in free text. An example is the term "CTCAE Grade 1 Supraventricular extrasystoles (Premature Atrial Contractions; Premature Nodal/Junctional Contractions)", which is very specific but will not be found in free text. Another example comes from Read codes where an axis indicator as [M] is often used before a term.

To further investigate the generalizability of the rules they should be tested on another type of text than MEDLINE, for example electronic patient records.

## Conclusions

We recommend the usage of the five rewrite rules and seven suppression rules that passed our evaluation when the UMLS is to be used for term identification in free text. Using these five rewrite rules we were able to identify 1,117,772 new occurrences of 14,784 rewritten terms in MEDLINE. Without the rewriting, we recognized 651,268 terms belonging to 397,414 concepts; with rewriting, we recognized 666,053 terms belonging to 410,823 concepts, which is an increase of 2.8% in the number of terms and an increase of 3.4% in the number of concepts recognized. Using the seven suppression rules, a total of 257,118 undesired terms were suppressed in the UMLS, thereby decreasing its size in megabyte by 25%, and 7,397 terms were suppressed in the corpus. By rewriting and suppressing the UMLS (and thereby increasing its recall and precision) it becomes more suitable for biomedical text mining purposes, such as information retrieval and knowledge discovery.

All the rules evaluated in this paper can be applied to UMLS data by using the software program Casper, which is available online at http://www.biosemantics.org. Casper takes a UMLS data file as input and gives a rewritten and suppressed UMLS data file as output. This UMLS data file can then be used together with any concept recognition software of choice. Please note that Casper operates on UMLS data, for which a license is needed.

## Competing interests

The authors declare that they have no competing interests.

## Authors' contributions

KMH participated in the design of the study, implemented the rules, performed the analysis and drafted the manuscript. EMM conceived of the study, participated in its design and coordination, generated the analysis data and helped to draft the manuscript. MJS participated in the design of the study, performed the MEDLINE indexations and helped to draft the manuscript. BJAS participated in the design of the study and helped to draft the manuscript. JAK participated in the design and coordination of the study and helped to draft the manuscript. All authors read and approved the final manuscript.

## Supplementary Material

Additional file 1 The 50 most frequent and 100 random terms. Click here for file
