# Supplementary material for: Rewriting and suppressing UMLS terms for improved biomedical term identification
Source: J Biomed Semantics. 2010 Mar 31;1:5. doi: 10.1186/2041-1480-1-5 (PMC2895736; doi:10.1186/2041-1480-1-5)
Supplement: Additional file 1 — The 50 most frequent and 100 random terms. [file 2041-1480-1-5-S1.PDF]

Supplement 1. Terms in bold are considered incorrect.

REWRITE RULES

Rule|CUI|Original term|Rewritten term|Frequency

TOP 50 FREQUENT Syntactic inv.:
Syntactic inv.|1578672|Process, The|The Process|93815
Syntactic inv.|20538|Hypertension, arterial|arterial Hypertension|24455
Syntactic inv.|1258983|Review, Systematic|Systematic Review|15321
Syntactic inv.|449851|Methods, Other|Other Methods|12136
Syntactic inv.|27051|Infarction (MI), myocardial|myocardial Infarction (MI)|7898
Syntactic inv.|238605|AFRICAN, SOUTH|SOUTH AFRICAN|7564
Syntactic inv.|1675|Human, Adult|Adult Human|7480
Syntactic inv.|181075|Grafts, Bone|Bone Grafts|5752
Syntactic inv.|1550269|Jugular, Internal|Internal Jugular|5548
Syntactic inv.|25123|Medicine, Chinese|Chinese Medicine|5275
Syntactic inv.|745442|Catheters, Venous|Venous Catheters|4625
Syntactic inv.|717385|Albumin, Human|Human Albumin|3086
Syntactic inv.|1140606|SORTER, CELL|CELL SORTER|3008
Syntactic inv.|21375|Inflammation, allergic|allergic Inflammation|2954
Syntactic inv.|238013|ASPERGILLOSIS, INVASIVE|INVASIVE ASPERGILLOSIS|2835
Syntactic inv.|1554888|Injection, intravitreal|intravitreal Injection|2564
Syntactic inv.|23089|Radiation, Laser|Laser Radiation|2543
Syntactic inv.|369241|Fungi, Filamentous|Filamentous Fungi|2505
Syntactic inv.|238644|ANEMIA, SEVERE|SEVERE ANEMIA|2477
Syntactic inv.|686377|involvement, CNS|CNS involvement|2297
Syntactic inv.|1510991|Autoradiography, Quantitative|Quantitative Autoradiography|2234
Syntactic inv.|1546658|Tube, Gastric|Gastric Tube|2187
Syntactic inv.|341950|Preeclampsia, severe|severe Preeclampsia|2155
Syntactic inv.|32428|Polyacrylamide Gel Electrophoresis, Two-Dimensional|Two-Dimensional Polyacrylamide Gel Electrophoresis|2144
Syntactic inv.|238606|AFRICAN, WEST|WEST AFRICAN|2092
Syntactic inv.|1546605|Drainage, Tube|Tube Drainage|2083
Syntactic inv.|73385|Ringer's, lactated|lactated Ringer's|2040
Syntactic inv.|152021|Heart Diseases, Congenital|Congenital Heart Diseases|1961
Syntactic inv.|1555389|Instillation, intratracheal|intratracheal Instillation|1898
Syntactic inv.|1519445|Spectroscopy, Circular Dichroism|Circular Dichroism Spectroscopy|1884
Syntactic inv.|687568|STENT, CORONARY|CORONARY STENT|1882
Syntactic inv.|717972|Pertussis, acellular|acellular Pertussis|1872
Syntactic inv.|487019|Interleukin 2 receptor, soluble|soluble Interleukin 2 receptor|1743
Syntactic inv.|1519521|Tissue, Fresh|Fresh Tissue|1687
Syntactic inv.|41618|Diagnosis, Ultrasound|Ultrasound Diagnosis|1659
Syntactic inv.|1509244|Epidermal Growth Factor, Human|Human Epidermal Growth Factor|1600
Syntactic inv.|553580|Sarcoma, Ewing|Ewing Sarcoma|1512
Syntactic inv.|1448177|tumor necrosis factor, human|human tumor necrosis factor|1462
Syntactic inv.|11463|Partial dentures, Removable|Removable Partial dentures|1456
Syntactic inv.|34991|Rehabilitation, Medical|Medical Rehabilitation|1449
Syntactic inv.|239998|INFECTION, RECURRENT|RECURRENT INFECTION|1375
Syntactic inv.|25326|Gonadotropin, menopausal|menopausal Gonadotropin|1304
Syntactic inv.|59291|endotoxin, Escherichia coli|Escherichia coli endotoxin|1298
Syntactic inv.|1560462|Incontinence, anal|anal Incontinence|1298
Syntactic inv.|1553218|Implantation, subcutaneous|subcutaneous Implantation|1291
Syntactic inv.|238603|AFRICAN, EAST|EAST AFRICAN|1252
Syntactic inv.|1519104|Markers, Risk|Risk Markers|1247
Syntactic inv.|21469|Inhibition, NOS|NOS Inhibition|1228

Syntactic inv.|282459|Clinical Trial, Phase I|Phase I Clinical Trial|1201
Syntactic inv.|153257|FEVER, VALLEY|VALLEY FEVER|1196
END TOP 50 FREQUENT

RANDOM CASES Syntactic inv.:
Syntactic inv.|279936|therapy, TNF|TNF therapy|457
Syntactic inv.|336569|FORK, TUNING|TUNING FORK|455
Syntactic inv.|242079|RHEUMATISM, INFLAMMATORY|INFLAMMATORY RHEUMATISM|375
Syntactic inv.|1456575|Leukemia, Adult Acute|Adult Acute Leukemia|327
Syntactic inv.|12164|Diets, Formula|Formula Diets|314
Syntactic inv.|1546737|Site, Pin|Pin Site|247
Syntactic inv.|179591|CUFF, NERVE|NERVE CUFF|201
Syntactic inv.|13842|Electron microscopy, diagnostic|diagnostic Electron microscopy|79
Syntactic inv.|341950|Pre-eclamptic, severe|severe Pre-eclamptic|77
Syntactic inv.|183744|Sutures, PTFE|PTFE Sutures|52
Syntactic inv.|30486|EXTREMITY PARALYSIS, LOWER|LOWER EXTREMITY PARALYSIS|40
Syntactic inv.|854215|bacterial; meningitis, gram-negative|gram-negative bacterial; meningitis|38
Syntactic inv.|1292068|Antibody, IgA class|IgA class Antibody|33
Syntactic inv.|452168|Hypospadias, glandular|glandular Hypospadias|31
Syntactic inv.|1456394|retinoid X receptor, gamma|gamma retinoid X receptor|27
Syntactic inv.|62113|female protein, hamster|hamster female protein|25
Syntactic inv.|238551|Abdominal pain, left lower quadrant|left lower quadrant Abdominal pain|24
Syntactic inv.|492618|GAUGE, MEASURING|MEASURING GAUGE|23
Syntactic inv.|1392622|motor; tic, chronic|chronic motor; tic|17
Syntactic inv.|183943|TIMER, COAGULATION|COAGULATION TIMER|15
Syntactic inv.|296376|DNA polymerase, Thermus thermophilus|Thermus thermophilus DNA polymerase|13
Syntactic inv.|1392261|cerebral; injury, diffuse|diffuse cerebral; injury|13
Syntactic inv.|397978|Bypass, iliac-femoral|iliac-femoral Bypass|11
Syntactic inv.|493786|Allografts, Cardiac Valve|Cardiac Valve Allografts|11
Syntactic inv.|383080|large surface protein, hepatitis B virus|hepatitis B virus large surface protein|10
Syntactic inv.|302682|Eyelid Weights, External|External Eyelid Weights|9
Syntactic inv.|182242|Phantoms, Ultrasound|Ultrasound Phantoms|8
Syntactic inv.|184093|Trocars, Other|Other Trocars|8
Syntactic inv.|269111|Endometriosis, cul-de-sac|cul-de-sac Endometriosis|7
Syntactic inv.|343900|HISTOPLASMOSIS, CHRONIC CAVITARY|CHRONIC CAVITARY HISTOPLASMOSIS|7
Syntactic inv.|202750|Orbitography, positive contrast|positive contrast Orbitography|6
Syntactic inv.|338575|THROMBOSIS, SUPERIOR LONGITUDINAL SINUS|SUPERIOR LONGITUDINAL SINUS THROMBOSIS|6
Syntactic inv.|525913|circumsporozoite (CS) protein, Plasmodium|Plasmodium circumsporozoite (CS) protein|6
Syntactic inv.|155681|acute; pericarditis, nonspecific|nonspecific acute; pericarditis|5
Syntactic inv.|175702|HYPERCALCEMIA, INFANTILE IDIOPATHIC|INFANTILE IDIOPATHIC HYPERCALCEMIA|5
Syntactic inv.|230923|Cell wall, prokaryotic|prokaryotic Cell wall|5
Syntactic inv.|752227|Camomile, Roman|Roman Camomile|5
Syntactic inv.|1568263|bone morphogenetic protein-4, Xenopus|Xenopus bone morphogenetic protein-4|5
Syntactic inv.|1306|acute; hepatitis, alcoholic|alcoholic acute; hepatitis|4
Syntactic inv.|278587|cervix cancer, IA|IA cervix cancer|4
Syntactic inv.|247213|alphaPS2 integrin, Drosophila|Drosophila alphaPS2 integrin|3
Syntactic inv.|625082|Pyridinium, 1-butyl-|1-butyl-Pyridinium|3
Syntactic inv.|1261109|cerebellar; ataxia, alcoholic|alcoholic cerebellar; ataxia|3
Syntactic inv.|1565051|disabled 2 interacting protein 2, mouse|mouse disabled 2 interacting protein 2|3
Syntactic inv.|62156|Hsf protein, Drosophila|Drosophila Hsf protein|2
Syntactic inv.|73263|ribosomal large subunit protein L7a, human|human ribosomal large subunit protein L7a|2
Syntactic inv.|82736|glycoprotein B, infectious laryngotracheitis virus|infectious laryngotracheitis virus glycoprotein B|2
Syntactic inv.|84264|Q protein, Bacteriophage lambda|Bacteriophage lambda Q protein|2

Syntactic inv.|168141|protein p12, African swine fever virus|African swine fever virus protein p12|2
Syntactic inv.|216698|cysteine-rich protein, avian|avian cysteine-rich protein|2
Syntactic inv.|235698|Abdominal distension, gaseous|gaseous Abdominal distension|2
Syntactic inv.|278166|Glasgow coma scale, 5|5 Glasgow coma scale|2
Syntactic inv.|342625|Hemosiderosis, acquired|acquired Hemosiderosis|2
Syntactic inv.|383422|fusion protein, PPRV|PPRV fusion protein|2
Syntactic inv.|757449|defensin, ant|ant defensin|2
Syntactic inv.|1138292|esterase 6, Drosophila|Drosophila esterase 6|2
Syntactic inv.|1266079|Mucinous adenocarcinoma, endocervical type|endocervical type Mucinous adenocarcinoma|2
Syntactic inv.|1433172|aldehyde dehydrogenase 3A1, human|human aldehyde dehydrogenase 3A1|2
Syntactic inv.|1453309|MCM10 protein, human|human MCM10 protein|2
Syntactic inv.|1505114|Rad51 protein, Drosophila|Drosophila Rad51 protein|2
Syntactic inv.|1565803|Kgf protein, mouse|mouse Kgf protein|2
Syntactic inv.|610|3-Pyridinecarboxamide, 6-amino-|6-amino-3-Pyridinecarboxamide|1
Syntactic inv.|17245|Pentanoic acid, 5-(2,5-dimethylphenoxy)-2,2-dimethyl-|5-(2,5-dimethylphenoxy)-2,2-dimethyl-Pentanoic acid|1
Syntactic inv.|44642|5,8,14-Eicosatrienoic acid, 10,11,12-trihydroxy-|10,11,12-trihydroxy-5,8,14-Eicosatrienoic acid|1
Syntactic inv.|62366|Hb, beta-99 Asp-Asn|beta-99 Asp-Asn Hb|1
Syntactic inv.|68804|Assimilatory Nitrate Reductase, NADPH-Dependent|NADPH-Dependent Assimilatory Nitrate Reductase|1
Syntactic inv.|81820|BCM1 antigen, human|human BCM1 antigen|1
Syntactic inv.|84690|G surface protein, Paramecium primaurelia|Paramecium primaurelia G surface protein|1
Syntactic inv.|179641|Carts, Housekeeping|Housekeeping Carts|1
Syntactic inv.|180414|Dialyzers, High-Permeability|High-Permeability Dialyzers|1
Syntactic inv.|180705|Elevators, Uterine|Uterine Elevators|1
Syntactic inv.|182495|Prostheses, Muscle|Muscle Prostheses|1
Syntactic inv.|184206|DISPENSER, LIQUID MEDICATION|LIQUID MEDICATION DISPENSER|1
Syntactic inv.|251930|POPA protein, Ralstonia solanacearum|Ralstonia solanacearum POPA protein|1
Syntactic inv.|432098|Cleft soft palate, central|central Cleft soft palate|1
Syntactic inv.|441072|SPECTACLE, MAGNIFYING|MAGNIFYING SPECTACLE|1
Syntactic inv.|443370|1-BP, propane|propane 1-BP|1
Syntactic inv.|527101|cleavage signal-1 protein, human|human cleavage signal-1 protein|1
Syntactic inv.|758157|glycoprotein 5, PRRSV|PRRSV glycoprotein 5|1
Syntactic inv.|855261|Delusional disorder, erotomanic type|erotomanic type Delusional disorder|1
Syntactic inv.|914891|a protein, Drosophila|Drosophila a protein|1
Syntactic inv.|960461|retinal proteoglycan IPM 150, human|human retinal proteoglycan IPM 150|1
Syntactic inv.|966976|CD4 silencer, AGM|AGM CD4 silencer|1
Syntactic inv.|967718|RAP1A protein, human|human RAP1A protein|1
Syntactic inv.|1136749|BORIS protein, human|human BORIS protein|1
Syntactic inv.|1137951|deltaNp73, human|human deltaNp73|1
Syntactic inv.|1257651|histone hairpin-binding protein, C elegans|C elegans histone hairpin-binding protein|1
Syntactic inv.|1309561|NPC1L1 protein, human|human NPC1L1 protein|1
Syntactic inv.|1447758|DNMT3L protein, human|human DNMT3L protein|1
Syntactic inv.|1448088|hyaluronan binding protein 2, human|human hyaluronan binding protein 2|1
Syntactic inv.|1448610|poly(A)polymerase I, E coli|E coli poly(A)polymerase I|1
Syntactic inv.|1448995|Dos protein, Drosophila|Drosophila Dos protein|1
Syntactic inv.|1450557|MAC25 protein, human|human MAC25 protein|1
Syntactic inv.|1454432|ecdysoneless protein, Drosophila|Drosophila ecdysoneless protein|1
Syntactic inv.|1513865|NZR, Rat Strain|Rat Strain NZR|1
Syntactic inv.|1565364|glutathione S-transferase A3-3, human|human glutathione S-transferase A3-3|1
Syntactic inv.|1566084|SigV protein, Enterococcus faecalis|Enterococcus faecalis SigV protein|1
Syntactic inv.|1611038|archaemetzincin-1, human|human archaemetzincin-1|1
Syntactic inv.|1620268|ribosomal protein S5, mouse|mouse ribosomal protein S5|1
Syntactic inv.|1621263|LcrV protein, Yersinia|Yersinia LcrV protein|1
END RANDOM CASES

TOP 50 FREQUENT Possessives:
Possessives|73386|Ringer's solution|Ringer solution|3065
Possessives|440466|Hen's egg|Hen egg|2673
Possessives|1708064|Fisher's Exact Test|Fisher Exact Test|1872
Possessives|1527336|Sjögren's syndrome|Sjögren syndrome|1597
Possessives|553580|Ewing's sarcoma|Ewing sarcoma|1512
Possessives|36920|Sézary's syndrome|Sézary syndrome|1414
Possessives|77548|Tyrode's solution|Tyrode solution|1157
Possessives|454792|King's County|King County|582
Possessives|242543|Women's Group|Women Group|580
Possessives|7125|Ehrlich's tumor|Ehrlich tumor|495
Possessives|31900|Robin's syndrome|Robin syndrome|490
Possessives|42170|Harada's syndrome|Harada syndrome|425
Possessives|15625|Fanconi's anaemia|Fanconi anaemia|367
Possessives|73385|Ringer's lactate|Ringer lactate|353
Possessives|27831|Recklinghausen's neurofibromatosis|Recklinghausen neurofibromatosis|333
Possessives|264092|Burn's disease|Burn disease|327
Possessives|79731|B-Cell Non-Hodgkin's Lymphoma|B-Cell Non-Hodgkin Lymphoma|302
Possessives|32463|Osler's disease|Osler disease|271
Possessives|39445|Osler's disease|Osler disease|271
Possessives|1708064|Fisher's Test|Fisher Test|261
Possessives|30567|Parkinson's syndrome|Parkinson syndrome|252
Possessives|270868|Bonnet's syndrome|Bonnet syndrome|252
Possessives|400084|Hartmann's procedure|Hartmann procedure|251
Possessives|742|Abelson's virus|Abelson virus|240
Possessives|271093|Stargardt's disease|Stargardt disease|234
Possessives|15458|Romberg's syndrome|Romberg syndrome|233
Possessives|65143|Locke's solution|Locke solution|210
Possessives|16712|Complete Freund's Adjuvant|Complete Freund Adjuvant|194
Possessives|6413|Burkitt's lymphomas|Burkitt lymphomas|187
Possessives|20017|Children's hospital|Children hospital|180
Possessives|270615|Nielsen's syndrome|Nielsen syndrome|170
Possessives|26926|Koch's bacillus|Koch bacillus|163
Possessives|73385|Lactated Ringer's Solution|Lactated Ringer Solution|156
Possessives|301528|Lactated Ringer's solution|Lactated Ringer solution|156
Possessives|406557|Kindler's syndrome|Kindler syndrome|148
Possessives|272199|Omenn's syndrome|Omenn syndrome|146
Possessives|266815|Cow's milk allergy|Cow milk allergy|145
Possessives|339510|Best's disease|Best disease|144
Possessives|580779|Russell's viper venom|Russell viper venom|139
Possessives|440448|Cow's milk protein|Cow milk protein|130
Possessives|224724|Lisfranc's joint|Lisfranc joint|126
Possessives|85277|Munchausen's by proxy|Munchausen by proxy|117
Possessives|1334815|multicentric Castleman's disease|multicentric Castleman disease|116
Possessives|175756|Blount's disease|Blount disease|112
Possessives|700365|Herring's bodies|Herring bodies|112
Possessives|221043|Liddle's syndrome|Liddle syndrome|111
Possessives|543710|Rokitansky's syndrome|Rokitansky syndrome|109
Possessives|227426|Ball's valves|Ball valves|104
Possessives|596979|Muller's cell|Muller cell|104
Possessives|730313|Terson's syndrome|Terson syndrome|104
END TOP 50 FREQUENT

RANDOM CASES Possessives:
Possessives|1527336|Sjögren's syndrome|Sjögren syndrome|1597
Possessives|15625|Fanconi's anaemia|Fanconi anaemia|367

Possessives|742|Abelson's virus|Abelson virus|240
Possessives|15458|Romberg's syndrome|Romberg syndrome|233
Possessives|580779|Russell's viper venom|Russell viper venom|139
Possessives|229865|Waldeyer's ring|Waldeyer ring|49
Possessives|459892|Waldeyer's ring|Waldeyer ring|49
Possessives|277977|Murphy's sign|Murphy sign|37
Possessives|278586|Metastatic Ewing's Sarcoma|Metastatic Ewing Sarcoma|34
Possessives|553580|Ewing's tumour|Ewing tumour|34
Possessives|32290|Mendelson's syndrome|Mendelson syndrome|30
Possessives|680964|consumer's surplus|consumer surplus|28
Possessives|220644|pediatric Hodgkin's disease|pediatric Hodgkin disease|26
Possessives|277811|Fowler's position|Fowler position|26
Possessives|1429|Warthin's tumour|Warthin tumour|25
Possessives|278206|Queckenstedt's test|Queckenstedt test|25
Possessives|432365|Lenz's syndrome|Lenz syndrome|23
Possessives|817105|Calot's triangle|Calot triangle|23
Possessives|995830|Whipple's bacillus|Whipple bacillus|23
Possessives|156147|CROHN'S DISEASE OF THE COLON|CROHN DISEASE OF THE COLON|22
Possessives|1279809|Maintaining patient's safety|Maintaining patient safety|20
Possessives|85400|Alzheimer's neurofibrillary degeneration|Alzheimer neurofibrillary degeneration|18
Possessives|272464|Le Fort's fracture|Le Fort fracture|18
Possessives|152094|Nezelof's syndrome|Nezelof syndrome|17
Possessives|339085|Ascher's syndrome|Ascher syndrome|17
Possessives|2994|QUINCKE'S OEDEMA|QUINCKE OEDEMA|16
Possessives|1512027|Doctor's Degree|Doctor Degree|16
Possessives|1282916|Secondary Raynaud's phenomenon|Secondary Raynaud phenomenon|15
Possessives|24305|Nonhodgkin's Lymphoma|Nonhodgkin Lymphoma|12
Possessives|814818|children's literature|children literature|12
Possessives|1555596|Provider's Office|Provider Office|12
Possessives|220644|Pediatric Hodgkin's Lymphoma|Pediatric Hodgkin Lymphoma|11
Possessives|225297|Alcock's canal|Alcock canal|11
Possessives|752347|Lewy body variant of Alzheimer's disease|Lewy body variant of Alzheimer disease|11
Possessives|666151|Mosher's acid|Mosher acid|9
Possessives|1512431|High Grade B-Cell Non-Hodgkin's Lymphoma|High Grade B-Cell Non-Hodgkin Lymphoma|9
Possessives|27961|Ota's naevus|Ota naevus|7
Possessives|152268|Hodgkin's disease, nodular sclerosis|Hodgkin disease, nodular sclerosis|7
Possessives|205748|Clark's Nevus|Clark Nevus|7
Possessives|341253|Brunner's gland hyperplasia|Brunner gland hyperplasia|7
Possessives|1336077|Non-endemic Burkitt's lymphoma|Non-endemic Burkitt lymphoma|7
Possessives|4766|Bartholin's abscess|Bartholin abscess|6
Possessives|1186762|Dakin's solution|Dakin solution|6
Possessives|43213|Women's Rights|Women Rights|5
Possessives|279980|Extraosseous Ewing's Tumor|Extraosseous Ewing Tumor|5
Possessives|327113|Reeves's turtle|Reeves turtle|5
Possessives|343280|Haglund's disease|Haglund disease|5
Possessives|738214|Camper's fascia|Camper fascia|5
Possessives|36025|Brewer's yeast|Brewer yeast|4
Possessives|325299|Thomson's gazelle|Thomson gazelle|4
Possessives|519030|Friedlander's pneumonia|Friedlander pneumonia|4
Possessives|1333800|Primary Gastrointestinal Non-Hodgkin's Lymphoma|Primary Gastrointestinal Non-Hodgkin Lymphoma|4
Possessives|1335365|Non-Hodgkin's Lymphoma of the Parotid Gland|Non-Hodgkin Lymphoma of the Parotid Gland|4
Possessives|1532963|Women's lacrosse|Women lacrosse|4
Possessives|4789|Rosenthal's vein|Rosenthal vein|3
Possessives|21345|Pfeiffer's disease|Pfeiffer disease|3
Possessives|103301|Angeli's salt|Angeli salt|3

Possessives|153791|Hodgkin's Lymphoma of the Spleen|Hodgkin Lymphoma of the Spleen|3
Possessives|270887|Vernet's syndrome|Vernet syndrome|3
Possessives|270898|Klumpke's palsy|Klumpke palsy|3
Possessives|677951|Stage I Hodgkin's Disease|Stage I Hodgkin Disease|3
Possessives|1096550|Capdepont's disease|Capdepont disease|3
Possessives|1336755|Non-Hodgkin's Lymphoma of the Thyroid Gland|Non-Hodgkin Lymphoma of the Thyroid Gland|3
Possessives|1626422|Number of positive Ranson's criteria|Number of positive Ranson criteria|3
Possessives|43211|WOMEN'S HEALTH EDUCATION|WOMEN HEALTH EDUCATION|2
Possessives|153564|Kaposi's Sarcoma of the Lung|Kaposi Sarcoma of the Lung|2
Possessives|158248|Baastrup's syndrome|Baastrup syndrome|2
Possessives|227547|Heister's valve|Heister valve|2
Possessives|229127|Reichert's membrane|Reichert membrane|2
Possessives|231669|Finkelstein's test|Finkelstein test|2
Possessives|236058|Descemet's detachment|Descemet detachment|2
Possessives|278586|Ewing's sarcoma, metastatic|Ewing sarcoma, metastatic|2
Possessives|279980|Extra-osseous Ewing's sarcoma|Extra-osseous Ewing sarcoma|2
Possessives|4420|Marek's disease herpesvirus|Marek disease herpesvirus|1
Possessives|5592|fancier's disease|fancier disease|1
Possessives|8441|Codman's tumor|Codman tumor|1
Possessives|11989|Engelman's disease|Engelman disease|1
Possessives|19829|Hodgkin's disease/lymphoma|Hodgkin disease/lymphoma|1
Possessives|152084|Jaccoud's syndrome|Jaccoud syndrome|1
Possessives|153792|Hodgkin's disease stage III|Hodgkin disease stage III|1
Possessives|153792|Stage III Hodgkin's Disease|Stage III Hodgkin Disease|1
Possessives|162809|de Morsier-Kallman's syndrome|de Morsier-Kallman syndrome|1
Possessives|195734|Scanzoni's maneuver|Scanzoni maneuver|1
Possessives|225314|Buck's fascia|Buck fascia|1
Possessives|228360|Forel's field H2|Forel field H2|1
Possessives|231790|Thompson's squeeze test|Thompson squeeze test|1
Possessives|263957|Tailor's bunion|Tailor bunion|1
Possessives|264099|Freiberg's infraction|Freiberg infraction|1
Possessives|271488|Active Meniere's disease|Active Meniere disease|1
Possessives|278590|Relapsed Ewing's Sarcoma|Relapsed Ewing Sarcoma|1
Possessives|278879|Childhood Burkitt's Lymphoma|Childhood Burkitt Lymphoma|1
Possessives|341700|Bartter's syndrome with hypercalciuria and nephrocalcinosis|Bartter syndrome with hypercalciuria and nephrocalcinosis|1
Possessives|430480|Buerger's test|Buerger test|1
Possessives|438114|Patient's condition unstable|Patient condition unstable|1
Possessives|442232|Stenver's projection|Stenver projection|1
Possessives|457147|Bachman's bundle|Bachman bundle|1
Possessives|677954|Hodgkin's Lymphoma Stage IV|Hodgkin Lymphoma Stage IV|1
Possessives|796548|metastatic Ewing's sarcoma/primitive neuroectodermal tumor|metastatic Ewing sarcoma/primitive neuroectodermal tumor|1
Possessives|1334149|Iatrogenic Kaposi's Sarcoma|Iatrogenic Kaposi Sarcoma|1
Possessives|1704323|Nipple Paget's Disease|Nipple Paget Disease|1
END RANDOM CASES

TOP 50 FREQUENT Short/long f.:
Short/long f.|917713|Becker muscular dystrophy (BMD)|BMD|50460
Short/long f.|11185|Dehydroepiandrosterone (DHA)|DHA|17734
Short/long f.|813025|Knee Orthosis (KO)|KO|14709
Short/long f.|1725182|Polibar Rapid (P/P)|P/P|13594
Short/long f.|85280|Alagille syndrome (AGS)|AGS|7722
Short/long f.|1561644|Chronic kidney disease (CKD)|CKD|7092
Short/long f.|175693|Silver-Russell syndrome (SRS)|SRS|6679

Short/long f.|796160|Snyder-Robinson syndrome (SRS)|SRS|6679
Short/long f.|246450|M protein, Mouse hepatitis virus (MHV)|MHV|6203
Short/long f.|360105|Selective Serotonin Reuptake Inhibitors (SSRIs)|SSRIs|5960
Short/long f.|795864|Smith-Magenis syndrome (SMS)|SMS|5546
Short/long f.|1456025|Intravascular ultrasound (IVUS)|Intravascular ultrasound|5417
Short/long f.|182281|Picture Archiving and Communication Systems (PACS)|PACS|5390
Short/long f.|1456025|Intravascular ultrasound (IVUS)|IVUS|5377
Short/long f.|65088|Lithium chloride (LiCl)|LiCl|5295
Short/long f.|796136|Richards-Rundle syndrome (RRS)|RRS|5018
Short/long f.|38505|Sturge-Weber syndrome (SWS)|SWS|4652
Short/long f.|35258|Restless legs syndrome (RLS)|RLS|4644
Short/long f.|32897|Prader-Willi syndrome (PWS)|PWS|4543
Short/long f.|22169|Isoelectric focusing (IEF)|IEF|4304
Short/long f.|796251|Prieto syndrome (PRS)|PRS|4036
Short/long f.|179224|Basic Radiography Systems (BRS)|BRS|3953
Short/long f.|1572769|MYTHYLSULFONYLMETHANE (MSM)|MSM|3839
Short/long f.|360105|Selective Serotonin Reuptake Inhibitors (SSRIs)|Selective Serotonin Reuptake Inhibitors|3750
Short/long f.|22283|Incontinentia pigmenti achromians (IPA)|IPA|3728
Short/long f.|242387|Treacher Collins syndrome (TCS)|TCS|3604
Short/long f.|430303|Nitroblue tetrazolium dye test (NTD)|NTD|3491
Short/long f.|1456077|Left ventricular assist device (LVAD)|LVAD|3486
Short/long f.|520370|Multifetal pregnancy reduction(s) (MPR)|MPR|3320
Short/long f.|201717|Radial immunodiffusion (RID)|Radial immunodiffusion|3099
Short/long f.|64036|Isophorone diamine (IPD)|IPD|2977
Short/long f.|201983|Dehydroepiandrosterone-sulfate (DHEA-S)|DHEA-S|2953
Short/long f.|1456035|Image guided surgery (IGS)|IGS|2904
Short/long f.|796089|Norman-Roberts syndrome (NRS)|NRS|2753
Short/long f.|16667|Martin-Bell syndrome (MBS)|MBS|2626
Short/long f.|34601|Radiology Information Systems (RIS)|RIS|2462
Short/long f.|38522|Subacute sclerosing panencephalitis (SSPE)|sclerosing panencephalitis|2374
Short/long f.|24814|Marinesco-Sjogren syndrome (MSS)|MSS|2142
Short/long f.|265210|Marshall-Smith syndrome (MSS)|MSS|2142
Short/long f.|23374|Lesch-Nyhan syndrome (LNS)|LNS|2120
Short/long f.|205711|Pelizaeus-Merzbacher disease (PMD)|PMD|2051
Short/long f.|2395|Alzheimer-type dementia (ADT)|ADT|2031
Short/long f.|131513|N-Terminal Peptide of POMC (NPP)|NPP|2007
Short/long f.|24477|Magnesium oxide (MgO)|MgO|1835
Short/long f.|265246|Townes-Brocks syndrome (TBS)|TBS|1763
Short/long f.|1571617|X (inactive)-specific transcript (XIST)|XIST|1719
Short/long f.|302275|Isocitric dehydrogenase (IDH)|IDH|1600
Short/long f.|36391|Schwartz-Jampel syndrome (SJS)|SJS|1505
Short/long f.|79365|Fluorescence polarization immunoassay (FPIA)|FPIA|1467
Short/long f.|1719627|Hypoxic-ischemic encephalopathy (HIE)|HIE|1411
END TOP 50 FREQUENT

RANDOM CASES Short/long f.:
Short/long f.|1725182|Polibar Rapid (P/P)|P/P|13594
Short/long f.|85280|Alagille syndrome (AGS)|AGS|7722
Short/long f.|175693|Silver-Russell syndrome (SRS)|SRS|6679
Short/long f.|246450|M protein, Mouse hepatitis virus (MHV)|MHV|6203
Short/long f.|360105|Selective Serotonin Reuptake Inhibitors (SSRIs)|SSRIs|5960
Short/long f.|1456025|Intravascular ultrasound (IVUS)|Intravascular ultrasound|5417
Short/long f.|182281|Picture Archiving and Communication Systems (PACS)|PACS|5390
Short/long f.|1456025|Intravascular ultrasound (IVUS)|IVUS|5377
Short/long f.|38505|Sturge-Weber syndrome (SWS)|SWS|4652
Short/long f.|35258|Restless legs syndrome (RLS)|RLS|4644

Short/long f.|32897|Prader-Willi syndrome (PWS)|PWS|4543
Short/long f.|179224|Basic Radiography Systems (BRS)|BRS|3953
Short/long f.|360105|Selective Serotonin Reuptake Inhibitors (SSRIs)|Selective Serotonin Reuptake Inhibitors|3750
Short/long f.|242387|Treacher Collins syndrome (TCS)|TCS|3604
Short/long f.|201717|Radial immunodiffusion (RID)|Radial immunodiffusion|3099
Short/long f.|1456035|Image guided surgery (IGS)|IGS|2904
Short/long f.|34601|Radiology Information Systems (RIS)|RIS|2462
Short/long f.|24814|Marinesco-Sjogren syndrome (MSS)|MSS|2142
Short/long f.|265210|Marshall-Smith syndrome (MSS)|MSS|2142
Short/long f.|131513|N-Terminal Peptide of POMC (NPP)|NPP|2007
Short/long f.|24477|Magnesium oxide (MgO)|MgO|1835
Short/long f.|302275|Isocitric dehydrogenase (IDH)|IDH|1600
Short/long f.|1719627|Hypoxic-ischemic encephalopathy (HIE)|HIE|1411
Short/long f.|1473|Adenosine triphosphatase (ATP'ase)|ATP'ase|1354
Short/long f.|1555992|Nurse Massage Therapist (NMT)|NMT|1341
Short/long f.|1522251|Dentatorubropallidoluysian Atrophy (DRPLA)|DRPLA|1065
Short/long f.|12236|DiGeorge syndrome (DGS)|DGS|957
Short/long f.|994142|MICROSURGICAL EPIDIDYMAL SPERM ASPIRATION (MESA)|MESA|939
Short/long f.|1519341|Control of skeletal myogenesis by HDAC & calcium/calmodulin-dependent kinase (CaMK)|calmodulin-dependent kinase|881
Short/long f.|796033|Marden-Walker syndrome (MWS)|MWS|879
Short/long f.|1009953|Isla Vista virus (ILV)|ILV|863
Short/long f.|231985|Mean maximum expiratory flow (MMEF)|maximum expiratory flow|639
Short/long f.|1561921|Non-ST elevation myocardial infarction (NSTEMI)|NSTEMI|638
Short/long f.|410174|Fukuyama congenital muscular dystrophy (FCMD)|FCMD|614
Short/long f.|1561925|Posterior lumbar interbody fusion (PLIF)|PLIF|607
Short/long f.|795810|Wolf-Hirschhorn syndrome (WHS)|WHS|456
Short/long f.|257253|COP9 Signalosome (CSN)|COP9 Signalosome|386
Short/long f.|1000312|Lettuce mosaic potyvirus (LMV)|LMV|377
Short/long f.|1740601|Epidural Spinal Electrostimulatory Systems (ESES)|ESES|376
Short/long f.|1553182|Microparticle enzyme immunoassay (MEIA)|Microparticle enzyme immunoassay|359
Short/long f.|265221|Walker-Warburg syndrome (WWS)|WWS|316
Short/long f.|1449939|Excimer Laser Subepithelial Abalation (ELSA)|ELSA|307
Short/long f.|1456079|Right ventricular assist device (RVAD)|RVAD|280
Short/long f.|1275127|Hyper-IgD periodic fever syndrome (HIDS)|HIDS|265
Short/long f.|344447|VISUAL REINFORCEMENT AUDIOMETRY (VRA)|VRA|260
Short/long f.|1719788|Episodic ataxia type 1 (EA1)|EA1|248
Short/long f.|1561926|Transforaminal lumbar interbody fusion (TLIF)|TLIF|215
Short/long f.|1275091|Ichthyosis follicularis with alopecia and photophobia (IFAP)|IFAP|210
Short/long f.|1705480|Anti-diuretic hormone (ADH)|Anti-diuretic hormone|208
Short/long f.|751774|Periodic limb movement disorder (PLMD)|PLMD|201
Short/long f.|311389|Nonspecific urethritis (NSU)|NSU|181
Short/long f.|10964|Dandy-Walker syndrome (DWS)|DWS|177
Short/long f.|1579361|Serotonin and Norepinephrine Reuptake Inhibitors (SNRIs)|SNRIs|176
Short/long f.|265326|Ruvalcaba-Myhre-Smith syndrome (RMSS)|RMSS|153
Short/long f.|201450|Anti-complement immunofluorescence (ACIF)|ACIF|149
Short/long f.|265219|Miller-Dieker lissencephaly syndrome (MDLS)|MDLS|149
Short/long f.|81714|Extracellular Matrix Metalloproteinase Inducer (EMMPRIN)|Extracellular Matrix Metalloproteinase Inducer|139
Short/long f.|202237|Triiodothyronine T3; total (TT-3)|TT-3|123
Short/long f.|441259|Limited contact dynamic compression plate (LC-DCP)|LC-DCP|110
Short/long f.|796117|Pitt-Rogers-Danks syndrome (PRDS)|PRDS|108
Short/long f.|796250|Partington syndrome (PRTS)|PRTS|91
Short/long f.|13958|Emergency Department Information Systems (EDIS)|EDIS|90
Short/long f.|898752|Scaffold Attachment Factor A (SAF-A)|SAF-A|83
Short/long f.|600664|Personal Narratives (PT)|Personal Narratives|81

Short/long f.|201450|Anti-complement immunofluorescence (ACIF)|Anti-complement immunofluorescence|70
Short/long f.|795889|Allan-Herndon-Dudley syndrome (AHDS)|AHDS|61
Short/long f.|1516628|Clinical Research Curriculum Award (CRCA)|CRCA|57
Short/long f.|1421164|trophinin associated protein (tastin)|tastin|48
Short/long f.|1697856|Minimum Lethal Concentration (MLC)|Minimum Lethal Concentration|48
Short/long f.|753143|Large Neutral Amino Acid Transporter (LNAA)|Large Neutral Amino Acid Transporter|40
Short/long f.|13264|X-linked dilated cardiomyopathy (XLCM)|XLCM|39
Short/long f.|813025|Knee Orthosis (KO)|Knee Orthosis|39
Short/long f.|1000332|Sunn-hemp Mosaic Virus (SHMV)|SHMV|39
Short/long f.|1135356|Prostatic intraepithelial neoplasia II (PIN II)|PIN II|38
Short/long f.|1738380|Integrated Hospital Information Systems (IHIS)|IHIS|36
Short/long f.|445795|Non-cholera vibrio (NCV)|Non-cholera vibrio|33
Short/long f.|753512|Telomere End-Binding Protein (TEBP)|Telomere End-Binding Protein|33
Short/long f.|1552426|Federally Qualified Health Center (FQHC)|Federally Qualified Health Center|32
Short/long f.|1552426|Federally Qualified Health Center (FQHC)|FQHC|30
Short/long f.|949918|Patient Handout (PT)|Patient Handout|26
Short/long f.|1013626|Strawberry Vein Banding Caulimovirus (SVBV)|SVBV|25
Short/long f.|1467040|Ixodes ricinus endosymbiont 1 (IricES1)|IricES1|17
Short/long f.|1617827|DYNAMIC INFRARED BLOOD PERFUSION IMAGING (DIRI)|DIRI|16
Short/long f.|1225498|Haemobartonella felis small form (Hfsm)|Hfsm|15
Short/long f.|175696|X-linked Opitz syndrome (XLOS)|XLOS|11
Short/long f.|1005579|El Moro Canyon hantavirus (ELMC)|ELMC|11
Short/long f.|1738380|Integrated Hospital Information Systems (IHIS)|Integrated Hospital Information Systems|11
Short/long f.|202237|Triiodothyronine T3; total (TT-3)|Triiodothyronine T3; total|10
Short/long f.|339026|Intra-capsular cataract extraction (ICCE)|Intra-capsular cataract extraction|10
Short/long f.|1516767|Comprehensive Minority Biomedical Branch (CMBB)|CMBB|10
Short/long f.|1275091|Ichthyosis follicularis with alopecia and photophobia (IFAP)|Ichthyosis follicularis with alopecia and photophobia|7
Short/long f.|1021993|Prevotella intermedia / Prevotella nigrescens-like organism (PINLO)|PINLO|6
Short/long f.|76313|Tetramethylthiuram monosulphide (TMTM)|Tetramethylthiuram monosulphide|5
Short/long f.|1547165|Systemized Nomenclature of Medicine (SNOMED)|Systemized Nomenclature of Medicine|5
Short/long f.|131513|N-Terminal Peptide of POMC (NPP)|N-Terminal Peptide of POMC|4
Short/long f.|38091|Slow reacting substance-A (SRS-A)|Slow reacting substance-A|3
Short/long f.|544481|Desoxynucleic acid (DNA)|Desoxynucleic acid|2
Short/long f.|1005596|Pear vein yellows-associated virus (PVYV)|PVYV|2
Short/long f.|1736751|Transluminal Extraction Catheters (TEC)|Transluminal Extraction Catheters|2
Short/long f.|813011|Cervical-Thoracic-Lumbar-Sacral Orthosis (CTLSO)|CTLSO|1
END RANDOM CASES

TOP 50 FREQUENT Angular br.:
Angular br.|232011|P_{PL}|PPL|1803
Angular br.|524675|Eisenia <Annelida>|Eisenia|918
Angular br.|1056300|Eisenia <Phaeophyceae>|Eisenia|918
Angular br.|995956|thermophilic bacterium <strain PS3>|thermophilic bacterium|868
Angular br.|303611|^{99m}Technetium|99mTechnetium|810
Angular br.|872916|¹³³Xenon|133Xenon|720
Angular br.|1011491|Albimanus <section>|Albimanus|713
Angular br.|1011494|Albimanus <series>|Albimanus|713
Angular br.|303403|¹¹¹Indium|111Indium|694
Angular br.|1060232|Hua <angiosperm>|Hua|529
Angular br.|1191716|Hua <Mollusca>|Hua|529
Angular br.|162552|Djungarian hamster <Phodopus campbelli>|Djungarian hamster|485
Angular br.|162553|Djungarian hamster <Phodopus sungorus>|Djungarian hamster|485
Angular br.|325803|Arenaria <Aves>|Arenaria|467
Angular br.|996717|Arenaria <Caryophyllaceae>|Arenaria|467
Angular br.|303212|⁵¹Chromium|51Chromium|419

Angular br.|796396|¹²⁵Iodine|125Iodine|385
Angular br.|1013151|Microstoma <fungus>|Microstoma|310
Angular br.|1068712|Reticulitermes <subgenus>|Reticulitermes|301
Angular br.|1082068|Reticulitermes <genus>|Reticulitermes|301
Angular br.|303322|²⁰¹Thallium|201Thallium|270
Angular br.|1018938|Bursaria <Ciliata>|Bursaria|264
Angular br.|1048829|Bursaria <angiosperm>|Bursaria|264
Angular br.|998219|white shrimp <Penaeus vannamei>|white shrimp|258
Angular br.|1029333|Catla <genus name>|Catla|245
Angular br.|303225|⁶⁷Gallium|67Gallium|235
Angular br.|303383|⁴⁵Calcium|45Calcium|229
Angular br.|1028257|Tritonia <Gastropoda>|Tritonia|227
Angular br.|1192547|Tritonia <angiosperm>|Tritonia|227
Angular br.|997465|Nectria <ascomycete>|Nectria|188
Angular br.|1224467|Nectria <echinoderm>|Nectria|188
Angular br.|1621251|Cota <grasshopper>|Cota|188
Angular br.|1622359|Cota <angiosperm>|Cota|188
Angular br.|1002429|tobacco budworm <Helicoverpa armigera>|tobacco budworm|187
Angular br.|1083100|Megacephala <genus>|Megacephala|178
Angular br.|1643168|Megacephala <subgenus>|Megacephala|178
Angular br.|1060212|Armigeres <genus>|Armigeres|161
Angular br.|1632324|Armigeres <subgenus>|Armigeres|161
Angular br.|231900|P_{MUS}|PMUS|160
Angular br.|999023|Eleutherodactylus <genus>|Eleutherodactylus|156
Angular br.|1234762|Eleutherodactylus <subgenus>|Eleutherodactylus|156
Angular br.|1204312|two-spotted spider mite <Tetranychus evansi>|two-spotted spider mite|152
Angular br.|303029|¹³¹Iodine|131Iodine|150
Angular br.|1206422|eelpout <Lycodes toyamensis>|eelpout|142
Angular br.|1038552|Poria cocos <fungus>|Poria cocos|136
Angular br.|1206419|rover <Emmelichthys struhsakeri>|rover|136
Angular br.|303558|⁸⁶Rubidium|86Rubidium|127
Angular br.|1025514|Charybdis <crab>|Charybdis|122
Angular br.|1035189|Charybdis <angiosperm>|Charybdis|122
Angular br.|1008036|Cystophora <Mammalia>|Cystophora|121
END TOP 50 FREQUENT

RANDOM CASES Angular br.:
Angular br.|524675|Eisenia <Annelida>|Eisenia|918
Angular br.|995956|thermophilic bacterium <strain PS3>|thermophilic bacterium|868
Angular br.|303611|^{99m}Technetium|99mTechnetium|810
Angular br.|1011491|Albimanus <section>|Albimanus|713
Angular br.|1060232|Hua <angiosperm>|Hua|529
Angular br.|1048829|Bursaria <angiosperm>|Bursaria|264
Angular br.|1622359|Cota <angiosperm>|Cota|188
Angular br.|1008036|Cystophora <Mammalia>|Cystophora|121
Angular br.|1057934|Pandora <bivalve>|Pandora|108
Angular br.|1067170|Uranotaenia <genus>|Uranotaenia|94
Angular br.|969745|yam-bean <P. tuberosus>|yam-bean|93
Angular br.|1643579|Oryctes <angiosperm>|Oryctes|80
Angular br.|1046434|Lagurus <angiosperm>|Lagurus|66
Angular br.|1229655|Erato <gastropod>|Erato|65
Angular br.|1000522|Danaus <genus>|Danaus|54
Angular br.|1224449|ibop <P. ruscifolia>|ibop|51
Angular br.|1033747|Pholidota <orchid>|Pholidota|47
Angular br.|1048413|Uronema <chlorophycean alga>|Uronema|45
Angular br.|1206660|Melanotus <fungus>|Melanotus|35

Angular br.|1209699|Melanotus <beetle>|Melanotus|35
Angular br.|1624991|Scandia <hydrozoan>|Scandia|30
Angular br.|1627659|Ristella <Squamata>|Ristella|29
Angular br.|303691|⁸⁵Krypton|85Krypton|28
Angular br.|1231296|Chondrilla <sponge>|Chondrilla|27
Angular br.|1635828|Unionicola <subgenus>|Unionicola|20
Angular br.|999224|lesser black-backed gull <fuscus>|lesser black-backed gull|16
Angular br.|1033577|Agathis <Hymenoptera>|Agathis|16
Angular br.|302995|¹⁸Fluorine|18Fluorine|15
Angular br.|303220|⁵⁹Iron|59Iron|15
Angular br.|1011493|Argyritarsis <series>|Argyritarsis|15
Angular br.|1013071|Bonellia <echiuran worm>|Bonellia|14
Angular br.|1071302|Tetraspora <Myxozoa>|Tetraspora|14
Angular br.|1188251|Huia <frog>|Huia|14
Angular br.|1632325|Bonellia <angiosperm>|Bonellia|14
Angular br.|1038164|Turbinaria <Phaeophyceae>|Turbinaria|13
Angular br.|1041800|Chaetosphaeria <Sordariales>|Chaetosphaeria|12
Angular br.|1056250|Diplodiscus <angiosperm>|Diplodiscus|12
Angular br.|1205808|Paphia <angiosperm>|Paphia|12
Angular br.|1207445|Dacrydium <mytilid bivalve>|Dacrydium|12
Angular br.|1009490|Heterocheilidae <nematode>|Heterocheilidae|10
Angular br.|1196689|Eulalia <Andropogoneae>|Eulalia|10
Angular br.|1027101|Crucibulum <fungus>|Crucibulum|9
Angular br.|996491|Pedinomonas <Chlorophyta>|Pedinomonas|8
Angular br.|1078653|smaller tea tortrix <Adoxophyes sp. KSB-2001>|smaller tea tortrix|8
Angular br.|302044|Blood group A₁B|Blood group A1B|6
Angular br.|303502|^{195m}Platinum|195mPlatinum|6
Angular br.|1007284|Schlechtendalia <insect>|Schlechtendalia|6
Angular br.|1009617|Schlechtendalia <plant>|Schlechtendalia|6
Angular br.|1071963|Ambrosiella <Ophiostomataceae>|Ambrosiella|6
Angular br.|1548612|<timing>C (_cum_)<meal>|C (_cum_)|6
Angular br.|1548622|every <integer> weeks|every weeks|6
Angular br.|1645293|Ophidion <cusk-eel>|Ophidion|6
Angular br.|1648352|Taenidia <beetle>|Taenidia|6
Angular br.|1010876|Gambelia <Vertebrata>|Gambelia|5
Angular br.|1032458|Macroglossinae <bats>|Macroglossinae|5
Angular br.|1049277|Gambelia <angiosperm>|Gambelia|5
Angular br.|1200526|Citrus mosaic virus <Badnavirus>|Citrus mosaic virus|5
Angular br.|1254144|low GC gram-positives<blast31968>|low GC gram-positives|5
Angular br.|1635608|Dyscophus <cricket>|Dyscophus|5
Angular br.|303553|⁸¹Rubidium|81Rubidium|4
Angular br.|1032702|Holopedium <crustacean>|Holopedium|4
Angular br.|1086389|Eunapius <sponge>|Eunapius|4
Angular br.|1208695|Orthosia <angiosperm>|Orthosia|4
Angular br.|1548620|every <integer> minutes|every minutes|4
Angular br.|303276|⁴⁰Potassium|40Potassium|3
Angular br.|1016008|Triteleia <angiosperm>|Triteleia|3
Angular br.|1028842|Lacrymaria <agaric fungus>|Lacrymaria|3
Angular br.|1060472|Newtonia <Aves>|Newtonia|3
Angular br.|1072382|pine moth <Dendrolimus pini>|pine moth|3
Angular br.|1080646|Eremococcus <bacteria>|Eremococcus|3
Angular br.|1219309|Pileolaria <fungus>|Pileolaria|3
Angular br.|1621246|serpentine leafminer <Liriomyza brassicae>|serpentine leafminer|3
Angular br.|1643170|Mitrella <gastropod>|Mitrella|3
Angular br.|302046|Blood group A₃B|Blood group A3B|2
Angular br.|313894|Blood group antigen Rl^a|Blood group antigen Rla|2

Angular br.|1025017|Alopiinae <gastropod>|Alopiinae|2
Angular br.|1189018|Stelis <angiosperm>|Stelis|2
Angular br.|1198296|Isomeris <angiosperm>|Isomeris|2
Angular br.|1458297|Chaetoderma <mollusk>|Chaetoderma|2
Angular br.|1492131|Ermia <Squamata>|Ermia|2
Angular br.|1632536|mycorrhizal samples <Basidiomycota>|mycorrhizal samples|2
Angular br.|1640447|Hildegardia <grasshopper>|Hildegardia|2
Angular br.|1640448|Himacerus <genus>|Himacerus|2
Angular br.|1641044|Himacerus <subgenus>|Himacerus|2
Angular br.|1642539|mycorrhizal samples <Ascomycota>|mycorrhizal samples|2
Angular br.|302944|¹¹Carbon|11Carbon|1
Angular br.|303324|²⁰⁴Thallium|204Thallium|1
Angular br.|312966|Blood group antigen Mi^a|Blood group antigen Mia|1
Angular br.|313049|Blood group antigen Ul^a|Blood group antigen Ula|1
Angular br.|313815|Blood group antigen Chr^a|Blood group antigen Chra|1
Angular br.|327989|Gonostomatidae <Vertebrata>|Gonostomatidae|1
Angular br.|998560|Leucophenga <genus>|Leucophenga|1
Angular br.|1021990|Schizophragma <angiosperm>|Schizophragma|1
Angular br.|1030413|Uaru <genus>|Uaru|1
Angular br.|1032137|Clionidae <Porifera>|Clionidae|1
Angular br.|1059999|Eurybia <butterfly>|Eurybia|1
Angular br.|1081412|Tovaria <angniosperm>|Tovaria|1
Angular br.|1216825|Genera incertae sedis <Anaeroplasmatales>|Genera incertae sedis|1
Angular br.|1494363|Tanella <Bacteria>|Tanella|1
Angular br.|1632670|Stelligera <angiosperm>|Stelligera|1
END RANDOM CASES

TOP 50 FREQUENT Semantic type:
Semantic type|237587|Dynamics (Group)|Dynamics|148128
Semantic type|521083|Cava (plant)|Cava|31568
Semantic type|237650|Homing (Animal)|Homing|9424
Semantic type|237624|Foraging (Animal)|Foraging|5403
Semantic type|42932|Vocalizations (Animal)|Vocalizations|3497
Semantic type|237895|Strain Differences (Animal)|Strain Differences|3053
Semantic type|237502|Captivity (Animal)|Captivity|1852
Semantic type|597864|mortality statistics (animal)|mortality statistics|1286
Semantic type|598616|mortality statistics (human)|mortality statistics|1286
Semantic type|540671|PLD1 (enzyme)|PLD1|1246
Semantic type|38631|Sucker (Fish)|Sucker|1086
Semantic type|524539|Roach (Fish)|Roach|759
Semantic type|1284149|All bone (tissue)|All bone|664
Semantic type|237770|Parental Behavior (Animal)|Parental Behavior|575
Semantic type|237600|Escape Behavior (Animal)|Escape Behavior|526
Semantic type|237850|Scent Marking (Animal)|Scent Marking|461
Semantic type|183175|Scleral buckle (physical object)|Scleral buckle|458
Semantic type|765067|LRP6 (receptor)|LRP6|428
Semantic type|237732|Migratory Behavior (Animal)|Migratory Behavior|419
Semantic type|33808|Genus Pseudomonas (organism)|Genus Pseudomonas|418
Semantic type|907896|PETH (hormone)|PETH|400
Semantic type|220806|Chemical agent (substance)|Chemical agent|387
Semantic type|37246|Skates (Fish)|Skates|374
Semantic type|598134|fast reaction (chemical)|fast reaction|333
Semantic type|380153|RED1 (enzyme)|RED1|317
Semantic type|1399203|heart; graft (tissue)|heart; graft|316
Semantic type|598137|slow reaction (chemical)|slow reaction|303
Semantic type|257289|CAPP (enzyme)|CAPP|263

Semantic type|597863|morbidity statistics (animal)|morbidity statistics|190
Semantic type|1540929|Von Willebrand factor activity (finding)|Von Willebrand factor activity|185
Semantic type|304207|Bacterial agent (substance)|Bacterial agent|183
Semantic type|529196|TACA (enzyme)|TACA|168
Semantic type|421231|Driving license (finding)|Driving license|163
Semantic type|21102|Implant, device (physical object)|Implant, device|143
Semantic type|180860|Filter, device (physical object)|Filter, device|132
Semantic type|25361|Information Processes (Human)|Information Processes|129
Semantic type|16215|Genus Flavivirus (organism)|Genus Flavivirus|127
Semantic type|38437|Female urinary stress incontinence (finding)|Female urinary stress incontinence|126
Semantic type|1268919|Fungal agent (substance)|Fungal agent|110
Semantic type|1009235|Tipula (Invertebrate)|Tipula|103
Semantic type|237579|Distress Calls (Animal)|Distress Calls|80
Semantic type|528037|RED2 (enzyme)|RED2|80
Semantic type|237550|Courtship Displays (Animal)|Courtship Displays|74
Semantic type|770536|AGGREGATED ALBUMIN(HUMAN)|AGGREGATED ALBUMIN|66
Semantic type|10200|Complaining of cough (finding)|Complaining of cough|62
Semantic type|59957|PPX1 (enzyme)|PPX1|59
Semantic type|1510495|Wheat - grass (plant)|Wheat - grass|58
Semantic type|237746|Nocturnal Behavior (Animal)|Nocturnal Behavior|55
Semantic type|599011|olfactory communication (animal)|olfactory communication|54
Semantic type|795677|VON WILLEBRAND FACTOR COMPLEX (HUMAN)|VON WILLEBRAND FACTOR COMPLEX|47
END TOP 50 FREQUENT

RANDOM CASES Semantic type:
Semantic type|237624|Foraging (Animal)|Foraging|5403
Semantic type|237895|Strain Differences (Animal)|Strain Differences|3053
Semantic type|237502|Captivity (Animal)|Captivity|1852
Semantic type|1284149|All bone (tissue)|All bone|664
Semantic type|237770|Parental Behavior (Animal)|Parental Behavior|575
Semantic type|765067|LRP6 (receptor)|LRP6|428
Semantic type|220806|Chemical agent (substance)|Chemical agent|387
Semantic type|37246|Skates (Fish)|Skates|374
Semantic type|598134|fast reaction (chemical)|fast reaction|333
Semantic type|380153|RED1 (enzyme)|RED1|317
Semantic type|1399203|heart; graft (tissue)|heart; graft|316
Semantic type|598137|slow reaction (chemical)|slow reaction|303
Semantic type|597863|morbidity statistics (animal)|morbidity statistics|190
Semantic type|1540929|Von Willebrand factor activity (finding)|Von Willebrand factor activity|185
Semantic type|529196|TACA (enzyme)|TACA|168
Semantic type|180860|Filter, device (physical object)|Filter, device|132
Semantic type|770536|AGGREGATED ALBUMIN(HUMAN)|AGGREGATED ALBUMIN|66
Semantic type|10200|Complaining of cough (finding)|Complaining of cough|62
Semantic type|59957|PPX1 (enzyme)|PPX1|59
Semantic type|1510495|Wheat - grass (plant)|Wheat - grass|58
Semantic type|599011|olfactory communication (animal)|olfactory communication|54
Semantic type|659150|hChk1 (human)|hChk1|45
Semantic type|13812|Electrode, device (physical object)|Electrode, device|35
Semantic type|180307|Defibrillator, device (physical object)|Defibrillator, device|28
Semantic type|453985|Button, device (physical object)|Button, device|26
Semantic type|175722|Clip, device (physical object)|Clip, device|23
Semantic type|175659|Scale, device (physical object)|Scale, device|20
Semantic type|961072|ACIX (enzyme)|ACIX|20
Semantic type|175723|Band, device (physical object)|Band, device|15
Semantic type|524724|Staple, device (physical object)|Staple, device|13

Semantic type|182913|Recorder, device (physical object)|Recorder, device|11
Semantic type|439039|Complaining of vomiting (finding)|Complaining of vomiting|11
Semantic type|75029|spleen fibrinolytic proteinase (human)|spleen fibrinolytic proteinase|10
Semantic type|544170|Lactococcus lactis subspecies cremoris (organism)|Lactococcus lactis subspecies cremoris|10
Semantic type|450148|Full thickness skin autograft (substance)|Full thickness skin autograft|8
Semantic type|39142|Syringe, device (physical object)|Syringe, device|7
Semantic type|182555|Punch, device (physical object)|Punch, device|7
Semantic type|663669|hVti1 (human)|hVti1|7
Semantic type|181627|Ligator, device (physical object)|Ligator, device|6
Semantic type|240841|Widened arterial pulse pressure (finding)|Widened arterial pulse pressure|6
Semantic type|729435|Scouring agent (substance)|Scouring agent|6
Semantic type|475201|Complaining of melena (finding)|Complaining of melena|5
Semantic type|676659|CHIT24 (enzyme)|CHIT24|5
Semantic type|1016685|Didiscus (Plant)|Didiscus|5
Semantic type|4185|Atomizer, device (physical object)|Atomizer, device|4
Semantic type|13890|Elevator, device (physical object)|Elevator, device|4
Semantic type|454169|Collimator, device (physical object)|Collimator, device|4
Semantic type|10246|viral; infection, coxsackie(virus)|viral; infection, coxsackie|3
Semantic type|39288|Tampon, device (physical object)|Tampon, device|3
Semantic type|86337|Feeding tube, device (physical object)|Feeding tube, device|3
Semantic type|392220|Scalpel, device (physical object)|Scalpel, device|3
Semantic type|420911|Antenatal care provider (finding)|Antenatal care provider|3
Semantic type|436556|Complaining of perineal pain (finding)|Complaining of perineal pain|3
Semantic type|175720|Caliper, device (physical object)|Caliper, device|2
Semantic type|180431|Dilator, device (physical object)|Dilator, device|2
Semantic type|184348|Warmer, device (physical object)|Warmer, device|2
Semantic type|336779|Machine, device (physical object)|Machine, device|2
Semantic type|392223|Lamp, device (physical object)|Lamp, device|2
Semantic type|436560|Complaining of stiffness (finding)|Complaining of stiffness|2
Semantic type|436561|Complaining of a pain (finding)|Complaining of a pain|2
Semantic type|439023|Complaining of somnolence (finding)|Complaining of somnolence|2
Semantic type|439030|Complaining of nasal congestion (finding)|Complaining of nasal congestion|2
Semantic type|559571|Finding of at risk (finding)|Finding of at risk|2
Semantic type|752521|cytochrome P-450 CYP2A6 (human)|cytochrome P-450 CYP2A6|2
Semantic type|1142644|cytochrome P-450 CYP3A4 (human)|cytochrome P-450 CYP3A4|2
Semantic type|1296478|Iberian pig breed (organism)|Iberian pig breed|2
Semantic type|14467|Eosinophil, segmented (cell)|Eosinophil, segmented|1
Semantic type|21436|Perfusion pump, device (physical object)|Perfusion pump, device|1
Semantic type|31437|Phenotype finding (finding)|Phenotype finding|1
Semantic type|37643|Soman - nerve agent (substance)|Soman - nerve agent|1
Semantic type|40289|Tissue expander, device (physical object)|Tissue expander, device|1
Semantic type|43016|Walker, device (physical object)|Walker, device|1
Semantic type|163275|Fibrin degradation agent (substance)|Fibrin degradation agent|1
Semantic type|175738|Freezer, device (physical object)|Freezer, device|1
Semantic type|179303|Abdominal binder, device (physical object)|Abdominal binder, device|1
Semantic type|179798|Umbrella catheter, device (physical object)|Umbrella catheter, device|1
Semantic type|180950|Hemostat, device (physical object)|Hemostat, device|1
Semantic type|181107|Hammer, device (physical object)|Hammer, device|1
Semantic type|181670|Lithotripter, device (physical object)|Lithotripter, device|1
Semantic type|301380|Cardiac depressant agent (substance)|Cardiac depressant agent|1
Semantic type|336561|Artificial lung, device (physical object)|Artificial lung, device|1
Semantic type|336755|Oven, device (physical object)|Oven, device|1
Semantic type|336756|Microwave oven, device (physical object)|Microwave oven, device|1
Semantic type|336762|Ladder, device (physical object)|Ladder, device|1
Semantic type|336791|Tool, device (physical object)|Tool, device|1
Semantic type|422828|Symptom of lower limb (finding)|Symptom of lower limb|1

Semantic type|439020|Complaining of feeling depressed (finding)|Complaining of feeling depressed|1
Semantic type|450134|Meshed split thickness skin autograft (substance)|Meshed split thickness skin autograft|1
Semantic type|522644|Embolization coil, device (physical object)|Embolization coil, device|1
Semantic type|522649|Detachable balloon, device (physical object)|Detachable balloon, device|1
Semantic type|600103|Finding of blood group (finding)|Finding of blood group|1
Semantic type|677582|Condom, device (physical object)|Condom, device|1
Semantic type|755849|EDTA-Mo (enzyme)|EDTA-Mo|1
Semantic type|1268545|J wire, device (physical object)|J wire, device|1
Semantic type|1296209|Chios sheep breed (organism)|Chios sheep breed|1
Semantic type|1296258|Navajo-Churro sheep breed (organism)|Navajo-Churro sheep breed|1
Semantic type|1296581|Casertana pig breed (organism)|Casertana pig breed|1
Semantic type|1296894|Havanese dog breed (organism)|Havanese dog breed|1
Semantic type|1297017|Awassi sheep breed (organism)|Awassi sheep breed|1
END RANDOM CASES

TOP 50 FREQUENT Begin par.:
Begin par.|1148924|(2-5')oligo(A) synthetase|oligo(A) synthetase|202
Begin par.|1148924|(2-5')oligo(A) synthetase activity|oligo(A) synthetase activity|53
Begin par.|265407|(4)r syndrome|r syndrome|34
Begin par.|795847|(13)r syndrome|r syndrome|34
Begin par.|795855|(15)r syndrome|r syndrome|34
Begin par.|795863|(17)r syndrome|r syndrome|34
Begin par.|795869|(19)r syndrome|r syndrome|34
Begin par.|621538|(COMPADRI)-III protocol|-III protocol|29
Begin par.|1534868|(Hematocrit) or (packed cell volume)|or (packed cell volume)|25
Begin par.|1622825|(Lacrimal disorders NOS) or (epiphora)|or (epiphora)|11
Begin par.|1507299|(PA & L-LAT) UPRIGHT VIEWS|UPRIGHT VIEWS|10
Begin par.|1151603|(protein) methionine-R-sulfoxide reductase|methionine-R-sulfoxide reductase|9
Begin par.|1444127|(1-->3)-beta-D-glucan detection|-beta-D-glucan detection|8
Begin par.|1151171|(R)-aminopropanol dehydrogenase activity|-aminopropanol dehydrogenase activity|6
Begin par.|1151802|(R)-6-hydroxynicotine oxidase activity|-6-hydroxynicotine oxidase activity|3
Begin par.|1151803|(S)-6-hydroxynicotine oxidase activity|-6-hydroxynicotine oxidase activity|3
Begin par.|1151172|(R,R)-butanediol dehydrogenase activity|-butanediol dehydrogenase activity|2
Begin par.|1151282|(S)-2-hydroxy-acid oxidase activity|-2-hydroxy-acid oxidase activity|2
Begin par.|1323951|(4S)-limonene synthase activity|-limonene synthase activity|2
Begin par.|1324056|(S,S)-butanediol dehydrogenase activity|-butanediol dehydrogenase activity|2
Begin par.|1323664|(S)-methylmalonyl-CoA hydrolase activity|-methylmalonyl-CoA hydrolase activity|1
Begin par.|1324049|(R)-2-hydroxyacid dehydrogenase activity|-2-hydroxyacid dehydrogenase activity|1
Begin par.|1324375|(S)-cheilanthifoline synthase activity|-cheilanthifoline synthase activity|1
Begin par.|1324376|(S)-stylopine synthase activity|-stylopine synthase activity|1
Begin par.|1522836|(alpha-N-acetylneuraminyl-2,3-beta-galactosyl-1,3)-N-acetylgalactosaminide alpha-2,6-sialyltransferase activity|-N-acetylgalactosaminide alpha-2,6-sialyltransferase activity|1
Begin par.|1561820|(HPV) DNA test positive|DNA test positive|1
END TOP 50 FREQUENT

TOP 50 FREQUENT End par.:
End par.|237541|Controls (Instrument)|Controls|609492
End par.|237559|Degrees (Educational)|Degrees|598284
End par.|237667|Influence (Interpersonal)|Influence|531725
End par.|935445|Wall (organ part)|Wall|261312
End par.|237881|Significance (Statistical)|Significance|257371
End par.|85415|Outcomes (Treatment)|Outcomes|224578
End par.|237763|Outcomes (Psychotherapeutic)|Outcomes|224578
End par.|1708698|LED (light emitting diode)|LED|189537
End par.|935480|Statistical (PsycINFO Cluster Term)|Statistical|183471
End par.|13878|Particles (Nuclear physics)|Particles|174234

End par.|282173|Space (Astronomy)|Space|173504
End par.|42926|Words (Vocabulary)|Words|170104
End par.|237956|Words (Form Classes)|Words|170104
End par.|237957|Words (Phonetic Units)|Words|170104
End par.|237587|Dynamics (Group)|Dynamics|148128
End par.|237942|Variability (Response)|Variability|148054
End par.|237943|Variability (Stimulus)|Variability|148054
End par.|237470|Amplitude (Response)|Amplitude|142502
End par.|237801|Profiles (Measurement)|Profiles|141775
End par.|42333|Variations (Genetics)|Variations|140570
End par.|178784|Organs (Anatomy)|Organs|139109
End par.|682002|acting (field)|acting|97734
End par.|23745|Linkage (Genetics)|Linkage|94054
End par.|35035|Reliability (Epidemiology)|Reliability|91418
End par.|237828|reliability (research methods)|reliability|91418
End par.|237829|Reliability (Test)|Reliability|91418
End par.|42283|Validity (Epidemiology)|Validity|90773
End par.|237940|validity (research methods)|validity|90773
End par.|237941|Validity (Test)|Validity|90773
End par.|237609|Experiences (Events)|Experiences|89617
End par.|32893|Practices (Psychology)|Practices|89280
End par.|237668|Influences (Social)|Influences|85423
End par.|162775|homology (molecular)|homology|85408
End par.|237882|Similarity (Stimulus)|Similarity|81007
End par.|259967|Residents (Medicine)|Residents|76979
End par.|36849|Sets (Psychology)|Sets|74430
End par.|872379|Subtypes (Disorders)|Subtypes|70097
End par.|679066|Interference (Learning)|Interference|68126
End par.|150775|Networks (Social)|Networks|60077
End par.|25362|Retardation (Mental)|Retardation|56346
End par.|680709|discovery (law)|discovery|56101
End par.|237877|Shifts (Workday)|Shifts|50403
End par.|237521|Complexity (Cognitive)|Complexity|47988
End par.|237522|Complexity (Stimulus)|Complexity|47988
End par.|237523|Complexity (Task)|Complexity|47988
End par.|237501|Candidates (Political)|Candidates|42210
End par.|237533|Conservation (Concept)|Conservation|38439
End par.|237534|Conservation (Ecological Behavior)|Conservation|38439
End par.|679039|conservation (psychology)|conservation|38439
End par.|237680|Institutions (Residential Care)|Institutions|37380
END TOP 50 FREQUENT

RANDOM CASES End par.:
End par.|598002|mechanistic (body)|mechanistic|21532
End par.|597336|QTLs (Quantitative Trait Loci)|QTLs|5171
End par.|1707894|Reed (electrically open)|Reed|4907
End par.|237952|Watson (John Broadus)|Watson|4477
End par.|1388021|venous; return (anomaly)|venous; return|4407
End par.|599218|random coil (protein)|random coil|2684
End par.|3907|arthus (phenomenon or reaction)|arthus|1382
End par.|1515662|inv(16)(p13;q22)|inv(16)|1093
End par.|237692|Item Analysis (Statistical)|Item Analysis|638
End par.|237693|Item Analysis (Test)|Item Analysis|638
End par.|598218|system analysis (computer)|system analysis|579
End par.|1422611|glycoprotein VI (platelet)|glycoprotein VI|325
End par.|447640|Pars nervosa (hypophysis)|Pars nervosa|266

End par.|1414558|Fukuyama type congenital muscular dystrophy (fukutin)|Fukuyama type congenital muscular dystrophy|254
End par.|260226|Scattering length (Nuclear physics)|Scattering length|252
End par.|599378|root resection (dental)|root resection|208
End par.|22827|Cell, L (Cell Line)|Cell, L|182
End par.|237599|Erikson (Erik)|Erikson|165
End par.|1515767|t(3;6)(p13;q25)|t(3;6)|112
End par.|1721006|EPPK (Epidermolytic Palmoplantar Keratoderma)|EPPK|86
End par.|1268806|Increase in CD4 cells (helper cells)|Increase in CD4 cells|75
End par.|1509602|PEN INJECTOR (ML)|PEN INJECTOR|69
End par.|1556364|Grade 3 Fatigue (asthenia, lethargy, malaise)|Grade 3 Fatigue|66
End par.|455939|Antenatal risk factors (situation)|Antenatal risk factors|64
End par.|178484|feeding center (brain)|feeding center|58
End par.|1413089|calmodulin 2 (phosphorylase kinase, delta)|calmodulin 2|57
End par.|1399540|herpes; simplex, hepatitis (manifestation)|herpes; simplex, hepatitis|52
End par.|392673|Adaptation, function (observable entity)|Adaptation, function|41
End par.|1515782|t(7;10)(q34;q24)|t(7;10)|40
End par.|559156|History of - vertigo (situation)|History of - vertigo|37
End par.|1402851|melanoma; liver (primary)|melanoma; liver|36
End par.|1326884|inactivation of MAPK (mating sensu Fungi)|inactivation of MAPK|33
End par.|179423|CIRCUIT, BREATHING (W CONNECTOR, ADAPTOR, Y PIECE)|CIRCUIT, BREATHING|30
End par.|929632|Lamina basalis (Corpus ciliare)|Lamina basalis|28
End par.|21852|Intestine - Small Intestine (MMHCC)|Intestine - Small Intestine|19
End par.|175723|Band, device (physical object)|Band, device|15
End par.|333441|colloid body (eye)|colloid body|14
End par.|600655|Laboratory Manuals (PT)|Laboratory Manuals|14
End par.|1416699|karyopherin alpha 1 (importin alpha 5)|karyopherin alpha 1|12
End par.|995841|Streptoverticillium sp. (strain JCM 4673)|Streptoverticillium sp.|9
End par.|21621|Medicine Institutes (U.S.)|Medicine Institutes|8
End par.|1413689|carnitine palmitoyltransferase 1B (muscle)|carnitine palmitoyltransferase 1B|8
End par.|1538552|odd-skipped related 2 (Drosophila)|odd-skipped related 2|8
End par.|37864|Spermatogenesis, function (observable entity)|Spermatogenesis, function|7
End par.|42226|Aspirator, device (physical object)|Aspirator, device|7
End par.|263859|Synovitis acne pustulosis hyperostosis osteomyelitis syndrome (disorder)|Synovitis acne pustulosis hyperostosis osteomyelitis syndrome|7
End par.|1417589|NGFI-A binding protein 2 (EGR1 binding protein 2)|NGFI-A binding protein 2|7
End par.|1418308|BH-protocadherin (brain-heart)|BH-protocadherin|7
End par.|1396424|encephalitis; influenza (manifestation)|encephalitis; influenza|6
End par.|1420006|sarcoglycan, beta (43kDa dystrophin-associated glycoprotein)|sarcoglycan, beta|6
End par.|1272647|>10 mitoses per 10 HPF (score = 3)|>10 mitoses per 10 HPF|5
End par.|1420744|tight junction protein 1 (zona occludens 1)|tight junction protein 1|5
End par.|226036|Ramus lateralis (arteria coronaria sinistra)|Ramus lateralis|4
End par.|375774|Need for Hormone replacement therapy (postmenopausal)|Need for Hormone replacement therapy|4
End par.|1037525|16SrVI (Clover proliferation group)|16SrVI|4
End par.|1079540|Anguina sp. (Astrebla)|Anguina sp.|4
End par.|27472|National Research Councils (U.S.)|National Research Councils|3
End par.|32897|Prader-Labhart-Willi (PLW) syndrome (PLWS)|Prader-Labhart-Willi (PLW) syndrome|3
End par.|455554|History of - urinary disease (situation)|History of - urinary disease|3
End par.|492942|SET, TRANSFER (BLOOD/PLASMA)|SET, TRANSFER|3
End par.|513124|Monitor fluid balance (e.g., intake/output and daily weight)|Monitor fluid balance|3
End par.|919481|v-maf musculoaponeurotic fibrosarcoma oncogene homolog (avian)|v-maf musculoaponeurotic fibrosarcoma oncogene homolog|3
End par.|1135614|LSA(2)-L(2)|LSA(2)-L|3
End par.|1391847|candidiasis; pneumonia (manifestation)|candidiasis; pneumonia|3
End par.|1394251|glomerulonephritis; cryoglobulinemia (manifestation)|glomerulonephritis; cryoglobulinemia|3
End par.|15944|Membrane Premature Rupture (Pregnancy)|Membrane Premature Rupture|2

End par.|181736|Markers, Magnetic Resonance Imaging (MRI)|Markers, Magnetic Resonance Imaging|2
End par.|194921|Unilateral epididymovasostomy (procedure)|Unilateral epididymovasostomy|2
End par.|239589|Pain;finger(s)|Pain;finger|2
End par.|738952|Ramus ascendens (A. circumflexa femoris lateralis)|Ramus ascendens|2
End par.|812267|v-erb-a erythroblastic leukemia viral oncogene homolog 4 (avian)|v-erb-a erythroblastic leukemia viral oncogene homolog 4|2
End par.|1297306|Sorraia horse breed (organism)|Sorraia horse breed|2
End par.|1332158|Acute Promyelocytic Leukemia with t(11;17)(q13;q21)|Acute Promyelocytic Leukemia with t(11;17)|2
End par.|1401425|catheter; inflammation (suprapubic)|catheter; inflammation|2
End par.|1420712|TGFB-induced factor (TALE family homeobox)|TGFB-induced factor|2
End par.|1423775|ADP-ribosylation factor guanine nucleotide-exchange factor 2 (brefeldin A-inhibited)|ADP-ribosylation factor guanine nucleotide-exchange factor 2|2
End par.|1522391|Hindlimb structure (body structure)|Hindlimb structure|2
End par.|770|teeth; abnormal (form) (size)|teeth; abnormal (form)|1
End par.|11881|diabetes; nephrosis (manifestation)|diabetes; nephrosis|1
End par.|229178|Facies anterior (Iris)|Facies anterior|1
End par.|242855|Congenital atresia of the pulmonary valve (disorder)|Congenital atresia of the pulmonary valve|1
End par.|302097|Lymphocyte production, function (observable entity)|Lymphocyte production, function|1
End par.|319476|Tobacco mosaic virus group (Plant hosts)|Tobacco mosaic virus group|1
End par.|424884|Family history: Maternal grandfather (situation)|Family history: Maternal grandfather|1
End par.|559491|upper limb; sprain (strain)|upper limb; sprain|1
End par.|677582|Condom, device (physical object)|Condom, device|1
End par.|850056|Ischaemia;limb(gangrene)|Ischaemia;limb|1
End par.|1003850|Bacterial sp. (strain ALV)|Bacterial sp.|1
End par.|1388705|arthropathy; psoriasis (manifestation)|arthropathy; psoriasis|1
End par.|1390723|mixed; bronchitis (simple and mucopurulent)|mixed; bronchitis|1
End par.|1392541|choroiditis; syphilitic (manifestation)|choroiditis; syphilitic|1
End par.|1394347|cyst; hydatid, liver (manifestation)|cyst; hydatid, liver|1
End par.|1398806|glomerulonephritis; subacute bacterial endocarditis (manifestation)|glomerulonephritis; subacute bacterial endocarditis|1
End par.|1412111|acetyl-Coenzyme A acetyltransferase 1 (acetoacetyl Coenzyme A thiolase)|acetyl-Coenzyme A acetyltransferase 1|1
End par.|1413201|chaperonin containing TCP1, subunit 8 (theta)|chaperonin containing TCP1, subunit 8|1
End par.|1417735|NK2 transcription factor related, locus 2 (Drosophila)|NK2 transcription factor related, locus 2|1
End par.|1423822|l(3)mbt-like (Drosophila)|l(3)mbt-like|1
End par.|1425521|nei endonuclease VIII-like 1 (E. coli)|nei endonuclease VIII-like 1|1
End par.|1428139|hook homolog 3 (Drosophila)|hook homolog 3|1
End par.|1523294|optic placode development (sensu Endopterygota)|optic placode development|1
END RANDOM CASES

TOP 50 FREQUENT Begin br.:
Begin br.|1149951|[pyruvate dehydrogenase (lipoamide)] phosphatase activity|phosphatase activity|20519
Begin br.|1323652|[3-methyl-2-oxobutanoate dehydrogenase (lipoamide)]-phosphatase activity|-phosphatase activity|20519
Begin br.|1323653|[acetyl-CoA carboxylase]-phosphatase activity|-phosphatase activity|20519
Begin br.|1323654|[glycogen-synthase-D] phosphatase activity|phosphatase activity|20519
Begin br.|1323655|[hydroxymethylglutaryl-CoA reductase (NADPH)]-phosphatase activity|-phosphatase activity|20519
Begin br.|1323656|[phosphorylase] phosphatase activity|phosphatase activity|20519
Begin br.|1323657|[pyruvate kinase]-phosphatase activity|-phosphatase activity|20519
Begin br.|1152359|[methionine synthase] reductase activity|reductase activity|11363
Begin br.|848067|[V]Psychological problems|Psychological problems|3044
Begin br.|1149879|[acyl-carrier protein] phosphodiesterase activity|phosphodiesterase activity|2934
Begin br.|260657|[V]Behavioral problems|Behavioral problems|2900
Begin br.|260657|[V]Behavioural problems|Behavioural problems|1482

Begin br.|362063|[V]Prophylactic antibiotic|Prophylactic antibiotic|1284
Begin br.|1623440|[formate-C-acetyltransferase]-activating enzyme|-activating enzyme|1202
Begin br.|476200|[Q] Post-ganglionic|Post-ganglionic|606
Begin br.|260823|[V]Dietary counseling|Dietary counseling|521
Begin br.|476170|[Q] Refracture|Refracture|475
Begin br.|677491|[V]Kidney transplanted|Kidney transplanted|415
Begin br.|317572|[Haemophilus] paragallinarum|paragallinarum|413
Begin br.|848067|[V]Mental problems|Mental problems|391
Begin br.|441127|[XA511] CATHETER, BALLOON|CATHETER, BALLOON|352
Begin br.|231796|[D]Respiratory abnormalities|Respiratory abnormalities|285
Begin br.|23908|[V]Liver transplanted|Liver transplanted|281
Begin br.|1261243|[V]Human immunodeficiency virus - negative|Human immunodeficiency virus - negative|259
Begin br.|17525|[M]Giant cell tumours|Giant cell tumours|258
Begin br.|260875|[V]Special investigations|Special investigations|247
Begin br.|6160|[M]Brenner tumors|Brenner tumors|245
Begin br.|260823|[V]Dietary counselling|Dietary counselling|244
Begin br.|543430|[V]Educational problems|Educational problems|213
Begin br.|18812|[V]Heart transplanted|Heart transplanted|191
Begin br.|476073|[M]Papillary neoplasms|Papillary neoplasms|188
Begin br.|481840|[V]Insurance medical|Insurance medical|144
Begin br.|476199|[Q] Pre-ganglionic|Pre-ganglionic|116
Begin br.|581325|[D]Acute vertigo|Acute vertigo|108
Begin br.|362085|[V]Sensitization test|Sensitization test|102
Begin br.|260875|[V]Special examinations|Special examinations|87
Begin br.|260937|[V]Screening for sickle cell disease|Screening for sickle cell disease|86
Begin br.|543420|[V]Lung transplanted|Lung transplanted|86
Begin br.|260576|[V]Infertility management|Infertility management|81
Begin br.|37291|[V]Skin transplanted|Skin transplanted|78
Begin br.|556006|[X]Mental and behavioral disorders|Mental and behavioral disorders|69
Begin br.|32131|[M]Plasma cell tumours|Plasma cell tumours|63
Begin br.|556006|[X]Mental and behavioural disorders|Mental and behavioural disorders|61
Begin br.|476270|[D]Heart symptoms|Heart symptoms|59
Begin br.|481842|[V]Prison medical|Prison medical|59
Begin br.|260733|[V]Routine circumcision|Routine circumcision|56
Begin br.|420029|[V]Screening for iron deficiency|Screening for iron deficiency|54
Begin br.|476181|[Q] Head at risk|Head at risk|54
Begin br.|1205369|[Aquaspirillum] autotrophicum|autotrophicum|54
Begin br.|260581|[V]Birth - type|Birth - type|52
END TOP 50 FREQUENT

RANDOM CASES Begin br.:
Begin br.|1149951|[pyruvate dehydrogenase (lipoamide)] phosphatase activity|phosphatase activity|20519
Begin br.|1323656|[phosphorylase] phosphatase activity|phosphatase activity|20519
Begin br.|1152359|[methionine synthase] reductase activity|reductase activity|11363
Begin br.|1149879|[acyl-carrier protein] phosphodiesterase activity|phosphodiesterase activity|2934
Begin br.|362063|[V]Prophylactic antibiotic|Prophylactic antibiotic|1284
Begin br.|1623440|[formate-C-acetyltransferase]-activating enzyme|-activating enzyme|1202
Begin br.|317572|[Haemophilus] paragallinarum|paragallinarum|413
Begin br.|848067|[V]Mental problems|Mental problems|391
Begin br.|1261243|[V]Human immunodeficiency virus - negative|Human immunodeficiency virus - negative|259
Begin br.|476199|[Q] Pre-ganglionic|Pre-ganglionic|116
Begin br.|543420|[V]Lung transplanted|Lung transplanted|86
Begin br.|260576|[V]Infertility management|Infertility management|81
Begin br.|37291|[V]Skin transplanted|Skin transplanted|78
Begin br.|32131|[M]Plasma cell tumours|Plasma cell tumours|63
Begin br.|556006|[X]Mental and behavioural disorders|Mental and behavioural disorders|61

Begin br.|260733|[V]Routine circumcision|Routine circumcision|56
Begin br.|476181|[Q] Head at risk|Head at risk|54
Begin br.|421162|[V]Meningococcal carrier|Meningococcal carrier|46
Begin br.|1319320|[X]Addictive personality|Addictive personality|39
Begin br.|347946|[D]Gallbladder nonvisualization|Gallbladder nonvisualization|36
Begin br.|419585|[V]Screening for rubella|Screening for rubella|35
Begin br.|481438|[V]Tetanus toxoid vaccination|Tetanus toxoid vaccination|34
Begin br.|421163|[V]Staphylococcal carrier|Staphylococcal carrier|33
Begin br.|785223|[XA850] SYRINGES/NEEDLES|SYRINGES/NEEDLES|31
Begin br.|260538|[V]Family history of hearing loss|Family history of hearing loss|30
Begin br.|421164|[V]Streptococcal carrier|Streptococcal carrier|30
Begin br.|11757|[X]Developmental co - ordination disorder|Developmental co - ordination disorder|28
Begin br.|260914|[V]Routine cervical smear|Routine cervical smear|28
Begin br.|1042438|[Actinomadura] sp. ATCC 39727|sp. ATCC 39727|22
Begin br.|973717|[XA800] INTRAVENOUS SETS|INTRAVENOUS SETS|17
Begin br.|1313923|[V]Screening for malaria|Screening for malaria|16
Begin br.|260823|[V]Dietary surveillance|Dietary surveillance|13
Begin br.|159040|[D]Skin texture changes|Skin texture changes|12
Begin br.|40750|[V]Bone transplanted|Bone transplanted|11
Begin br.|496709|[V]Family history of visual loss|Family history of visual loss|11
Begin br.|476245|[D]Global retardation|Global retardation|10
Begin br.|23440|[M]Erythroleukaemias|Erythroleukaemias|9
Begin br.|476223|[D]Crossed laterality|Crossed laterality|8
Begin br.|481514|[V]Health problems in family|Health problems in family|8
Begin br.|420007|[V]Screening for leprosy|Screening for leprosy|7
Begin br.|476186|[Q] Central disc prolapse|Central disc prolapse|7
Begin br.|589447|[V]Family history of celiac disease|Family history of celiac disease|7
Begin br.|184567|[D]Acute pain (situation)|Acute pain (situation)|6
Begin br.|260657|[V]Other behavioural problems|Other behavioural problems|6
Begin br.|260954|[V]Screening for ischaemic heart disease|Screening for ischaemic heart disease|6
Begin br.|476269|[D]Other cardiovascular symptoms|Other cardiovascular symptoms|6
Begin br.|1034502|[Aquaspirillum] delicatum|delicatum|6
Begin br.|1074757|[Anaerococcus] geminatus|geminatus|6
Begin br.|481868|[V]Screening for Chagas' disease|Screening for Chagas' disease|5
Begin br.|486441|[BL900] BLOOD PRODUCTS, OTHER|BLOOD PRODUCTS, OTHER|5
Begin br.|282607|[M]Blood vessel tumours|Blood vessel tumours|4
Begin br.|474855|[M]Mixed leukaemia|Mixed leukaemia|4
Begin br.|481894|[V]Investigation for celiac disease|Investigation for celiac disease|4
Begin br.|549297|[D]Swelling of ear|Swelling of ear|4
Begin br.|36572|[D]Fit (situation)|Fit (situation)|3
Begin br.|333300|[D]Phagedaena|Phagedaena|3
Begin br.|346617|[X]Malignant neoplasm of digestive organs|Malignant neoplasm of digestive organs|3
Begin br.|362036|[D]Coma and stupor|Coma and stupor|3
Begin br.|481863|[V]Screening for leptospirosis|Screening for leptospirosis|3
Begin br.|481894|[V]Investigation for coeliac disease|Investigation for coeliac disease|3
Begin br.|700631|[V]Occupational therapy and vocational rehabilitation|Occupational therapy and vocational rehabilitation|3
Begin br.|178415|[D] Raised prostatic specific antigen|Raised prostatic specific antigen|2
Begin br.|260339|[V]Contact with communicable disease|Contact with communicable disease|2
Begin br.|348272|[X]Other schistosomiasis|Other schistosomiasis|2
Begin br.|476421|[D]Abnormal pancreatic function test|Abnormal pancreatic function test|2
Begin br.|481870|[V]Screening for trypanosomiasis|Screening for trypanosomiasis|2
Begin br.|481887|[V]Screening for chronic bronchitis|Screening for chronic bronchitis|2
Begin br.|565797|[D]Development, failure in|Development, failure in|2
Begin br.|581299|[D]Submental lump|Submental lump|2
Begin br.|973670|[XA100] BANDAGES/DRESSINGS|BANDAGES/DRESSINGS|2

Begin br.|1273100|[D]Gross motor development delay|Gross motor development delay|2
Begin br.|1792|[D]Old age (situation)|Old age (situation)|1
Begin br.|22408|[X]Arthrosis (disorder)|Arthrosis (disorder)|1
Begin br.|38644|[D]Cot death (situation)|Cot death (situation)|1
Begin br.|151611|[D]Electroencephalogram (EEG) abnormal|Electroencephalogram (EEG) abnormal|1
Begin br.|232488|[D]Colic (situation)|Colic (situation)|1
Begin br.|302487|[V]Periodic pelvic examination|Periodic pelvic examination|1
Begin br.|344329|[D]Collapse (situation)|Collapse (situation)|1
Begin br.|348482|[X]Other hyperphenylalaninemias|Other hyperphenylalaninemias|1
Begin br.|362081|[V]Problems with in-laws|Problems with in-laws|1
Begin br.|420007|[V]Screening for Hansen's disease|Screening for Hansen's disease|1
Begin br.|423633|[D]Parasternal chest pain|Parasternal chest pain|1
Begin br.|476084|[M]Papillary adenomas and adenocarcinomas|Papillary adenomas and adenocarcinomas|1
Begin br.|476115|[M]Sebaceous adenoma and adenocarcinoma|Sebaceous adenoma and adenocarcinoma|1
Begin br.|476319|[D]Nonspecific blood findings|Nonspecific blood findings|1
Begin br.|476492|[D]Antibody titre raised|Antibody titre raised|1
Begin br.|476506|[D]Abnormal cytological findings in cerebrospinal fluid|Abnormal cytological findings in cerebrospinal fluid|1
Begin br.|481452|[V]Other adolescence|Other adolescence|1
Begin br.|481703|[V]Problems with smell|Problems with smell|1
Begin br.|481703|[V]Problems with taste|Problems with taste|1
Begin br.|481870|[V]Screening for sleeping sickness|Screening for sleeping sickness|1
Begin br.|481888|[V]Screening for emphysema|Screening for emphysema|1
Begin br.|481907|[V]Dietary counseling in obesity|Dietary counseling in obesity|1
Begin br.|522055|[D]Electrocardiogram (ECG) abnormal|Electrocardiogram (ECG) abnormal|1
Begin br.|558448|[D]Clubbing of fingers/toes|Clubbing of fingers/toes|1
Begin br.|581326|[D]Clicking thumb|Clicking thumb|1
Begin br.|599986|[V]Genetic counselling (situation)|Genetic counselling (situation)|1
Begin br.|917801|[D]Insomnia (situation)|Insomnia (situation)|1
Begin br.|1531565|[X]Involuntary excessive blinking|Involuntary excessive blinking|1
END RANDOM CASES

TOP 50 FREQUENT End br.:
End br.|600678|Abstracts [Publication Type]|Abstracts|25082
End br.|600644|Collections [Publication Type]|Collections|15893
End br.|600661|Outlines [Publication Type]|Outlines|12827
End br.|600645|Drawings [Publication Type]|Drawings|4756
End br.|887957|Validation Studies [Publication Type]|Validation Studies|1668
End br.|600647|Essays [Publication Type]|Essays|773
End br.|1568011|Study Characteristics [Publication Type]|Study Characteristics|504
End br.|1265139|Shigella flexneri 2a [II:3,4]|Shigella flexneri 2a|339
End br.|282407|Meeting Abstracts [Publication Type]|Meeting Abstracts|234
End br.|1569094|Consumer Health Information [Publication Type]|Consumer Health Information|230
End br.|155876|Acute exacerbation of chronic obstructive pulmonary disease [COPD]|Acute exacerbation of chronic obstructive pulmonary disease|228
End br.|236752|Dementia Due to...[Indicate the General Medical Condition]|Dementia Due to...|225
End br.|376667|Herbals [Publication Type]|Herbals|213
End br.|282409|Legal Cases [Publication Type]|Legal Cases|209
End br.|600651|Handbooks [Publication Type]|Handbooks|203
End br.|1523351|tissue maintenance [MGI:add]|tissue maintenance|149
End br.|29082|Ophthalmic division [V1]|Ophthalmic division|136
End br.|600649|Exhibitions [Publication Type]|Exhibitions|134
End br.|846506|Bi-level positive airway pressure [BiPAP]|Bi-level positive airway pressure|119
End br.|24695|Mandibular division [V3]|Mandibular division|91
End br.|887932|Government Documents [Publication Type]|Government Documents|90
End br.|24955|Maxillary division [V2]|Maxillary division|85

End br.|1150675|gamma-glutamylcysteine synthetase activity [EC:6.3.2.2]|gamma-glutamylcysteine synthetase activity|82
End br.|600664|Personal Narratives [Publication Type]|Personal Narratives|81
End br.|340961|Acquired pure red cell aplasia [erythroblastopenia]|Acquired pure red cell aplasia|80
End br.|600648|Examination Questions [Publication Type]|Examination Questions|74
End br.|1719649|Elevated carcinoembryonic antigen [CEA]|Elevated carcinoembryonic antigen|72
End br.|1179596|Lumbar nerves [L1--L5]|Lumbar nerves|64
End br.|236809|Insomnia Related to...[Indicate the Axis I or Axis II Disorder]|Insomnia Related to...|55
End br.|1719612|Total laparoscopic radical hysterectomy [TLRH]|Total laparoscopic radical hysterectomy|49
End br.|236744|Delirium Due to...[Indicate the General Medical Condition]|Delirium Due to...|44
End br.|887930|Unpublished Documents [Publication Type]|Unpublished Documents|42
End br.|589717|Gluten-free foods [generic]|Gluten-free foods|41
End br.|1276943|Gluten-free foods [generic 1]|Gluten-free foods|41
End br.|354909|Allergic emergencies [no drugs here]|Allergic emergencies|37
End br.|334674|Malignant myelosclerosis [obs]|Malignant myelosclerosis|35
End br.|949918|Patient Handout [Publication Type]|Patient Handout|26
End br.|6413|Burkitt tumour [obs]|Burkitt tumour|22
End br.|1265149|Shigella flexneri 6 [VI:(3,4)]|Shigella flexneri 6|22
End br.|875833|Nedocromil Sodium 2% Ophthalmic Solution [ALOCRIL]|Nedocromil Sodium 2% Ophthalmic Solution|21
End br.|1265137|Shigella flexneri 1b [I: 6 (3,4)]|Shigella flexneri 1b|20
End br.|887929|Unpublished Works [Publication Type]|Unpublished Works|18
End br.|938950|Ketotifen Fumarate 0.025% Ophthalmic Solution [ZADITOR]|Ketotifen Fumarate 0.025% Ophthalmic Solution|18
End br.|1523417|linking collagen [ISBN:0721639976]|linking collagen|18
End br.|1607209|Lamivudine 150 MG / Zidovudine 300 MG [Combivir]|Lamivudine 150 MG / Zidovudine 300 MG|18
End br.|715292|Olopatadine Hydrochloride 0.1% Ophthalmic Solution [PATANOL]|Olopatadine Hydrochloride 0.1% Ophthalmic Solution|17
End br.|236817|Personality Change Due to...[Indicate the General Medical Condition]|Personality Change Due to...|16
End br.|1265142|Shigella flexneri 3a [III:6,7,8]|Shigella flexneri 3a|16
End br.|1605771|Captopril 50 MG / Hydrochlorothiazide 25 MG [Capozide]|Captopril 50 MG / Hydrochlorothiazide 25 MG|16
End br.|600643|Collected Works [Publication Type]|Collected Works|15
END TOP 50 FREQUENT

RANDOM CASES End br.:
End br.|600678|Abstracts [Publication Type]|Abstracts|25082
End br.|600644|Collections [Publication Type]|Collections|15893
End br.|600647|Essays [Publication Type]|Essays|773
End br.|282409|Legal Cases [Publication Type]|Legal Cases|209
End br.|24695|Mandibular division [V3]|Mandibular division|91
End br.|24955|Maxillary division [V2]|Maxillary division|85
End br.|600664|Personal Narratives [Publication Type]|Personal Narratives|81
End br.|340961|Acquired pure red cell aplasia [erythroblastopenia]|Acquired pure red cell aplasia|80
End br.|1719649|Elevated carcinoembryonic antigen [CEA]|Elevated carcinoembryonic antigen|72
End br.|887930|Unpublished Documents [Publication Type]|Unpublished Documents|42
End br.|354909|Allergic emergencies [no drugs here]|Allergic emergencies|37
End br.|949918|Patient Handout [Publication Type]|Patient Handout|26
End br.|875833|Nedocromil Sodium 2% Ophthalmic Solution [ALOCRIL]|Nedocromil Sodium 2% Ophthalmic Solution|21
End br.|1265137|Shigella flexneri 1b [I: 6 (3,4)]|Shigella flexneri 1b|20
End br.|887929|Unpublished Works [Publication Type]|Unpublished Works|18
End br.|938950|Ketotifen Fumarate 0.025% Ophthalmic Solution [ZADITOR]|Ketotifen Fumarate 0.025% Ophthalmic Solution|18
End br.|236817|Personality Change Due to...[Indicate the General Medical Condition]|Personality Change Due to...|16
End br.|1719707|Estrogen receptor negative status [ER-]|Estrogen receptor negative status|15

End br.|600655|Laboratory Manuals [Publication Type]|Laboratory Manuals|14
End br.|1266194|Hodgkin's disease, lymphocyte predominance [obs]|Hodgkin's disease, lymphocyte predominance|13
End br.|1607004|Amlodipine 5 MG / benazepril 10 MG [Lotrel]|Amlodipine 5 MG / benazepril 10 MG|11
End br.|1265140|Shigella flexneri 2b [II:7,8]|Shigella flexneri 2b|10
End br.|1607014|Amlodipine 5 MG / benazepril 20 MG [Lotrel]|Amlodipine 5 MG / benazepril 20 MG|10
End br.|1167476|nitrite reductase complex [NAD(P)H]|nitrite reductase complex|9
End br.|600667|Popular Works [Publication Type]|Popular Works|8
End br.|1320109|Cyclosporine 0.05% Ophthalmic Emulsion [RESTASIS]|Cyclosporine 0.05% Ophthalmic Emulsion|8
End br.|1182936|Floating ribs [XI-XII]|Floating ribs|7
End br.|1320273|Shigella flexneri 5a [V:3,4]|Shigella flexneri 5a|7
End br.|1588570|ezetimibe 10 MG / Simvastatin 20 MG [Vytorin]|ezetimibe 10 MG / Simvastatin 20 MG|7
End br.|236747|Mood Disorder Due to...[Indicate the General Medical Condition]|Mood Disorder Due to...|6
End br.|716277|Brinzolamide 1% Ophthalmic Suspension [AZOPT]|Brinzolamide 1% Ophthalmic Suspension|6
End br.|16142|Pathological fire-setting [pyromania]|Pathological fire-setting|5
End br.|24695|N. mandibularis [Vc; V3]|N. mandibularis|5
End br.|43116|Infantile spinal muscular atrophy, type I [Werdnig-Hoffman]|Infantile spinal muscular atrophy, type I|5
End br.|476491|Laboratory evidence of human immunodeficiency virus [HIV]|Laboratory evidence of human immunodeficiency virus|5
End br.|841481|Excision of arteriovenous malformation [AVM]|Excision of arteriovenous malformation|5
End br.|1323948|3-hydroxyaspartate dehydratase [PMID:10481099, SGD:mcc]|3-hydroxyaspartate dehydratase|5
End br.|1607910|Bisoprolol 2.5 MG / Hydrochlorothiazide 6.25 MG [Ziac]|Bisoprolol 2.5 MG / Hydrochlorothiazide 6.25 MG|5
End br.|1320274|Shigella flexneri 5b [V:7,8]|Shigella flexneri 5b|4
End br.|1719417|Noninvasive programmed electrical stimulation [NIPS]|Noninvasive programmed electrical stimulation|4
End br.|334636|Malignant lymphoma, centroblastic-centrocytic [obs]|Malignant lymphoma, centroblastic-centrocytic|3
End br.|1593422|Acetaminophen 325 MG / Hydrocodone 7.5 MG [Anexsia]|Acetaminophen 325 MG / Hydrocodone 7.5 MG|3
End br.|1595311|Acetaminophen 500 MG / Hydrocodone 5 MG [Anexsia]|Acetaminophen 500 MG / Hydrocodone 5 MG|3
End br.|1595345|Acetaminophen 500 MG / Hydrocodone 5 MG [Lortab 5/500]|Acetaminophen 500 MG / Hydrocodone 5 MG|3
End br.|1602294|Acetaminophen 500 MG / Hydrocodone 5 MG [Vidone]|Acetaminophen 500 MG / Hydrocodone 5 MG|3
End br.|1605458|Acetaminophen 500 MG / Hydrocodone 5 MG [Co-Gesic]|Acetaminophen 500 MG / Hydrocodone 5 MG|3
End br.|1605459|Acetaminophen 500 MG / Hydrocodone 5 MG [DuoCet]|Acetaminophen 500 MG / Hydrocodone 5 MG|3
End br.|1605460|Acetaminophen 500 MG / Hydrocodone 5 MG [Hy-Phen]|Acetaminophen 500 MG / Hydrocodone 5 MG|3
End br.|1605461|Acetaminophen 500 MG / Hydrocodone 5 MG [Panacet]|Acetaminophen 500 MG / Hydrocodone 5 MG|3
End br.|1605462|Acetaminophen 500 MG / Hydrocodone 5 MG [Vanacet]|Acetaminophen 500 MG / Hydrocodone 5 MG|3
End br.|1605463|Acetaminophen 500 MG / Hydrocodone 5 MG [Vicodin]|Acetaminophen 500 MG / Hydrocodone 5 MG|3
End br.|1605471|Acetaminophen 500 MG / Hydrocodone 5 MG [Ceta Plus]|Acetaminophen 500 MG / Hydrocodone 5 MG|3
End br.|1605474|Acetaminophen 500 MG / Hydrocodone 5 MG [Hyco-pap]|Acetaminophen 500 MG / Hydrocodone 5 MG|3
End br.|1605477|Acetaminophen 500 MG / Hydrocodone 5 MG [Hydrogesic]|Acetaminophen 500 MG / Hydrocodone 5 MG|3
End br.|1605478|Acetaminophen 500 MG / Hydrocodone 5 MG [Lorcet HD]|Acetaminophen 500 MG / Hydrocodone 5 MG|3
End br.|1605484|Acetaminophen 500 MG / Hydrocodone 5 MG [Stagesic]|Acetaminophen 500 MG / Hydrocodone 5 MG|3

End br.|1605485|Acetaminophen 500 MG / Hydrocodone 5 MG [T-Gesic]|Acetaminophen 500 MG / Hydrocodone 5 MG|3
End br.|1605486|Acetaminophen 500 MG / Hydrocodone 5 MG [Ugesic]|Acetaminophen 500 MG / Hydrocodone 5 MG|3
End br.|1605912|Amoxicillin 875 MG / Clavulanate 125 MG [Augmentin]|Amoxicillin 875 MG / Clavulanate 125 MG|3
End br.|154275|Selective deficiency of immunoglobulin M [IgM]|Selective deficiency of immunoglobulin M|2
End br.|600660|Nurses' Instruction [Publication Type]|Nurses' Instruction|2
End br.|709968|DINOPROSTONE 0.5 MG GEL [PREPIDIL]|DINOPROSTONE 0.5 MG GEL|2
End br.|716267|Loteprednol Etabonate 0.2% Ophthalmic Suspension [ALREX]|Loteprednol Etabonate 0.2% Ophthalmic Suspension|2
End br.|938958|Pemirolast Potassium 0.1% Ophthalmic Solution [ALAMAST]|Pemirolast Potassium 0.1% Ophthalmic Solution|2
End br.|1182933|Set of ribs [I-XII]|Set of ribs|2
End br.|1265146|Shigella flexneri 4a [IV: (3,4,7,8)]|Shigella flexneri 4a|2
End br.|1523419|MACIT [ISBN:0198599587]|MACIT|2
End br.|1592411|Acetaminophen 325 MG / Oxycodone 5 MG [Percocet]|Acetaminophen 325 MG / Oxycodone 5 MG|2
End br.|1604170|Loratadine 5 MG / Pseudoephedrine 120 MG [Claritin-D]|Loratadine 5 MG / Pseudoephedrine 120 MG|2
End br.|1605713|Acetaminophen 325 MG / Oxycodone 5 MG [Endocet]|Acetaminophen 325 MG / Oxycodone 5 MG|2
End br.|1606451|Atenolol 100 MG / Chlorthalidone 25 MG [Tenoretic]|Atenolol 100 MG / Chlorthalidone 25 MG|2
End br.|1606671|Carbidopa 50 MG / Levodopa 200 MG [Sinemet CR]|Carbidopa 50 MG / Levodopa 200 MG|2
End br.|1607696|acrivastine 8 MG / Pseudoephedrine 60 MG [Semprex-D]|acrivastine 8 MG / Pseudoephedrine 60 MG|2
End br.|1682329|Amlodipine 5 MG / atorvastatin 10 MG [Caduet]|Amlodipine 5 MG / atorvastatin 10 MG|2
End br.|236837|Male Dyspareunia Due to...[Indicate the General Medical Condition]|Male Dyspareunia Due to...|1
End br.|236841|Female Hypoactive Sexual Desire Disorder Due to...[Indicate the General Medical Condition]|Female Hypoactive Sexual Desire Disorder Due to...|1
End br.|476492|Antibody titre raised [D]|Antibody titre raised|1
End br.|495992|Traumatic amputation of both arms [any level]|Traumatic amputation of both arms|1
End br.|558448|Clubbing of fingers/toes [D]|Clubbing of fingers/toes|1
End br.|706980|CLINDAMYCIN PHOSPHATE TOPICAL GEL [CLEOCIN]|CLINDAMYCIN PHOSPHATE TOPICAL GEL|1
End br.|707971|Lodoxamide Tromethamine 0.1% Ophthalmic Solution [ALOMIDE]|Lodoxamide Tromethamine 0.1% Ophthalmic Solution|1
End br.|714099|Prednisolone Sodium Phosphate 1% Ophthalmic Solution [INFLAMASE FORTE]|Prednisolone Sodium Phosphate 1% Ophthalmic Solution|1
End br.|875562|ETANERCEPT 25 MG SUBCUTANEOUS INJECTION [ENBREL]|ETANERCEPT 25 MG SUBCUTANEOUS INJECTION|1
End br.|978950|NIACIN 500 MG TABLET [NIACOR]|NIACIN 500 MG TABLET|1
End br.|1182938|Set of metatarsals [I-V]|Set of metatarsals|1
End br.|1584815|Acetaminophen 500 MG / Oxycodone 5 MG [Taxadone]|Acetaminophen 500 MG / Oxycodone 5 MG|1
End br.|1598909|Acetaminophen 325 MG / Codeine 30 MG [Phenaphen with Codeine]|Acetaminophen 325 MG / Codeine 30 MG|1
End br.|1601035|benazepril 10 MG / Hydrochlorothiazide 12.5 MG [Lotensin HCT]|benazepril 10 MG / Hydrochlorothiazide 12.5 MG|1
End br.|1601064|benazepril 20 MG / Hydrochlorothiazide 25 MG [Lotensin HCT]|benazepril 20 MG / Hydrochlorothiazide 25 MG|1
End br.|1602633|Carbidopa 25 MG / Levodopa 100 MG [Parcopa]|Carbidopa 25 MG / Levodopa 100 MG|1
End br.|1605606|Acetaminophen 500 MG / Oxycodone 5 MG [Roxilox]|Acetaminophen 500 MG / Oxycodone 5 MG|1
End br.|1605607|Acetaminophen 500 MG / Oxycodone 5 MG [Tylox]|Acetaminophen 500 MG / Oxycodone 5 MG|1
End br.|1606368|trandolapril 4 MG / Verapamil 240 MG [Tarka]|trandolapril 4 MG / Verapamil 240 MG|1
End br.|1606369|Enalapril 5 MG / Felodipine 5 MG [Lexxel]|Enalapril 5 MG / Felodipine 5 MG|1
End br.|1606504|Carbidopa 25 MG / Levodopa 100 MG [Atamet]|Carbidopa 25 MG / Levodopa 100 MG|1
End br.|1606650|Carbidopa 25 MG / Levodopa 100 MG [Sinemet CR]|Carbidopa 25 MG / Levodopa 100 MG|1

End br.|1606792|Acetaminophen 325 MG / Hydrocodone 10 MG [Norco]|Acetaminophen 325 MG / Hydrocodone 10 MG|1
End br.|1618263|Ibuprofen 400 MG / Oxycodone 5 MG [Combunox]|Ibuprofen 400 MG / Oxycodone 5 MG|1
End br.|1621354|Ac-MVA pathway [PMID:14517367]|Ac-MVA pathway|1
End br.|1621839|CNS ischemic response [ISBN:072163949 "Textbook of Medical Physiology"]|CNS ischemic response|1
END RANDOM CASES

SUPPRESS RULES

TOP 50 FREQUENT Dosages:
Dosages|1374702|FLUOROURACIL 5 %|FLUOROURACIL 5 %|6643
Dosages|1321990|Amino Acids 1%|Amino Acids 1%|1580
Dosages|988268|Omeprazole 20 MG|Omeprazole 20 MG|1281
Dosages|1123414|Ranitidine 150 MG|Ranitidine 150 MG|746
Dosages|1375274|LIDOCAINE 1 %|LIDOCAINE 1 %|727
Dosages|1374218|CISPLATIN 100 MG|CISPLATIN 100 MG|650
Dosages|1123920|Morphine 10 MG|Morphine 10 MG|643
Dosages|1145828|lansoprazole 30 MG|lansoprazole 30 MG|632
Dosages|984982|Clarithromycin 500 MG|Clarithromycin 500 MG|588
Dosages|1123415|Ranitidine 300 MG|Ranitidine 300 MG|484
Dosages|1381794|IMIQUIMOD 5 %|IMIQUIMOD 5 %|483
Dosages|977735|Imiquimod 5% cream|Imiquimod 5% cream|448
Dosages|992678|Omeprazole 40 MG|Omeprazole 40 MG|445
Dosages|1381699|CHOLESTEROL 3 %|CHOLESTEROL 3 %|439
Dosages|499128|ETOPOSIDE, 100 MG|ETOPOSIDE, 100 MG|426
Dosages|1125128|Etoposide 100 MG|Etoposide 100 MG|426
Dosages|1123916|Morphine 5 MG|Morphine 5 MG|414
Dosages|499109|CISPLATIN, 50 MG|CISPLATIN, 50 MG|409
Dosages|1379116|CISPLATIN 50 MG|CISPLATIN 50 MG|409
Dosages|985489|Diazepam 10 MG|Diazepam 10 MG|387
Dosages|1379772|atorvastatin 10 MG|atorvastatin 10 MG|385
Dosages|983889|Atenolol 50 MG|Atenolol 50 MG|372
Dosages|986112|Fluoxetine 20 MG|Fluoxetine 20 MG|372
Dosages|987276|Losartan 50 MG|Losartan 50 MG|360
Dosages|986405|Haloperidol 1 MG|Haloperidol 1 MG|355
Dosages|983887|Atenolol 100 MG|Atenolol 100 MG|354
Dosages|1305845|Oxygen 1 %|Oxygen 1 %|350
Dosages|1123382|Metoclopramide 10 MG|Metoclopramide 10 MG|346
Dosages|1171050|Naloxone 2 MG|Naloxone 2 MG|342
Dosages|1380261|LATANOPROST 0.005 %|LATANOPROST 0.005 %|337
Dosages|988140|Nifedipine 10 MG|Nifedipine 10 MG|332
Dosages|992254|Diazepam 5 MG|Diazepam 5 MG|330
Dosages|1373368|ALCOHOL 2 %|ALCOHOL 2 %|327
Dosages|1126048|pantoprazole 40 MG|pantoprazole 40 MG|325
Dosages|988141|Nifedipine 20 MG|Nifedipine 20 MG|323
Dosages|1126289|Tamoxifen 20 MG|Tamoxifen 20 MG|323
Dosages|989915|Simvastatin 20 MG|Simvastatin 20 MG|312
Dosages|1123176|Enalapril 10 MG|Enalapril 10 MG|306
Dosages|984915|Cimetidine 400 MG|Cimetidine 400 MG|301
Dosages|984503|Captopril 50 MG|Captopril 50 MG|298
Dosages|1127610|Esomeprazole 40 MG|Esomeprazole 40 MG|298
Dosages|992611|Metronidazole 500 MG|Metronidazole 500 MG|295
Dosages|1123542|Propranolol 80 MG|Propranolol 80 MG|291
Dosages|1378997|DEXAMETHASONE 0.1 %|DEXAMETHASONE 0.1 %|291

Dosages|1123179|Enalapril 20 MG|Enalapril 20 MG|281
Dosages|984502|Captopril 25 MG|Captopril 25 MG|280
Dosages|992428|Ibuprofen 400 MG|Ibuprofen 400 MG|278
Dosages|992015|Aspirin 325 MG|Aspirin 325 MG|275
Dosages|986111|Fluoxetine 10 MG|Fluoxetine 10 MG|267
Dosages|985344|Dexamethasone 1 MG|Dexamethasone 1 MG|266
END TOP 50 FREQUENT

RANDOM CASES Dosages:
Dosages|986526|Hydrochlorothiazide 25 MG|Hydrochlorothiazide 25 MG|265
Dosages|993445|Paroxetine 20 MG|Paroxetine 20 MG|167
Dosages|1145935|montelukast 10 MG|montelukast 10 MG|136
Dosages|987282|Lovastatin 20 MG|Lovastatin 20 MG|120
Dosages|794811|Lidocaine Patch 5%|Lidocaine Patch 5%|99
Dosages|1380056|BRINZOLAMIDE 1 %|BRINZOLAMIDE 1 %|87
Dosages|984555|carvedilol 25 MG|carvedilol 25 MG|67
Dosages|989893|sildenafil 100 MG|sildenafil 100 MG|63
Dosages|1127473|linezolid 600 MG|linezolid 600 MG|59
Dosages|1381822|LIDOCAINE 2.5 %|LIDOCAINE 2.5 %|57
Dosages|1123645|Diltiazem 180 MG|Diltiazem 180 MG|56
Dosages|1379311|PERMETHRIN 5 %|PERMETHRIN 5 %|44
Dosages|975705|Clindamycin phosphate 1%|Clindamycin phosphate 1%|43
Dosages|1128662|pioglitazone 15 MG|pioglitazone 15 MG|28
Dosages|1128686|Methotrexate 7.5 MG|Methotrexate 7.5 MG|27
Dosages|1123651|Tranylcypromine 10 MG|Tranylcypromine 10 MG|25
Dosages|1331116|Bupropion 300 MG|Bupropion 300 MG|24
Dosages|1123613|Acebutolol 200 MG|Acebutolol 200 MG|22
Dosages|987134|Levofloxacin 250 MG|Levofloxacin 250 MG|19
Dosages|989287|Progesterone 2.5 MG|Progesterone 2.5 MG|19
Dosages|1373371|ALCOHOL 22 %|ALCOHOL 22 %|18
Dosages|1124160|Ciprofloxacin 100 MG|Ciprofloxacin 100 MG|17
Dosages|1384462|Ibandronate 2.5 MG|Ibandronate 2.5 MG|16
Dosages|987271|Loratadine 5 MG|Loratadine 5 MG|14
Dosages|1123156|Secobarbital 100 MG|Secobarbital 100 MG|14
Dosages|983810|Ascorbic Acid 200 MG|Ascorbic Acid 200 MG|13
Dosages|1616485|duloxetine 20 MG|duloxetine 20 MG|13
Dosages|1375789|OXYMETAZOLINE 0.025 %|OXYMETAZOLINE 0.025 %|12
Dosages|984275|cabergoline 0.5 MG|cabergoline 0.5 MG|11
Dosages|1128721|Disulfiram 200 MG|Disulfiram 200 MG|11
Dosages|1380017|TRYPSIN 0.01 %|TRYPSIN 0.01 %|11
Dosages|1126847|Amiloride 10 MG|Amiloride 10 MG|10
Dosages|987814|Methylprednisolone 8 MG|Methylprednisolone 8 MG|9
Dosages|1380875|SILVER NITRATE 0.5 %|SILVER NITRATE 0.5 %|9
Dosages|1577190|Eszopiclone 3 MG|Eszopiclone 3 MG|9
Dosages|1381940|HALOTHANE 100 %|HALOTHANE 100 %|8
Dosages|1577177|darifenacin 15 MG|darifenacin 15 MG|8
Dosages|691279|Capsaicin Cream 0.025%|Capsaicin Cream 0.025%|7
Dosages|990431|Sulfasalazine 500 MG|Sulfasalazine 500 MG|7
Dosages|1379605|TERCONAZOLE 0.8 %|TERCONAZOLE 0.8 %|7
Dosages|1178690|Dextromethorphan 40 MG|Dextromethorphan 40 MG|6
Dosages|1380012|ATROPINE SULFATE 0.2 MG|ATROPINE SULFATE 0.2 MG|6
Dosages|1129500|Calcium Citrate 800 MG|Calcium Citrate 800 MG|5
Dosages|1146410|Levobupivacaine 5 MG/ML|Levobupivacaine 5 MG/ML|5
Dosages|1375922|PHENOL 4.5 %|PHENOL 4.5 %|5
Dosages|1379849|SULFUR 3 %|SULFUR 3 %|5
Dosages|978638|Miconazole Nitrate Vaginal Cream 2%|Miconazole Nitrate Vaginal Cream 2%|4

Dosages|984645|Cephradine 500 MG|Cephradine 500 MG|4
Dosages|989615|repaglinide 2 MG|repaglinide 2 MG|4
Dosages|1123390|Mexiletine 150 MG|Mexiletine 150 MG|4
Dosages|1379446|PILOCARPINE HYDROCHLORIDE 5 MG|PILOCARPINE HYDROCHLORIDE 5 MG|4
Dosages|1379944|SALICYLIC ACID 2.5 %|SALICYLIC ACID 2.5 %|4
Dosages|1380020|PROPYLENE GLYCOL 40 %|PROPYLENE GLYCOL 40 %|4
Dosages|976541|Doxepin hydrochloride 5% cream|Doxepin hydrochloride 5% cream|3
Dosages|988710|Phenylalanine 500 MG|Phenylalanine 500 MG|3
Dosages|989278|Prochlorperazine 25 MG|Prochlorperazine 25 MG|3
Dosages|1124357|Amitriptyline 12.5 MG|Amitriptyline 12.5 MG|3
Dosages|1125959|dofetilide 0.5 MG|dofetilide 0.5 MG|3
Dosages|1127782|coenzyme Q10 100 MG|coenzyme Q10 100 MG|3
Dosages|1127870|Diclofenac 12.5 MG|Diclofenac 12.5 MG|3
Dosages|1306382|Etonogestrel 68 MG|Etonogestrel 68 MG|3
Dosages|1374497|DICLOFENAC POTASSIUM 50 MG|DICLOFENAC POTASSIUM 50 MG|3
Dosages|984757|Chlorothiazide 250 MG|Chlorothiazide 250 MG|2
Dosages|985264|Cycloserine 250 MG|Cycloserine 250 MG|2
Dosages|985719|EICOSAPENTAENOIC ACID 180 MG|EICOSAPENTAENOIC ACID 180 MG|2
Dosages|993073|trilostane 30 MG|trilostane 30 MG|2
Dosages|1127432|Risperidone 0.25 MG|Risperidone 0.25 MG|2
Dosages|1128020|Fentanyl 0.005 MG/ML|Fentanyl 0.005 MG/ML|2
Dosages|1132630|Sotalol 40 MG|Sotalol 40 MG|2
Dosages|1132968|Zinc 22.5 MG|Zinc 22.5 MG|2
Dosages|1306684|Dipyridamole 10 MG/ML|Dipyridamole 10 MG/ML|2
Dosages|1380464|PYROGALLOL 25 %|PYROGALLOL 25 %|2
Dosages|1381073|MAGNESIUM SULFATE 80 %|MAGNESIUM SULFATE 80 %|2
Dosages|1381830|PHENYLEPHRINE HYDROCHLORIDE 0.25 %|PHENYLEPHRINE HYDROCHLORIDE 0.25 %|2
Dosages|1581209|VOLTAREN 25 MG|VOLTAREN 25 MG|2
Dosages|781381|Metronidazole 0.75% lotion|Metronidazole 0.75% lotion|1
Dosages|984138|Bile Salts 100 MG|Bile Salts 100 MG|1
Dosages|984378|Calcium Carbonate 300 MG|Calcium Carbonate 300 MG|1
Dosages|986101|FLUORIDE 0.5 MG|FLUORIDE 0.5 MG|1
Dosages|992287|efavirenz 200 MG|efavirenz 200 MG|1
Dosages|1105566|prednisolone 40 MG/ML|prednisolone 40 MG/ML|1
Dosages|1124585|benzonatate 200 MG|benzonatate 200 MG|1
Dosages|1125075|Encainide 35 MG|Encainide 35 MG|1
Dosages|1125198|Guanabenz 8 MG|Guanabenz 8 MG|1
Dosages|1128364|Vitamin B6 1 MG|Vitamin B6 1 MG|1
Dosages|1131994|Vitamin B6 30 MG|Vitamin B6 30 MG|1
Dosages|1134938|Zinc Gluconate 50 MG|Zinc Gluconate 50 MG|1
Dosages|1186852|Papaveretum 15.4 MG/ML|Papaveretum 15.4 MG/ML|1
Dosages|1298364|Betamethasone dipropionate 0.5% ointment|Betamethasone dipropionate 0.5% ointment|1
Dosages|1306336|Ceftazidime 10 MG/ML|Ceftazidime 10 MG/ML|1
Dosages|1306667|fenbufen 600 MG|fenbufen 600 MG|1
Dosages|1337433|AEROSOL (GM)|AEROSOL (GM)|1
Dosages|1373854|BENZYL ALCOHOL 5 %|BENZYL ALCOHOL 5 %|1
Dosages|1374018|CAMPHOR 3.25 %|CAMPHOR 3.25 %|1
Dosages|1374281|CODEINE PHOSPHATE 1 MG|CODEINE PHOSPHATE 1 MG|1
Dosages|1375125|Iron 50 MG|Iron 50 MG|1
Dosages|1377405|VITAMIN B2 6 MG|VITAMIN B2 6 MG|1
Dosages|1379992|AMLODIPINE BESYLATE 10 MG|AMLODIPINE BESYLATE 10 MG|1
Dosages|1380339|MEPERIDINE HYDROCHLORIDE 10 MG/ML|MEPERIDINE HYDROCHLORIDE 10 MG/ML|1
Dosages|1381143|TRICHLOROACETIC ACID 25 %|TRICHLOROACETIC ACID 25 %|1
END RANDOM CASES

TOP 50 FREQUENT Short token:
Short token|332285|Of|Of|89239224
Short token|456628|oF|oF|89239224
Short token|1515981|And|And|59896788
Short token|1550557|and|and|59896788
Short token|1706368|And|And|59896788
Short token|2838|AND|AND|59885756
Short token|21201|IN|IN|54392482
Short token|21206|IN|IN|54392482
Short token|150312|In|In|54392482
Short token|332285|In|In|54392482
Short token|439204|in|in|54392482
Short token|1706540|To|To|31587690
Short token|40363|TO|TO|31581124
Short token|40407|TO|TO|31581124
Short token|1442985|a|a|30604554
Short token|1442986|A|A|30604554
Short token|1522424|A|A|30604554
Short token|1706280|a|a|30604554
Short token|1706281|A|A|30604554
Short token|1706282|A|A|30604554
Short token|227089|A|A|30598458
Short token|457243|A|A|30598458
Short token|1705690|With|With|21443934
Short token|332287|With|With|21439198
Short token|1421478|WAS|WAS|16240979
Short token|1562169|FOR|FOR|15698852
Short token|521125|For|For|15696045
Short token|6448|BY|BY|13089227
Short token|6539|BY|BY|13089227
Short token|1556119|Is|Is|11864452
Short token|20750|IS|IS|11861009
Short token|22271|IS|IS|11861009
Short token|441913|is|is|11861009
Short token|1720294|On|On|9527976
Short token|1517320|From|From|8348455
Short token|1314970|1|1|8132848
Short token|1513302|1|1|8132848
Short token|1579809|1|1|8132848
Short token|205447|1|1|8132251
Short token|227032|1|1|8132251
Short token|439078|-1|-1|8132251
Short token|470206|+1|+1|8132251
Short token|920321|1|1|8132251
Short token|1563293|As|As|7925031
Short token|1706103|As|As|7925031
Short token|3507|as|as|7923305
Short token|4340|AS|AS|7923305
Short token|242536|AS|AS|7923305
Short token|1080058|This|This|6882699
Short token|1314971|2|2|6808043
END TOP 50 FREQUENT

RANDOM CASES Short token:
Short token|1442986|A|A|30604554
Short token|6448|BY|BY|13089227

Short token|1706103|As|As|7925031
Short token|227038|5|5|4400832
Short token|439084|>5|>5|4400832
Short token|205103|Between|Between|3355470
Short token|439115|P|P|3349914
Short token|22277|IT|IT|2737985
Short token|340978|May|May|2641854
Short token|1298908|no|no|2584482
Short token|23449|ALL|ALL|2459490
Short token|1704852|CAN|CAN|1994383
Short token|227093|I|I|1864487
Short token|1524063|Use|Use|1546829
Short token|1551055|T|T|1403112
Short token|439111|H|H|1398028
Short token|1706495|L|L|1207374
Short token|1551054|S|S|1193810
Short token|470228|30|30|921338
Short token|439122|X|X|908326
Short token|750519|WHILE|WHILE|898063
Short token|1706457|m|m|871296
Short token|456533|M|M|871209
Short token|6368|100|100|765851
Short token|227046|11|11|763542
Short token|470218|14|14|742823
Short token|202194|K+|K+|531471
Short token|439112|K|K|531471
Short token|282416|Overall|Overall|404925
Short token|332183|Often|Often|355392
Short token|227062|23|23|342821
Short token|1720176|Upon|Upon|329740
Short token|227075|32|32|290000
Short token|450357|32|32|290000
Short token|470244|75|75|282975
Short token|1522726|IV|IV|263923
Short token|1519042|2-3|2-3|232127
Short token|439673|U|U|231751
Short token|439256|J|J|203370
Short token|1705108|Y|Y|168500
Short token|442759|3/6|3/6|86574
Short token|439141|q|q|84991
Short token|442749|6/5|6/5|53641
Short token|1732|004|004|29524
Short token|470355|7+7|7+7|29321
Short token|470254|112|112|26361
Short token|470256|126|126|23608
Short token|1520439|129|129|22336
Short token|470362|10+10|10+10|20879
Short token|470263|168|168|16483
Short token|15024|231|231|13750
Short token|442752|20/40|20/40|13008
Short token|442766|1/24|1/24|12171
Short token|13014|214|214|8873
Short token|1510707|A/J|A/J|8706
Short token|450362|380|380|8408
Short token|470369|25+2|25+2|7725
Short token|3023|024|024|6892

Short token|16132|246|246|6856
Short token|1549830|022|022|6298
Short token|12764|D.C.|D.C.|5948
Short token|456587|1958|1958|5899
Short token|28971|512|512|5348
Short token|282245|580|580|4254
Short token|439390|mL/s|mL/s|4035
Short token|22804|414|414|3782
Short token|22718|417|417|3557
Short token|23190|422|422|3504
Short token|442764|3/36|3/36|3252
Short token|4348|040|040|3135
Short token|24552|458|458|2943
Short token|37712|710|710|2506
Short token|442756|6/60|6/60|2338
Short token|35826|642|642|2201
Short token|28366|574|574|2097
Short token|450334|18/22|18/22|2080
Short token|31529|608|608|2067
Short token|439494|km/h|km/h|1975
Short token|30375|598|598|1966
Short token|30266|591|591|1959
Short token|32356|616|616|1917
Short token|36056|662|662|1729
Short token|1513911|N.D.|N.D.|1646
Short token|40363|768|768|1631
Short token|13715|818|818|1596
Short token|41405|796|796|1399
Short token|42469|862|862|1254
Short token|43402|887|887|1201
Short token|444810|B 24|B 24|365
Short token|442755|20/120|20/120|349
Short token|444968|II/X|II/X|347
Short token|1705073|I-309|I-309|252
Short token|719054|C-500|C-500|223
Short token|1515150|I 3.2|I 3.2|147
Short token|444973|IV/R|IV/R|114
Short token|1513008|M.P.H.|M.P.H.|100
Short token|7787|T.I.A.|T.I.A.|36
Short token|450326|1473 K|1473 K|14
Short token|1513007|M.H.S.A.|M.H.S.A.|7
END RANDOM CASES

TOP 50 FREQUENT At-sign:
No cases found
END TOP 50 FREQUENT

TOP 50 FREQUENT EC numbers:
EC numbers|31727|EC 2.7|EC 2.7|3397
EC numbers|85249|EC 2.4|EC 2.4|1845
EC numbers|108836|EC 2.7.1.-|EC 2.7.1.-|1622
EC numbers|108855|EC 2.7.1.-|EC 2.7.1.-|1622
EC numbers|217310|EC 2.7.1.-|EC 2.7.1.-|1622
EC numbers|258733|EC 2.7.1.-|EC 2.7.1.-|1622
EC numbers|259367|EC 2.7.1.-|EC 2.7.1.-|1622
EC numbers|659150|EC 2.7.1.-|EC 2.7.1.-|1622

EC numbers|673406|EC 2.7.1.-|EC 2.7.1.-|1622
EC numbers|1333180|EC 2.7.1.-|EC 2.7.1.-|1622
EC numbers|1333735|EC 2.7.1.-|EC 2.7.1.-|1622
EC numbers|1333738|EC 2.7.1.-|EC 2.7.1.-|1622
EC numbers|1337052|EC 2.7.1.-|EC 2.7.1.-|1622
EC numbers|1447440|EC 2.7.1.-|EC 2.7.1.-|1622
EC numbers|1390|EC 2.3|EC 2.3|1487
EC numbers|390904|EC 2.3.1.-|EC 2.3.1.-|1090
EC numbers|1259556|EC 3.6.1.-|EC 3.6.1.-|1010
EC numbers|2594|EC 2.6.1|EC 2.6.1|848
EC numbers|1313290|EC 3.4.24.-|EC 3.4.24.-|819
EC numbers|966207|EC 3.4.21.-|EC 3.4.21.-|747
EC numbers|1705965|EC 3.4.21.-|EC 3.4.21.-|747
EC numbers|28632|EC 2.7.7|EC 2.7.7|702
EC numbers|25831|EC 2.1.1|EC 2.1.1|621
EC numbers|22917|EC 1.1.1.27|EC 1.1.1.27|455
EC numbers|1332236|EC 2.5|EC 2.5|442
EC numbers|291573|EC 3.4.22.-|EC 3.4.22.-|410
EC numbers|537969|EC 3.4.22.-|EC 3.4.22.-|410
EC numbers|538091|EC 3.4.22.-|EC 3.4.22.-|410
EC numbers|758959|EC 3.4.22.-|EC 3.4.22.-|410
EC numbers|769345|EC 3.4.22.-|EC 3.4.22.-|410
EC numbers|910167|EC 3.4.22.-|EC 3.4.22.-|410
EC numbers|912403|EC 3.4.22.-|EC 3.4.22.-|410
EC numbers|1437927|EC 3.4.22.-|EC 3.4.22.-|410
EC numbers|1453171|EC 3.4.22.-|EC 3.4.22.-|410
EC numbers|10287|EC 2.7.3.2|EC 2.7.3.2|360
EC numbers|17757|EC 1.1.1.49|EC 1.1.1.49|293
EC numbers|1382029|EC 4.6.1.1|EC 4.6.1.1|285
EC numbers|17837|EC 2.5.1.18|EC 2.5.1.18|243
EC numbers|19475|EC 2.7.1.1|EC 2.7.1.1|224
EC numbers|33640|EC 2.7.1.37|EC 2.7.1.37|210
EC numbers|72402|EC 2.7.1.37|EC 2.7.1.37|210
EC numbers|244987|EC 2.7.1.37|EC 2.7.1.37|210
EC numbers|294209|EC 2.7.1.37|EC 2.7.1.37|210
EC numbers|380146|EC 2.7.1.37|EC 2.7.1.37|210
EC numbers|541150|EC 2.7.1.37|EC 2.7.1.37|210
EC numbers|1314894|EC 2.7.1.37|EC 2.7.1.37|210
EC numbers|1332856|EC 2.7.1.37|EC 2.7.1.37|210
EC numbers|1447968|EC 2.7.1.37|EC 2.7.1.37|210
EC numbers|31667|EC 3.1.1.4|EC 3.1.1.4|189
EC numbers|35681|EC 2.7.7.6|EC 2.7.7.6|185
END TOP 50 FREQUENT

RANDOM CASES EC numbers:
EC numbers|85249|EC 2.4|EC 2.4|1845
EC numbers|108836|EC 2.7.1.-|EC 2.7.1.-|1622
EC numbers|108855|EC 2.7.1.-|EC 2.7.1.-|1622
EC numbers|217310|EC 2.7.1.-|EC 2.7.1.-|1622
EC numbers|258733|EC 2.7.1.-|EC 2.7.1.-|1622
EC numbers|659150|EC 2.7.1.-|EC 2.7.1.-|1622
EC numbers|1333735|EC 2.7.1.-|EC 2.7.1.-|1622
EC numbers|1337052|EC 2.7.1.-|EC 2.7.1.-|1622
EC numbers|1447440|EC 2.7.1.-|EC 2.7.1.-|1622
EC numbers|1390|EC 2.3|EC 2.3|1487
EC numbers|2594|EC 2.6.1|EC 2.6.1|848

EC numbers|1313290|EC 3.4.24.-|EC 3.4.24.-|819
EC numbers|966207|EC 3.4.21.-|EC 3.4.21.-|747
EC numbers|1332236|EC 2.5|EC 2.5|442
EC numbers|537969|EC 3.4.22.-|EC 3.4.22.-|410
EC numbers|538091|EC 3.4.22.-|EC 3.4.22.-|410
EC numbers|758959|EC 3.4.22.-|EC 3.4.22.-|410
EC numbers|910167|EC 3.4.22.-|EC 3.4.22.-|410
EC numbers|1453171|EC 3.4.22.-|EC 3.4.22.-|410
EC numbers|17757|EC 1.1.1.49|EC 1.1.1.49|293
EC numbers|1382029|EC 4.6.1.1|EC 4.6.1.1|285
EC numbers|17837|EC 2.5.1.18|EC 2.5.1.18|243
EC numbers|19475|EC 2.7.1.1|EC 2.7.1.1|224
EC numbers|72402|EC 2.7.1.37|EC 2.7.1.37|210
EC numbers|244987|EC 2.7.1.37|EC 2.7.1.37|210
EC numbers|541150|EC 2.7.1.37|EC 2.7.1.37|210
EC numbers|1332856|EC 2.7.1.37|EC 2.7.1.37|210
EC numbers|1447968|EC 2.7.1.37|EC 2.7.1.37|210
EC numbers|35681|EC 2.7.7.6|EC 2.7.7.6|185
EC numbers|20687|EC 2.4.2.8|EC 2.4.2.8|178
EC numbers|752312|EC 2.7.2-|EC 2.7.2-|176
EC numbers|1457|EC 3.5.4.4|EC 3.5.4.4|155
EC numbers|12892|EC 2.7.7.7|EC 2.7.7.7|119
EC numbers|40078|EC 2.7.1.21|EC 2.7.1.21|112
EC numbers|27310|EC 1.6.2.4|EC 1.6.2.4|104
EC numbers|85144|EC 5.3.1.9|EC 5.3.1.9|102
EC numbers|1261253|EC 2.3.1.5|EC 2.3.1.5|92
EC numbers|34135|EC 2.4.2.1|EC 2.4.2.1|91
EC numbers|1414|EC 2.4.2.7|EC 2.4.2.7|66
EC numbers|3805|EC 1.14.14.1|EC 1.14.14.1|61
EC numbers|57223|EC 1.14.14.1|EC 1.14.14.1|61
EC numbers|59735|EC 1.14.14.1|EC 1.14.14.1|61
EC numbers|207509|EC 1.14.14.1|EC 1.14.14.1|61
EC numbers|608437|EC 1.14.14.1|EC 1.14.14.1|61
EC numbers|960580|EC 1.14.14.1|EC 1.14.14.1|61
EC numbers|1142644|EC 1.14.14.1|EC 1.14.14.1|61
EC numbers|1321893|EC 1.14.14.1|EC 1.14.14.1|61
EC numbers|7407|EC 2.1.1.6|EC 2.1.1.6|57
EC numbers|132555|EC 1.14.13.39|EC 1.14.13.39|56
EC numbers|297817|EC 1.17.4.1|EC 1.17.4.1|50
EC numbers|249196|EC 3.1.3.48|EC 3.1.3.48|48
EC numbers|287531|EC 3.1.3.48|EC 3.1.3.48|48
EC numbers|1333317|EC 3.1.3.48|EC 3.1.3.48|48
EC numbers|2492|EC 2.4.2.14|EC 2.4.2.14|45
EC numbers|26027|EC 3.3.2.3|EC 3.3.2.3|45
EC numbers|249196|EC 3.1.3.16|EC 3.1.3.16|45
EC numbers|1333317|EC 3.1.3.16|EC 3.1.3.16|45
EC numbers|3993|EC 3.5.1.1|EC 3.5.1.1|44
EC numbers|17796|EC 3.5.1.2|EC 3.5.1.2|42
EC numbers|52451|EC 3.1.1.2|EC 3.1.1.2|39
EC numbers|68895|EC 2.7.4.6|EC 2.7.4.6|39
EC numbers|8169|EC 2.3.1.28|EC 2.3.1.28|35
EC numbers|12881|EC 2.7.7.31|EC 2.7.7.31|29
EC numbers|15683|EC 2.3.1.85|EC 2.3.1.85|29
EC numbers|17037|EC 6.3.2.2|EC 6.3.2.2|28
EC numbers|11486|EC 2.7.1.74|EC 2.7.1.74|27
EC numbers|8415|EC 2.7.1.32|EC 2.7.1.32|26

EC numbers|58126|EC 1.3.1.2|EC 1.3.1.2|24
EC numbers|1309483|EC 3.1.26.-|EC 3.1.26.-|21
EC numbers|33681|EC 2.7.1.112|EC 2.7.1.112|19
EC numbers|109317|EC 2.7.1.112|EC 2.7.1.112|19
EC numbers|117718|EC 2.7.1.112|EC 2.7.1.112|19
EC numbers|138965|EC 2.7.1.112|EC 2.7.1.112|19
EC numbers|169658|EC 2.7.1.112|EC 2.7.1.112|19
EC numbers|169661|EC 2.7.1.112|EC 2.7.1.112|19
EC numbers|290067|EC 2.7.1.112|EC 2.7.1.112|19
EC numbers|290068|EC 2.7.1.112|EC 2.7.1.112|19
EC numbers|907648|EC 2.7.1.112|EC 2.7.1.112|19
EC numbers|915156|EC 2.7.1.112|EC 2.7.1.112|19
EC numbers|1259418|EC 2.7.1.112|EC 2.7.1.112|19
EC numbers|1333409|EC 2.7.1.112|EC 2.7.1.112|19
EC numbers|1333410|EC 2.7.1.112|EC 2.7.1.112|19
EC numbers|1370509|EC 2.7.1.112|EC 2.7.1.112|19
EC numbers|1504624|EC 2.7.1.112|EC 2.7.1.112|19
EC numbers|24375|EC 1.4.3.13|EC 1.4.3.13|17
EC numbers|54871|EC 3.4.22.15|EC 3.4.22.15|17
EC numbers|1452577|EC 2.1.1.37|EC 2.1.1.37|15
EC numbers|35379|EC 2.7.7.49|EC 2.7.7.49|14
EC numbers|1334066|EC 1.1.1.51|EC 1.1.1.51|13
EC numbers|1334858|EC 2.3.1.97|EC 2.3.1.97|13
EC numbers|1442799|EC 6.5.1.1|EC 6.5.1.1|13
EC numbers|1336737|EC 2.1.1.67|EC 2.1.1.67|10
EC numbers|54869|EC 3.4.22.16|EC 3.4.22.16|9
EC numbers|66357|EC 1.5.1.20|EC 1.5.1.20|8
EC numbers|35685|EC 2.7.7.48|EC 2.7.7.48|7
EC numbers|41497|EC 6.1.1.1|EC 6.1.1.1|7
EC numbers|299856|EC 2.7.1.3|EC 2.7.1.3|6
EC numbers|62773|EC 2.3.1.48|EC 2.3.1.48|5
EC numbers|12873|EC 2.1.1.113|EC 2.1.1.113|1
END RANDOM CASES

TOP 50 FREQUENT Any class.:
Any class.|205426|Unclassified|Unclassified|3399
Any class.|1650340|unclassified.|unclassified.|3399
Any class.|584609|Unclassified cells|Unclassified cells|28
Any class.|42566|Unclassified virus|Unclassified virus|16
Any class.|1336853|Unclassified Renal Cell Carcinoma|Unclassified Renal Cell Carcinoma|14
Any class.|42566|UNCLASSIFIED VIRUSES|UNCLASSIFIED VIRUSES|11
Any class.|42566|Unclassified Viruses|Unclassified Viruses|11
Any class.|318309|unclassified Bacteria|unclassified Bacteria|9
Any class.|318309|Unclassified bacterium|Unclassified bacterium|9
Any class.|1282956|Unclassified sprue|Unclassified sprue|9
Any class.|1335558|Unclassified Protein|Unclassified Protein|6
Any class.|33976|PSYCHOTIC DISORDERS NOT ELSEWHERE CLASSIFIED|PSYCHOTIC DISORDERS NOT ELSEWHERE CLASSIFIED|5
Any class.|36654|Senility without mention of psychosis|Senility without mention of psychosis|5
Any class.|868741|Impulse-Control Disorders Not Elsewhere Classified|Impulse-Control Disorders Not Elsewhere Classified|5
Any class.|868759|PSYCHOTIC DISORDERS NOT ELSEWHERE CLASSIFIED|PSYCHOTIC DISORDERS NOT ELSEWHERE CLASSIFIED|5
Any class.|1040101|unclassified Enteroviruses|unclassified Enteroviruses|4
Any class.|1650340|unclassified sequences|unclassified sequences|4
Any class.|887854|unclassified Microsporidia|unclassified Microsporidia|3

Any class.|302378|Chronic airway obstruction, not elsewhere classified|Chronic airway obstruction, not elsewhere classified|2
Any class.|495356|Disorders related to short gestation and low birth weight, not elsewhere classified|Disorders related to short gestation and low birth weight, not elsewhere classified|2
Any class.|1000354|unclassified phages|unclassified phages|2
Any class.|1218034|unclassified Acinetobacter|unclassified Acinetobacter|2
Any class.|1483598|unclassified Theileria|unclassified Theileria|2
Any class.|1515286|Testicular Intratubular Germ Cell Neoplasia, Unclassified|Testicular Intratubular Germ Cell Neoplasia, Unclassified|2
Any class.|42566|Viruses, Unclassified|Viruses, Unclassified|1
Any class.|270826|Unclassified epileptic seizures|Unclassified epileptic seizures|1
Any class.|302370|Special symptoms or syndromes, not elsewhere classified|Special symptoms or syndromes, not elsewhere classified|1
Any class.|318309|unclassified Eubacteria|unclassified Eubacteria|1
Any class.|320212|Unclassified fungus|Unclassified fungus|1
Any class.|392650|Measles without mention of complication|Measles without mention of complication|1
Any class.|694459|Symptoms, signs and abnormal clinical and laboratory findings, not elsewhere classified|Symptoms, signs and abnormal clinical and laboratory findings, not elsewhere classified|1
Any class.|1004860|unclassified Cyanobacteria|unclassified Cyanobacteria|1
Any class.|1008373|unclassified Flavivirus|unclassified Flavivirus|1
Any class.|1026612|unclassified Pasteurellaceae|unclassified Pasteurellaceae|1
Any class.|1037534|unclassified phytoplasmas|unclassified phytoplasmas|1
Any class.|1057061|unclassified Betaproteobacteria|unclassified Betaproteobacteria|1
Any class.|1093811|unclassified Pestivirus|unclassified Pestivirus|1
Any class.|1209842|unclassified Aquareovirus|unclassified Aquareovirus|1
Any class.|1225689|unclassified Ralstonia|unclassified Ralstonia|1
Any class.|1460830|unclassified Rickettsiales|unclassified Rickettsiales|1
Any class.|1621543|unclassified Bacilli|unclassified Bacilli|1
Any class.|1637420|unclassified Lyssavirus|unclassified Lyssavirus|1
END TOP 50 FREQUENT

TOP 50 FREQUENT Any underspec.:
Any underspec.|205370|Unspecified|Unspecified|2822
Any underspec.|1549663|Unspecified|Unspecified|2822
Any underspec.|1518425|Not Otherwise Specified|Not Otherwise Specified|1385
Any underspec.|205370|Not Specified|Not Specified|536
Any underspec.|1524083|Not Specified|Not Specified|536
Any underspec.|6104|Brain, NOS|Brain, NOS|141
Any underspec.|79774|Peripheral T-cell lymphoma unspecified|Peripheral T-cell lymphoma unspecified|113
Any underspec.|12634|Disorder, NOS|Disorder, NOS|64
Any underspec.|7097|Carcinoma, NOS|Carcinoma, NOS|42
Any underspec.|7634|Cell, NOS|Cell, NOS|37
Any underspec.|14442|Enzyme, NOS|Enzyme, NOS|28
Any underspec.|26845|Muscle, NOS|Muscle, NOS|26
Any underspec.|1721|Affect, NOS|Affect, NOS|22
Any underspec.|33975|Psychosis, NOS|Psychosis, NOS|22
Any underspec.|1519812|Unspecified Site|Unspecified Site|21
Any underspec.|13473|Eating disorder, NOS|Eating disorder, NOS|20
Any underspec.|17337|Gene, NOS|Gene, NOS|20
Any underspec.|1261473|Sarcoma, NOS|Sarcoma, NOS|20
Any underspec.|1522022|Sarcoma, NOS|Sarcoma, NOS|20
Any underspec.|24109|Lung, NOS|Lung, NOS|19
Any underspec.|847477|Unspecified neoplasms|Unspecified neoplasms|16
Any underspec.|1622815|Unspecified Peripheral T-Cell Lymphoma|Unspecified Peripheral T-Cell Lymphoma|16
Any underspec.|11570|Depression, NOS|Depression, NOS|15
Any underspec.|27651|Tumor, NOS|Tumor, NOS|15

Any underspec.|8633|Chromosome, NOS|Chromosome, NOS|14
Any underspec.|40300|Tissue, NOS|Tissue, NOS|12
Any underspec.|18563|Hand, NOS|Hand, NOS|11
Any underspec.|23884|Liver, NOS|Liver, NOS|11
Any underspec.|33684|Protein, NOS|Protein, NOS|11
Any underspec.|185117|Expression, NOS|Expression, NOS|11
Any underspec.|34693|Rat, NOS|Rat, NOS|10
Any underspec.|42149|Uterus, NOS|Uterus, NOS|10
Any underspec.|438984|Unspecified conditions|Unspecified conditions|10
Any underspec.|18681|Headache, NOS|Headache, NOS|9
Any underspec.|25362|Unspecified mental retardation|Unspecified mental retardation|9
Any underspec.|28778|Block, NOS|Block, NOS|9
Any underspec.|6826|CANCER (NOS)|CANCER (NOS)|8
Any underspec.|24201|Lymnaea, NOS|Lymnaea, NOS|8
Any underspec.|30193|Unspecified Pain|Unspecified Pain|8
Any underspec.|30193|Unspecified pain|Unspecified pain|8
Any underspec.|497327|Unspecified dementia|Unspecified dementia|8
Any underspec.|3842|Artery, NOS|Artery, NOS|7
Any underspec.|33975|Unspecified psychosis|Unspecified psychosis|7
Any underspec.|1134719|Invasive Ductal Carcinoma, Not Otherwise Specified|Invasive Ductal Carcinoma, Not Otherwise Specified|7
Any underspec.|1285092|Gland, NOS|Gland, NOS|7
Any underspec.|1518740|Ovarian Steroid Cell Tumor, NOS|Ovarian Steroid Cell Tumor, NOS|7
Any underspec.|4114|Astrocytoma, NOS|Astrocytoma, NOS|6
Any underspec.|23418|Unspecified leukemia|Unspecified leukemia|6
Any underspec.|347947|Debility, unspecified|Debility, unspecified|6
Any underspec.|1657111|unspecified disease|unspecified disease|6
END TOP 50 FREQUENT

RANDOM CASES Any underspec.:
Any underspec.|205370|Unspecified|Unspecified|2822
Any underspec.|6104|Brain, NOS|Brain, NOS|141
Any underspec.|79774|Peripheral T-cell lymphoma unspecified|Peripheral T-cell lymphoma unspecified|113
Any underspec.|12634|Disorder, NOS|Disorder, NOS|64
Any underspec.|1721|Affect, NOS|Affect, NOS|22
Any underspec.|24109|Lung, NOS|Lung, NOS|19
Any underspec.|11570|Depression, NOS|Depression, NOS|15
Any underspec.|27651|Tumor, NOS|Tumor, NOS|15
Any underspec.|8633|Chromosome, NOS|Chromosome, NOS|14
Any underspec.|40300|Tissue, NOS|Tissue, NOS|12
Any underspec.|18563|Hand, NOS|Hand, NOS|11
Any underspec.|185117|Expression, NOS|Expression, NOS|11
Any underspec.|28778|Block, NOS|Block, NOS|9
Any underspec.|30193|Unspecified Pain|Unspecified Pain|8
Any underspec.|497327|Unspecified dementia|Unspecified dementia|8
Any underspec.|3842|Artery, NOS|Artery, NOS|7
Any underspec.|1285092|Gland, NOS|Gland, NOS|7
Any underspec.|924|Unspecified accidents|Unspecified accidents|5
Any underspec.|8073|Developmental disorder, NOS|Developmental disorder, NOS|5
Any underspec.|37925|Spinal cord, NOS|Spinal cord, NOS|5
Any underspec.|175677|Injury, unspecified|Injury, unspecified|5
Any underspec.|1267092|Smooth muscle, NOS|Smooth muscle, NOS|5
Any underspec.|3469|Anxiety disorder, NOS|Anxiety disorder, NOS|4
Any underspec.|20517|Allergy, unspecified|Allergy, unspecified|4
Any underspec.|24299|Lymphoma, NOS|Lymphoma, NOS|4
Any underspec.|330390|Beta, NOS|Beta, NOS|4

Any underspec.|3483|Aorta, NOS|Aorta, NOS|3
Any underspec.|18787|Heart, NOS|Heart, NOS|3
Any underspec.|27121|Unspecified myositis|Unspecified myositis|3
Any underspec.|36690|Unspecified septicemia|Unspecified septicemia|3
Any underspec.|37633|Solution, NOS|Solution, NOS|3
Any underspec.|158892|Disorders relating to short gestation and unspecified low birthweight|Disorders relating to short gestation and unspecified low birthweight|3
Any underspec.|458827|Airway, NOS|Airway, NOS|3
Any underspec.|17612|Unspecified open-angle glaucoma|Unspecified open-angle glaucoma|2
Any underspec.|17638|Glioma, NOS|Glioma, NOS|2
Any underspec.|20507|Hyperplasia, NOS|Hyperplasia, NOS|2
Any underspec.|23418|Unspecified Leukemias|Unspecified Leukemias|2
Any underspec.|27651|Neoplasm, NOS|Neoplasm, NOS|2
Any underspec.|29045|Oocyte, NOS|Oocyte, NOS|2
Any underspec.|30920|Unspecified peptic ulcer|Unspecified peptic ulcer|2
Any underspec.|42027|Urinary tract, NOS|Urinary tract, NOS|2
Any underspec.|85639|Unspecified fall|Unspecified fall|2
Any underspec.|85979|Guinea pig, NOS|Guinea pig, NOS|2
Any underspec.|162337|Vascular permeability, NOS|Vascular permeability, NOS|2
Any underspec.|441800|Grade, NOS|Grade, NOS|2
Any underspec.|941062|Unspecified colitis|Unspecified colitis|2
Any underspec.|1134719|Invasive Ductal Carcinoma, NOS|Invasive Ductal Carcinoma, NOS|2
Any underspec.|1830|Agrobacterium, NOS|Agrobacterium, NOS|1
Any underspec.|2874|Aplastic anaemia, unspecified|Aplastic anaemia, unspecified|1
Any underspec.|5456|Binding site, NOS|Binding site, NOS|1
Any underspec.|5612|Birth weight, NOS|Birth weight, NOS|1
Any underspec.|6277|Bronchitis, NOS|Bronchitis, NOS|1
Any underspec.|6277|Bronchitis unspecified|Bronchitis unspecified|1
Any underspec.|7009|Carbon, NOS|Carbon, NOS|1
Any underspec.|7276|Internal carotid artery, NOS|Internal carotid artery, NOS|1
Any underspec.|7610|Nucleus, NOS|Nucleus, NOS|1
Any underspec.|7785|Cerebral infarction, unspecified|Cerebral infarction, unspecified|1
Any underspec.|7820|Unspecified cerebrovascular disease|Unspecified cerebrovascular disease|1
Any underspec.|7820|Cerebrovascular disease, unspecified|Cerebrovascular disease, unspecified|1
Any underspec.|13227|Medicine, NOS|Medicine, NOS|1
Any underspec.|13604|Edema, unspecified|Edema, unspecified|1
Any underspec.|17601|Glaucoma, NOS|Glaucoma, NOS|1
Any underspec.|18524|Hallucinations, unspecified|Hallucinations, unspecified|1
Any underspec.|18801|Heart failure, unspecified|Heart failure, unspecified|1
Any underspec.|20614|Hypoglossal nerve, NOS|Hypoglossal nerve, NOS|1
Any underspec.|20885|Ileum, NOS|Ileum, NOS|1
Any underspec.|21469|Inhibition, NOS|Inhibition, NOS|1
Any underspec.|23418|Leukemia, unspecified|Leukemia, unspecified|1
Any underspec.|23916|Lizard, NOS|Lizard, NOS|1
Any underspec.|26473|Monocyte, NOS|Monocyte, NOS|1
Any underspec.|26771|Unspecified multiple injuries|Unspecified multiple injuries|1
Any underspec.|29227|Organic mental disorder, NOS|Organic mental disorder, NOS|1
Any underspec.|31154|Unspecified peritonitis|Unspecified peritonitis|1
Any underspec.|32043|Placenta, NOS|Placenta, NOS|1
Any underspec.|33325|Prognosis, NOS|Prognosis, NOS|1
Any underspec.|36349|Paranoid schizophrenia, NOS|Paranoid schizophrenia, NOS|1
Any underspec.|37199|Unspecified sinusitis|Unspecified sinusitis|1
Any underspec.|37949|Spine, NOS|Spine, NOS|1
Any underspec.|39597|Testis, NOS|Testis, NOS|1
Any underspec.|41880|Unspecified reason for consultation|Unspecified reason for consultation|1
Any underspec.|42029|Urinary tract infection, site not specified|Urinary tract infection, site not specified|1

Any underspec.|42360|Vas deferens, NOS|Vas deferens, NOS|1
Any underspec.|42401|Vasodilatation, NOS|Vasodilatation, NOS|1
Any underspec.|79189|Cytokine, NOS|Cytokine, NOS|1
Any underspec.|178282|Unspecified abdominal hernia|Unspecified abdominal hernia|1
Any underspec.|185023|Fixation, NOS|Fixation, NOS|1
Any underspec.|222041|Shell, NOS|Shell, NOS|1
Any underspec.|242724|Leaves, NOS|Leaves, NOS|1
Any underspec.|274281|Exposure, NOS|Exposure, NOS|1
Any underspec.|332464|Widening, NOS|Widening, NOS|1
Any underspec.|334044|Dysplasia, NOS|Dysplasia, NOS|1
Any underspec.|336769|Lighter, NOS|Lighter, NOS|1
Any underspec.|442874|NEUROPATHY - (NOS)|NEUROPATHY - (NOS)|1
Any underspec.|497126|GENERAL AND UNSPECIFIED|GENERAL AND UNSPECIFIED|1
Any underspec.|524528|Pervasive developmental disorder, unspecified|Pervasive developmental disorder, unspecified|1
Any underspec.|543467|Operation, NOS|Operation, NOS|1
Any underspec.|687028|Duct, NOS|Duct, NOS|1
Any underspec.|1123019|Ovine, NOS|Ovine, NOS|1
Any underspec.|1258666|Ganglion, NOS|Ganglion, NOS|1
Any underspec.|1442981|Alcoholic liver damage unspecified|Alcoholic liver damage unspecified|1
END RANDOM CASES

TOP 50 FREQUENT Misc.:
Misc.|1271040|Other|Other|2068321
Misc.|1521979|OTHER|OTHER|2068321
Misc.|1546380|Other|Other|2068321
Misc.|1546725|Other|Other|2068321
Misc.|1546836|Other|Other|2068321
Misc.|1546840|Other|Other|2068321
Misc.|1546902|Other|Other|2068321
Misc.|1546930|Other|Other|2068321
Misc.|1547110|Other|Other|2068321
Misc.|1547196|Other|Other|2068321
Misc.|1547233|Other|Other|2068321
Misc.|1547241|Other|Other|2068321
Misc.|1547267|Other|Other|2068321
Misc.|1547272|Other|Other|2068321
Misc.|1547281|Other|Other|2068321
Misc.|1547292|Other|Other|2068321
Misc.|1547304|Other|Other|2068321
Misc.|1547309|Other|Other|2068321
Misc.|1547994|Other|Other|2068321
Misc.|1549063|Other|Other|2068321
Misc.|1549104|Other|Other|2068321
Misc.|1549110|Other|Other|2068321
Misc.|1550146|Other|Other|2068321
Misc.|1556042|Other|Other|2068321
Misc.|1556043|Other|Other|2068321
Misc.|1556044|other|other|2068321
Misc.|1556045|Other|Other|2068321
Misc.|1556046|Other|Other|2068321
Misc.|1556048|Other|Other|2068321
Misc.|1556049|Other|Other|2068321
Misc.|1556050|Other|Other|2068321
Misc.|1556051|Other|Other|2068321
Misc.|1556052|Other|Other|2068321
Misc.|1556053|Other|Other|2068321

Misc.|1556054|Other|Other|2068321
Misc.|1556055|Other|Other|2068321
Misc.|1556056|Other|Other|2068321
Misc.|1556057|Other|Other|2068321
Misc.|1561608|Other|Other|2068321
Misc.|205394|Other|Other|2067762
Misc.|220886|Other|Other|2067762
Misc.|1521803|UNKNOWN|UNKNOWN|174793
Misc.|1546837|Unknown|Unknown|174793
Misc.|1546841|Unknown|Unknown|174793
Misc.|1547283|Unknown|Unknown|174793
Misc.|1547294|Unknown|Unknown|174793
Misc.|1547306|Unknown|Unknown|174793
Misc.|1547312|Unknown|Unknown|174793
Misc.|1548340|Unknown|Unknown|174793
Misc.|1548502|Unknown|Unknown|174793
END TOP 50 FREQUENT

RANDOM CASES Misc.:
Misc.|1521979|OTHER|OTHER|2068321
Misc.|1556054|Other|Other|2068321
Misc.|1546837|Unknown|Unknown|174793
Misc.|1547294|Unknown|Unknown|174793
Misc.|1547306|Unknown|Unknown|174793
Misc.|1549064|Unknown|Unknown|174793
Misc.|1556120|Unknown|Unknown|174793
Misc.|1556134|Unknown|Unknown|174793
Misc.|439673|Unknown|Unknown|174742
Misc.|746919|NO TREATMENT|NO TREATMENT|14378
Misc.|1610167|Obsolete|Obsolete|1692
Misc.|1718172|OTHER INDICATORS|OTHER INDICATORS|868
Misc.|424577|No complaints|No complaints|663
Misc.|349232|Other anxiety disorders|Other anxiety disorders|420
Misc.|1557623|OTHER PAIN|OTHER PAIN|383
Misc.|220647|Carcinoma of unknown primary|Carcinoma of unknown primary|316
Misc.|1325814|other collagen|other collagen|303
Misc.|1550584|no shock|no shock|261
Misc.|973669|Other Vitamins|Other Vitamins|209
Misc.|28128|NO - Nitric oxide|NO - Nitric oxide|152
Misc.|425443|No respiratory symptoms|No respiratory symptoms|148
Misc.|236993|Other sleep disorders|Other sleep disorders|120
Misc.|850560|other autoimmune disease|other autoimmune disease|88
Misc.|813121|Other Medical Services|Other Medical Services|79
Misc.|29582|Other respiratory disorders|Other respiratory disorders|62
Misc.|156723|Other pregnancy complications|Other pregnancy complications|55
Misc.|178273|Other forms of heart disease|Other forms of heart disease|50
Misc.|496674|Other medical care|Other medical care|50
Misc.|425518|No sputum|No sputum|44
Misc.|1265234|Mycobacteria other than tubercle bacilli|Mycobacteria other than tubercle bacilli|44
Misc.|356919|Other antineoplastic drugs|Other antineoplastic drugs|41
Misc.|29704|Other perfusion|Other perfusion|36
Misc.|1335151|Other Finding|Other Finding|35
Misc.|695257|Other streptococcus|Other streptococcus|28
Misc.|236993|Other sleep disturbances|Other sleep disturbances|20
Misc.|585135|Metastatic adenocarcinoma of unknown origin|Metastatic adenocarcinoma of unknown origin|20
Misc.|319059|Other bunyaviruses|Other bunyaviruses|17

Misc.|682323|spouse or significant other|spouse or significant other|14
Misc.|416980|Other falls|Other falls|12
Misc.|1260416|Other forms of asthma|Other forms of asthma|12
Misc.|29485|Other acne|Other acne|11
Misc.|494846|Other dermatitis|Other dermatitis|11
Misc.|132701|No 233|No 233|10
Misc.|393949|Other cerebrovascular disease|Other cerebrovascular disease|10
Misc.|1709302|Obsolete Concept|Obsolete Concept|10
Misc.|1516648|Clinical Trials, Other|Clinical Trials, Other|9
Misc.|3232|MISCELLANEOUS ANTIBIOTICS|MISCELLANEOUS ANTIBIOTICS|8
Misc.|332126|No procedure for|No procedure for|8
Misc.|332442|No diagnostic abnormality|No diagnostic abnormality|8
Misc.|336404|Other construction workers|Other construction workers|8
Misc.|410004|Other fibromatoses|Other fibromatoses|7
Misc.|1442639|Other specialty services|Other specialty services|7
Misc.|1550138|Other Warning|Other Warning|7
Misc.|153325|Other infectious and parasitic diseases|Other infectious and parasitic diseases|6
Misc.|436087|Other multiple injuries|Other multiple injuries|6
Misc.|476291|Other digestive symptoms|Other digestive symptoms|6
Misc.|153322|Other infestations|Other infestations|5
Misc.|171957|NO 0756|NO 0756|5
Misc.|260661|Other eye problems|Other eye problems|5
Misc.|1718178|OTHER QUANTITATION|OTHER QUANTITATION|5
Misc.|29588|Other disorders of impulse control|Other disorders of impulse control|4
Misc.|494230|Other hereditary hemolytic anemias|Other hereditary hemolytic anemias|4
Misc.|497304|Neurological disease other|Neurological disease other|4
Misc.|564787|Rheumatoid arthritis - other joint|Rheumatoid arthritis - other joint|4
Misc.|1556327|Coagulation - Other|Coagulation - Other|3
Misc.|29582|Other respiratory system diseases|Other respiratory system diseases|2
Misc.|236870|No diagnosis on Axis I|No diagnosis on Axis I|2
Misc.|260663|Other ear problems|Other ear problems|2
Misc.|334727|Other physical scientists|Other physical scientists|2
Misc.|375193|Other conduct disorders|Other conduct disorders|2
Misc.|375697|Other anaphylactic shock|Other anaphylactic shock|2
Misc.|436071|Other elbow injuries|Other elbow injuries|2
Misc.|457748|No tooth decay|No tooth decay|2
Misc.|544625|Aspiration, other|Aspiration, other|2
Misc.|1513382|Molecular Biology, Other|Molecular Biology, Other|2
Misc.|11157|Deficiency of other vitamins|Deficiency of other vitamins|1
Misc.|29533|Other causes of encephalitis|Other causes of encephalitis|1
Misc.|29651|Other kidney transplantation|Other kidney transplantation|1
Misc.|29708|Other phototherapy|Other phototherapy|1
Misc.|33684|Protein (Obsolete)|Protein (Obsolete)|1
Misc.|153116|Other typhus|Other typhus|1
Misc.|156402|Other specified symptoms associated with female genital organs|Other specified symptoms associated with female genital organs|1
Misc.|220647|METASTATIC CARCINOMA WITH UNKNOWN PRIMARY|METASTATIC CARCINOMA WITH UNKNOWN PRIMARY|1
Misc.|237098|Other Environmental problem|Other Environmental problem|1
Misc.|276066|MISCELLANEOUS BACTERIAL INFECTIONS|MISCELLANEOUS BACTERIAL INFECTIONS|1
Misc.|348526|Other forms of keratitis|Other forms of keratitis|1
Misc.|349239|Other obsessive-compulsive disorders|Other obsessive-compulsive disorders|1
Misc.|423302|No pupil reaction|No pupil reaction|1
Misc.|425813|No nipple discharge|No nipple discharge|1
Misc.|473950|Other anesthetic (procedure)|Other anesthetic (procedure)|1
Misc.|477145|Other land transport accidents|Other land transport accidents|1

Misc.|477709|Other biomechanical lesions|Other biomechanical lesions|1
Misc.|490009|Other pyoderma|Other pyoderma|1
Misc.|520806|Sudden death, cause unknown|Sudden death, cause unknown|1
Misc.|566967|No vaginal blood loss|No vaginal blood loss|1
Misc.|585089|No endocervical cells present|No endocervical cells present|1
Misc.|732519|Other aerosol delivery systems|Other aerosol delivery systems|1
Misc.|809978|Gout and other crystal arthropathies|Gout and other crystal arthropathies|1
Misc.|1273800|Other related persons|Other related persons|1
Misc.|1561381|Neurology - Other|Neurology - Other|1
END RANDOM CASES

TOP 50 FREQUENT Words > 5:
Words > 5|19704|human immunodeficiency virus type-1 HIV-1|human immunodeficiency virus type-1 HIV-1|10215
Words > 5|7670|Centers for Disease Control and Prevention|Centers for Disease Control and Prevention|5194
Words > 5|1547112|Single photon emission computed tomography (SPECT)|Single photon emission computed tomography (SPECT)|4215
Words > 5|1168401|Carcinoma of the Head and Neck|Carcinoma of the Head and Neck|3698
Words > 5|206679|herpes simplex virus type-1 HSV-1|herpes simplex virus type-1 HSV-1|3473
Words > 5|1168401|Squamous Cell Carcinoma of the Head and Neck|Squamous Cell Carcinoma of the Head and Neck|3008
Words > 5|1168401|Head and Neck Squamous Cell Carcinoma|Head and Neck Squamous Cell Carcinoma|2860
Words > 5|376344|National Health and Nutrition Examination Survey|National Health and Nutrition Examination Survey|2818
Words > 5|1518101|Matrix-Assisted Laser Desorption/Ionization Time-of-Flight Mass Spectrometry|Matrix-Assisted Laser Desorption/Ionization Time-of-Flight Mass Spectrometry|2497
Words > 5|1136324|Diagnostic and Statistical Manual of Mental Disorders|Diagnostic and Statistical Manual of Mental Disorders|2383
Words > 5|1151233|3-hydroxy-3-methylglutaryl coenzyme A reductase|3-hydroxy-3-methylglutaryl coenzyme A reductase|2338
Words > 5|1415615|3-hydroxy-3-methylglutaryl-Coenzyme A reductase|3-hydroxy-3-methylglutaryl-Coenzyme A reductase|2338
Words > 5|279680|transitional cell carcinoma of the bladder|transitional cell carcinoma of the bladder|2290
Words > 5|429706|Forced expiratory volume in 1 second|Forced expiratory volume in 1 second|2149
Words > 5|849974|Forced Expiratory Volume in 1 Second|Forced Expiratory Volume in 1 Second|2149
Words > 5|149925|Small cell carcinoma of the lung|Small cell carcinoma of the lung|2071
Words > 5|243030|Acute Physiology and Chronic Health Evaluation|Acute Physiology and Chronic Health Evaluation|1605
Words > 5|282597|Matrix-Assisted Laser Desorption-Ionization Mass Spectrometry|Matrix-Assisted Laser Desorption-Ionization Mass Spectrometry|1462
Words > 5|282597|Matrix Assisted Laser Desorption Ionization Mass Spectrometry|Matrix Assisted Laser Desorption Ionization Mass Spectrometry|1462
Words > 5|1334094|insulin-like growth factor binding protein 3|insulin-like growth factor binding protein 3|1300
Words > 5|596458|Dorsal Motor Nucleus of the Vagus|Dorsal Motor Nucleus of the Vagus|1214
Words > 5|41711|Department of Health and Human Services|Department of Health and Human Services|1134
Words > 5|376536|PORCINE REPRODUCTIVE AND RESPIRATORY SYNDROME VIRUS|PORCINE REPRODUCTIVE AND RESPIRATORY SYNDROME VIRUS|1119
Words > 5|1516985|European Organization for Research and Treatment of Cancer|European Organization for Research and Treatment of Cancer|1113
Words > 5|20094|Human T-lymphotropic virus, type I|Human T-lymphotropic virus, type I|1091
Words > 5|1334092|insulin-like growth factor binding protein 1|insulin-like growth factor binding protein 1|1044
Words > 5|1150842|ribulose 1,5-bisphosphate carboxylase/oxygenase|ribulose 1,5-bisphosphate carboxylase/oxygenase|1037
Words > 5|20094|Human T-cell leukemia virus type 1|Human T-cell leukemia virus type 1|1012
Words > 5|19351|herpes simplex virus type 2 HSV-2|herpes simplex virus type 2 HSV-2|998
Words > 5|279626|squamous cell carcinoma of the esophagus|squamous cell carcinoma of the esophagus|988

Words > 5|279626|Squamous Cell Carcinoma of the Esophagus|Squamous Cell Carcinoma of the Esophagus|988
Words > 5|19704|human immunodeficiency virus-1 HIV-1|human immunodeficiency virus-1 HIV-1|973
Words > 5|206366|Ossification of the posterior longitudinal ligament|Ossification of the posterior longitudinal ligament|972
Words > 5|279671|squamous cell carcinoma of the cervix|squamous cell carcinoma of the cervix|965
Words > 5|949945|foot and mouth disease virus FMDV|foot and mouth disease virus FMDV|898
Words > 5|278996|Cancer of the Head and Neck|Cancer of the Head and Neck|863
Words > 5|228564|Dorsal Horn of the Spinal Cord|Dorsal Horn of the Spinal Cord|821
Words > 5|149782|Squamous Cell Carcinoma of the Lung|Squamous Cell Carcinoma of the Lung|779
Words > 5|521184|Atypical squamous cells of undetermined significance|Atypical squamous cells of undetermined significance|735
Words > 5|585362|Squamous cell carcinoma of the oral cavity|Squamous cell carcinoma of the oral cavity|728
Words > 5|1522359|Squamous Cell Carcinoma of the Oral Cavity|Squamous Cell Carcinoma of the Oral Cavity|728
Words > 5|342276|Maturity-onset diabetes of the young|Maturity-onset diabetes of the young|721
Words > 5|1515938|American College of Obstetricians and Gynecologists|American College of Obstetricians and Gynecologists|704
Words > 5|27463|National Institute for Occupational Safety and Health|National Institute for Occupational Safety and Health|700
Words > 5|243028|Acute physiology and chronic health evaluation II|Acute physiology and chronic health evaluation II|697
Words > 5|22405|Joint Commission on Accreditation of Healthcare Organizations|Joint Commission on Accreditation of Healthcare Organizations|692
Words > 5|2395|SENILE DEMENTIA OF THE ALZHEIMER TYPE|SENILE DEMENTIA OF THE ALZHEIMER TYPE|658
Words > 5|21141|Syndrome of inappropriate secretion of antidiuretic hormone|Syndrome of inappropriate secretion of antidiuretic hormone|639
Words > 5|80268|Agency for Health Care Policy and Research|Agency for Health Care Policy and Research|637
Words > 5|279671|Squamous Cell Carcinoma of the Uterine Cervix|Squamous Cell Carcinoma of the Uterine Cervix|630
END TOP 50 FREQUENT

RANDOM CASES Words > 5:
Words > 5|1272305|Cerebral autosomal dominant arteriopathy with subcortical infarcts and leukoencephalopathy|Cerebral autosomal dominant arteriopathy with subcortical infarcts and leukoencephalopathy|384
Words > 5|280217|stage, non-small cell lung cancer|stage, non-small cell lung cancer|356
Words > 5|149925|Oat Cell Carcinoma of the Lung|Oat Cell Carcinoma of the Lung|248
Words > 5|1266000|Carcinoma with osteoclast-like giant cells|Carcinoma with osteoclast-like giant cells|75
Words > 5|279680|TRANSITIONAL CELL CARCINOMA OF URINARY BLADDER|TRANSITIONAL CELL CARCINOMA OF URINARY BLADDER|53
Words > 5|278505|stage II non-small cell lung cancer|stage II non-small cell lung cancer|49
Words > 5|949557|Infectious hypodermal and hematopoietic necrosis virus|Infectious hypodermal and hematopoietic necrosis virus|47
Words > 5|42154|Utilization and Quality Control Peer Review Organizations|Utilization and Quality Control Peer Review Organizations|25
Words > 5|345359|Congenital dislocation and subluxation of the hip|Congenital dislocation and subluxation of the hip|20
Words > 5|1332347|Atypical Ductal Hyperplasia of the Breast|Atypical Ductal Hyperplasia of the Breast|16
Words > 5|1333448|Granular Cell Myoblastoma of the Esophagus|Granular Cell Myoblastoma of the Esophagus|15
Words > 5|1334610|Malignant Mixed Tumor of the Parotid|Malignant Mixed Tumor of the Parotid|14
Words > 5|278726|Extensive Stage Small Cell Lung Carcinoma|Extensive Stage Small Cell Lung Carcinoma|13
Words > 5|1335903|Mucoepidermoid Carcinoma of the Salivary Gland|Mucoepidermoid Carcinoma of the Salivary Gland|12

Words > 5|272062|Hemolytic anemia due to pyruvate kinase deficiency|Hemolytic anemia due to pyruvate kinase deficiency|11
Words > 5|1418884|proline arginine-rich end leucine-rich repeat protein|proline arginine-rich end leucine-rich repeat protein|11
Words > 5|1522128|T-cell Lymphomas of the Skin|T-cell Lymphomas of the Skin|11
Words > 5|41709|United States Department of Health, Education, and Welfare|United States Department of Health, Education, and Welfare|9
Words > 5|220620|Carcinoid tumor of the gastrointestinal tract|Carcinoid tumor of the gastrointestinal tract|9
Words > 5|1334643|Adenoid Cystic Carcinoma of the Maxillary Sinus|Adenoid Cystic Carcinoma of the Maxillary Sinus|9
Words > 5|278987|Metastatic Non-Small Cell Carcinoma of the Lung|Metastatic Non-Small Cell Carcinoma of the Lung|8
Words > 5|853221|Very low density lipoprotein cholesterol decreased|Very low density lipoprotein cholesterol decreased|7
Words > 5|349276|Enduring personality change after catastrophic experience|Enduring personality change after catastrophic experience|5
Words > 5|474469|Duration of first stage of labor|Duration of first stage of labor|5
Words > 5|815316|Megaloblastic anaemia due to vitamin B12 deficiency|Megaloblastic anaemia due to vitamin B12 deficiency|5
Words > 5|1336891|Inflammatory Myofibroblastic Tumor of the Bladder|Inflammatory Myofibroblastic Tumor of the Bladder|5
Words > 5|20594|Lack or loss of sexual desire|Lack or loss of sexual desire|4
Words > 5|265932|Congenital anomaly of inferior vena cava|Congenital anomaly of inferior vena cava|4
Words > 5|747273|Malignant Neoplasm of the Parotid Gland|Malignant Neoplasm of the Parotid Gland|4
Words > 5|949132|Loss of teeth due to caries|Loss of teeth due to caries|4
Words > 5|1335100|Occult Squamous Cell Carcinoma of the Lung|Occult Squamous Cell Carcinoma of the Lung|4
Words > 5|1335359|Kaposi's Sarcoma of the Parotid Gland|Kaposi's Sarcoma of the Parotid Gland|4
Words > 5|1335456|Postoperative Spindle Cell Nodule of the Bladder|Postoperative Spindle Cell Nodule of the Bladder|4
Words > 5|1370963|stage B carcinoma of the prostate|stage B carcinoma of the prostate|4
Words > 5|1419123|protein tyrosine phosphatase, receptor type, R|protein tyrosine phosphatase, receptor type, R|4
Words > 5|22672|Acute renal failure with tubular necrosis|Acute renal failure with tubular necrosis|3
Words > 5|26613|Specific developmental disorder of motor function|Specific developmental disorder of motor function|3
Words > 5|30246|Pustular Psoriasis of Palms and Soles|Pustular Psoriasis of Palms and Soles|3
Words > 5|334086|Nevus lipomatosus cutaneus superficialis (Hoffmann- Zurhelle)|Nevus lipomatosus cutaneus superficialis (Hoffmann- Zurhelle)|3
Words > 5|451549|TRISS - Trauma and injury severity score|TRISS - Trauma and injury severity score|3
Words > 5|456368|Working Formulation for Non-Hodgkin's Lymphoma|Working Formulation for Non-Hodgkin's Lymphoma|3
Words > 5|578348|Difficulty getting out of a chair|Difficulty getting out of a chair|3
Words > 5|1413336|carboxyl ester lipase (bile salt-stimulated lipase)|carboxyl ester lipase (bile salt-stimulated lipase)|3
Words > 5|278505|Non-small cell lung cancer stage II|Non-small cell lung cancer stage II|2
Words > 5|338588|Isolated angiitis of central nervous system|Isolated angiitis of central nervous system|2
Words > 5|346302|Growth Hormone Secreting Adenoma of the Pituitary|Growth Hormone Secreting Adenoma of the Pituitary|2
Words > 5|1000331|pepper mild mottle virus PMMV-S|pepper mild mottle virus PMMV-S|2
Words > 5|1000362|Lactobacillus delbrueckii subsp. lactis phage LL-H|Lactobacillus delbrueckii subsp. lactis phage LL-H|2
Words > 5|1185382|Branch of posterior inferior cerebellar artery|Branch of posterior inferior cerebellar artery|2
Words > 5|1274381|Erythema nodosum due to Yersinia enterocolitica|Erythema nodosum due to Yersinia enterocolitica|2

Words > 5|1323367|transforming growth factor alpha receptor binding|transforming growth factor alpha receptor binding|2
Words > 5|1332880|Central Nervous System Germ Cell Tumor|Central Nervous System Germ Cell Tumor|2
Words > 5|1333646|Epidermoid Carcinoma of the Frontal Sinus|Epidermoid Carcinoma of the Frontal Sinus|2
Words > 5|1367859|Pineal parenchymal tumour of intermediate differentiation|Pineal parenchymal tumour of intermediate differentiation|2
Words > 5|1414385|echinoderm microtubule associated protein like 1|echinoderm microtubule associated protein like 1|2
Words > 5|1421339|UDP glycosyltransferase 2 family, polypeptide B17|UDP glycosyltransferase 2 family, polypeptide B17|2
Words > 5|1423701|palate, lung and nasal epithelium carcinoma associated|palate, lung and nasal epithelium carcinoma associated|2
Words > 5|1427860|N-acetylglucosamine-1-phosphotransferase, gamma subunit|N-acetylglucosamine-1-phosphotransferase, gamma subunit|2
Words > 5|1705554|RAS Homolog Gene Family, Member I|RAS Homolog Gene Family, Member I|2
Words > 5|1706913|Basal-Like Subtype of Invasive Breast Carcinoma|Basal-Like Subtype of Invasive Breast Carcinoma|2
Words > 5|13264|mild X-linked recessive muscular dystrophy|mild X-linked recessive muscular dystrophy|1
Words > 5|42029|Urinary tract infection, site not specified|Urinary tract infection, site not specified|1
Words > 5|175696|HYPERTELORISM WITH ESOPHAGEAL ABNORMALITY AND HYPOSPADIAS|HYPERTELORISM WITH ESOPHAGEAL ABNORMALITY AND HYPOSPADIAS|1
Words > 5|278544|Dukes B Carcinoma of the Rectum|Dukes B Carcinoma of the Rectum|1
Words > 5|278733|stage IV cancer of the vulva|stage IV cancer of the vulva|1
Words > 5|318588|Foot-and-mouth disease virus SAT 1|Foot-and-mouth disease virus SAT 1|1
Words > 5|334017|Multiple self-healing epitheliomas of skin|Multiple self-healing epitheliomas of skin|1
Words > 5|339109|Basal Cell Papilloma of the Eyelid|Basal Cell Papilloma of the Eyelid|1
Words > 5|393643|Other degenerative diseases of the nervous system|Other degenerative diseases of the nervous system|1
Words > 5|451910|Erythema marginatum in acute rheumatic fever|Erythema marginatum in acute rheumatic fever|1
Words > 5|458814|Spiral prominence of the cochlear duct|Spiral prominence of the cochlear duct|1
Words > 5|545960|Right fallopian tube and right ovary|Right fallopian tube and right ovary|1
Words > 5|694250|Diltiazem 180 MG Extended Release Tablet|Diltiazem 180 MG Extended Release Tablet|1
Words > 5|716850|Betaxolol Hydrochloride 0.25% Ophthalmic Suspension [BETOPTIC S]|Betaxolol Hydrochloride 0.25% Ophthalmic Suspension [BETOPTIC S]|1
Words > 5|854822|Anaplastic Large Cell Lymphoma Stage I|Anaplastic Large Cell Lymphoma Stage I|1
Words > 5|855114|Diffuse Large B-Cell Lymphoma Stage II|Diffuse Large B-Cell Lymphoma Stage II|1
Words > 5|930563|Superficial part of left parotid gland|Superficial part of left parotid gland|1
Words > 5|1148852|double-stranded RNA adenosine deaminase activity|double-stranded RNA adenosine deaminase activity|1
Words > 5|1150940|pterin-4-alpha-carbinolamine dehydratase activity|pterin-4-alpha-carbinolamine dehydratase activity|1
Words > 5|1152807|hematopoietin/interferon-class (D200-domain) cytokine receptor activity|hematopoietin/interferon-class (D200-domain) cytokine receptor activity|1
Words > 5|1159487|branched-chain aliphatic amino acid transport|branched-chain aliphatic amino acid transport|1
Words > 5|1232196|Chinese squash leaf curl virus SqLCV-C|Chinese squash leaf curl virus SqLCV-C|1
Words > 5|1301363|Early Plasmacytoid Dendritic Cell Leukemia/Lymphoma|Early Plasmacytoid Dendritic Cell Leukemia/Lymphoma|1
Words > 5|1302981|APCT - Alternate prism and cover test|APCT - Alternate prism and cover test|1
Words > 5|1319105|Insertion of internal jugular vein catheter|Insertion of internal jugular vein catheter|1
Words > 5|1320211|Invasive Group A beta-hemolytic streptococcal disease|Invasive Group A beta-hemolytic streptococcal disease|1
Words > 5|1332633|Breast Mucosa-Associated Lymphoid Tissue Lymphoma|Breast Mucosa-Associated Lymphoid Tissue Lymphoma|1

Words > 5|1332762|Cytokine-Inducible SH2-Containing Protein Gene|Cytokine-Inducible SH2-Containing Protein Gene|1
Words > 5|1335213|phosphoinositide-3-kinase, catalytic, beta polypeptide|phosphoinositide-3-kinase, catalytic, beta polypeptide|1
Words > 5|1335413|Benign Glial Cyst of the Pineal Gland|Benign Glial Cyst of the Pineal Gland|1
Words > 5|1336530|Angiosarcoma of the Superior Vena Cava|Angiosarcoma of the Superior Vena Cava|1
Words > 5|1423937|chromosome 2 open reading frame 7|chromosome 2 open reading frame 7|1
Words > 5|1425155|six transmembrane epithelial antigen of the prostate 2|six transmembrane epithelial antigen of the prostate 2|1
Words > 5|1426948|checkpoint with forkhead and ring finger domains|checkpoint with forkhead and ring finger domains|1
Words > 5|1428082|chromosome 9 open reading frame 39|chromosome 9 open reading frame 39|1
Words > 5|1522289|Squamous Cell Carcinoma of the Salivary Glands|Squamous Cell Carcinoma of the Salivary Glands|1
Words > 5|1538476|nucleolar protein with MIF4G domain 1|nucleolar protein with MIF4G domain 1|1
Words > 5|1539758|seizure related 6 homolog (mouse)-like 2|seizure related 6 homolog (mouse)-like 2|1
Words > 5|1603329|Sodium Hyaluronate 10 MG/ML [Healon]|Sodium Hyaluronate 10 MG/ML [Healon]|1
Words > 5|1621673|major histocompatibility complex class II ligand|major histocompatibility complex class II ligand|1
END RANDOM CASES
